# Histology and transcriptomic profiling reveal the dynamics of seed coat and endosperm formation in tree peony (*Paeonia ostii*)

**DOI:** 10.1093/hr/uhac106

**Published:** 2022-05-17

**Authors:** Jing Sun, Haixia Guo, Mi Liu, Ming Chen, Mengyuan Zhu, Datong Liu, Jun Tao

**Affiliations:** Jiangsu Key Laboratory of Crop Genetics and Physiology, College of Horticulture and Plant Protection, Yangzhou University, Yangzhou 225009, China; Jiangsu Key Laboratory of Crop Genetics and Physiology, College of Horticulture and Plant Protection, Yangzhou University, Yangzhou 225009, China; Jiangsu Key Laboratory of Crop Genetics and Physiology, College of Horticulture and Plant Protection, Yangzhou University, Yangzhou 225009, China; Jiangsu Key Laboratory of Crop Genetics and Physiology, College of Horticulture and Plant Protection, Yangzhou University, Yangzhou 225009, China; Jiangsu Key Laboratory of Crop Genetics and Physiology, College of Horticulture and Plant Protection, Yangzhou University, Yangzhou 225009, China; Key Laboratory of Wheat Biology and Genetic Improvement for the Low & Middle Yangtze Valley, Ministry of Agriculture/Lixiahe Agricultural Institute of Jiangsu Province, Yangzhou 225007, China; Jiangsu Key Laboratory of Crop Genetics and Physiology, College of Horticulture and Plant Protection, Yangzhou University, Yangzhou 225009, China

Dear Editor,

In recent years, two species of tree peony have been recognized as potential oil crops, *Paeonia rockii* and *Paeonia ostii* [1]. In 2011, *P. ostii* seeds, which contain 27–33% oil, were identified as novel sources of α-linolenic acid (ALA) for seed oil production in China [2, 3]. Interestingly, peony seed reserves are stored in the endosperm rather than the embryo, but little research has focused on peony endosperm development [4]. Traditional edible oil crops such as rapeseed, soybean, and peanut store oil mainly in the embryo, specifically in the cotyledons. By contrast, tree peony produces oil primarily in the endosperm, distinguishing it from traditional oil crops.

To further investigate this fundamental difference, we performed histological analysis to document the internal structure of *P. ostii* seeds from the pre-storage phase until the end of seed filling. The *P. ostii* embryo only became visible 50 days after pollination (DAP), demonstrating physiological after-ripening much like that of the *Ginkgo biloba* embryo. The *P. ostii* embryo was always surrounded by two covering layers: the endosperm and the seed coat (Fig. 1a).

We sectioned and observed the seed coat by transmission and scanning electron microscopy. We found that the *P. ostii* seed coat underwent a thickening process and stored abundant starch granules early in seed development. At 20–30 DAP, numerous amyloplasts filled with starch granules were observed in the seed coat parenchyma cells, and the parenchyma cell walls were very thin, with a loose appearance (Fig. 1b and c). The cell walls were thinner at this stage than at later stages, which may have promoted cell expansion and nutrient transport (Fig. 1b). By 50 DAP, the number and size of starch granules had increased until they filled most of the seed coat cells, and most starch existed in the form of multiple granules (Fig. 1c). At the same time, the immature endosperm had formed and undergone cellularization (Fig. 1a and e). At 70 DAP, the starch granules had begun to split and degrade, and the density of starch had decreased dramatically (Fig. 1c). The early synthesis and subsequent breakdown of carbohydrates in the seed coat are presumed to be associated with endosperm formation and the accumulation of oil bodies. Severino *et al*. found that variation in seed oil content of castor bean was not associated with total seed weight but instead with the relative weight of the seed coat, shedding new light on the importance of seed coat research [5].

**Figure 1:**
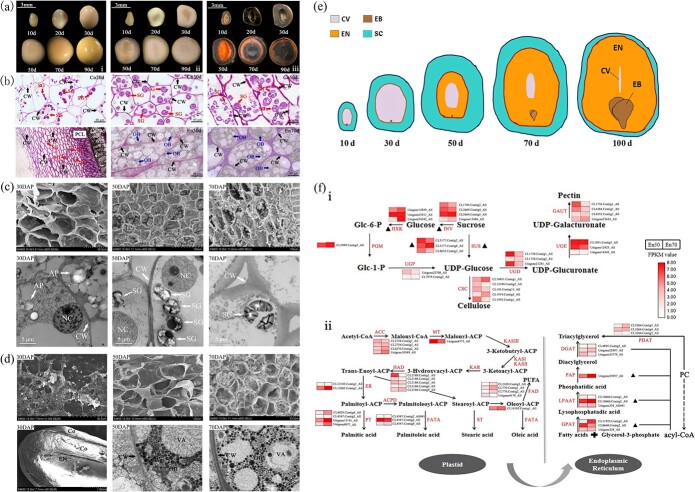
Morphology and histology of the *P. ostii* seed during seed filling and overview of cell wall and lipid biosynthetic pathways during endosperm formation. **a** (i) Stereomicrographs show seed development from 10 to 90 DAP. (ii) Seeds were dissected longitudinally. In early development, the central cavity of the seed was filled with a transparent liquid. A visible endosperm appeared and underwent accelerated cellularization after 50 days. (iii) *P. ostii* seeds were stained with I_2_-KI; dark blue color indicates starch, whereas orange color indicated protein. The seed coat was stained dark blue, demonstrating that it contains starch. **b** Semi-thin sections of the seed coat and endosperm. Starch granules began to accumulate in the seed coat at 20–30 DAP. Starch grains began to disintegrate, and oil bodies gradually accumulated in the endosperm at 50–70 DAP. Scale bar = 40 μm. SG, starch grains; CW, cell wall; OB, oil body; PCL, palisade cell layer of the seed coat. **c** SEM and TEM micrographs of the immature *P. ostii* seed coat during seed filling. Amyloplasts were observed in the seed coat at 30 DAP, and the seed coat cell walls were very thin. At 50 DAP, most starch was present in the form of multiple granules, and the seed coat cell walls had thickened. At 70 DAP, starch granule numbers had decreased sharply, and granules had begun to split and degrade. Scale bar = 5 μm. AP, amyloplast; SG, starch granules; NC, nucleus; CW, cell wall. **d** SEM and TEM observations of the *P. ostii* endosperm at 30, 50, and 70 DAP. At 50 DAP, primary endosperm cell walls were present, and the endosperm cells were filled with oil droplets and vacuoles. At 70 DAP, the endosperm cells had taken on a bouquet-like shape, and their cell walls were clearly thickened. The volume of the vacuole was reduced, and oil droplets were more numerous. Scale bar = 5 μm. Co, seed coat; EN, endosperm; VA, vacuole; CW, cell wall; OB, oil body. **e** Diagram of *P. ostii* seed development. From 10 to 30 DAP, the seed volume gradually enlarged, and the cavity began to fill with transparent liquid. At 50 DAP, the endosperm began to cellularize, oil droplets accumulated, the cavity gradually narrowed, and the starch content of the seed coat increased. From 70 to 100 DAP, endosperm cellularization completed, the endosperm cell walls thickened, oil continued to accumulate, the internal cavity gradually shrank, seed coat starch was gradually degraded, and the embryo developed. CV, seed cavity; EN, endosperm; EB, embryo; SC, seed coat. **f** (i) Expression heatmaps of genes involved in cellulose and pectin biosynthesis pathways during endosperm formation; (ii) Expression heatmaps of genes involved in fatty acid and triacylglycerol biosynthetic pathways during endosperm formation. INV, sucrose invertase; HXK, hexokinase; PGM, phosphoglucomutase; UGP, UDP-glucose pyrophosphorylase; SUS, sucrose synthase; CSC, cellulose synthase; UGD, UDP glucose-6-dehydrogenase; UGE, UDP-d-glucoronate-4-epimerase 6; GAUT, galacturonosyl transferase; ACC, acetyl-CoA carboxylase; MT, malonyl transferase; KASI, β-ketoacyl-ACP synthase I; KASII, β-ketoacyl-ACP synthase II; KASIII, β-ketoacyl-ACP synthase III; KAR-β-ketoacyl-ACP reductase; HAD, β-hydroxyacyl ACP dehydratase; ER, enoyl-ACP reductase; PT, palmitoyl thioesterase; FATA, fatty acyl-ACP thioesterase; ST, stearoyl thioesterase; SAD, stearoyl-ACP desaturase; ACPD, palmitoyl desaturase; FAD, ω fatty acid desaturase; GPAT, glycerol-3-phosphate acyltransferase; LPAAT, lyso-phosphatidic acid acyltransferase; PAP, phosphatidic acid phosphatase; DGAT, diacylglycerol acyltransferase; PDAT, phospholipid:diacylglycerol acyltransferase; PC, phosphatidylcholine. Triangles indicate unigenes that were significantly differentially expressed between 50 and 70 DAP.

We subsequently examined the endosperm cellularization process. Unlike most dicot seeds, which have an ephemeral endosperm, tree peony seeds retain the endosperm throughout seed maturation and can therefore serve as a model system for the study of endosperm development in dicots [5–7]. The *P. ostii* endosperm was not visible until 50 DAP, and the degradation of seed coat starch accompanied endosperm formation. At 30 DAP, primary endosperm cells were not yet visible: beneath the thick seed coat, only an endosperm cavity filled with transparent liquid was present in the center of the seed (Fig. 1d). At 50 DAP, the endosperm began to cellularize gradually from the outside to the inside, and the central cavity steadily filled (Fig. 1a and e). The endosperm cell walls gradually thickened, and the endosperm cells filled with oil droplets and vacuoles (Fig. 1d). At 70 DAP, the endosperm cells had a bouquet-like shape, and the cell walls were clearly thickened. The volume of the vacuole was reduced; it began to take on an oval-like shape, and the number of oil droplets increased (Fig. 1d).

Seed coat development is a complex process during which the integuments of the ovule differentiate into specialized cell types [8]. In most cereals, the seed coat of the grain does not produce starch, and starch accumulates in the endosperm tissue [9]. To date, few studies have reported on the relationship between seed coat development and seed oil content. Here, we showed that the *P. ostii* seed coat is a storage location, as it thickens and accumulates starch during the early stage of seed development (Fig. 1e). As the endosperm develops, the starchy seed coat gradually decomposes and is compressed towards the outside of the seed. These results are consistent with an important role for the *P. ostii* seed coat during endosperm filling and oil accumulation.

To identify potential target genes that control final seed traits, we next analyzed the seed coat and endosperm transcriptomes separately at key seed developmental stages (30, 50, and 70 DAP) (NCBI BioProject PRJNA648679) (Supplementary Data Tables S1–S3; Supplementary Data Figs S1 and S2). Ten genes were randomly selected for qRT–PCR validation, and transcriptome data were consistent with quantitative results (Supplementary Data Fig. S3; Supplementary Data Table S4). Starch metabolism genes were among the most highly regulated genes in the *P. ostii* seed coat. Genes related to cell wall metabolism, starch synthesis, sucrose mobilization, and lipid synthesis were upregulated at 50 DAP compared with 30 DAP (Table S5). We identified two types of starch synthase genes in the seed coat, *granule-bound starch synthase* (*GBSS*) and *soluble starch synthase* (*SSS*), and we found that *GBSS* (*CL2717.Contig6*, *Unigene21772*) and *SSS* (*CL7708.Contig1*) expression levels were among the highest of any annotated unigenes. Other starch synthesis-related genes were also identified, including *glucose-1-phosphate adenylyltransferase*, *1,4-alpha-glucan-branching enzyme*, and *amyloplastic precursor*. Comparisons of differentially expressed genes (DEGs) between 30 and 50 DAP and between 50 and 70 DAP indicated that starch degradation-related genes were expressed at increasing levels throughout development. Genes encoding both α-amylase (*Unigene11173, CL3562.Contig5, CL6483.Contig2*) and β-amylase (*CL10393.Contig2, CL6765.Contig3, CL6765.Contig4*) continued to increase in expression from 30 to 70 DAP. The endosperm developed rapidly from 30 to 70 DAP, and the expression of genes related to starch degradation in the seed coat was presumably tightly correlated with rapid endosperm formation, as starch serves as the carbon source for oil production in the endosperm (Supplementary Data Table S6).

Endosperm cellularization is accompanied by the formation of cell walls. Plant cell walls are constructed from cellulose, hemicellulose, pectin, and other quantitatively minor but functionally important components [10]. UDP-glucose is the building block for cellulose and pectin synthesis, and the *P. ostii* endosperm consists of sink cells in which sucrose is catabolized to produce UDP-glucose via sucrose synthase (SUS) or intracellular invertases (INVs). Our transcriptome contained three *SUS* unigenes, among which *CL5177.Contig6* had the highest expression and continued to increase in expression from 50 to 70 DAP. Most genes encoding invertase (INV), hexokinase (HXK), and phosphoglucomutase (PGM) also increased in expression during endosperm cellularization. Genes related to pectin synthesis included *UDP glucose-6-dehydrogenase* (*UGD*), *UDP-D-glucoronate-4-epimerase 6* (*UGE*), and *galacturonosyl transferase* (*GAUT*); their expression levels were all higher at 50 DAP than at 70 DAP [Fig. 1f(i); Supplementary Data Tables S7 and S8].

Endosperm cellularization is also accompanied by the accumulation of triglycerides. Here, endosperm gene expression patterns suggested that fatty acid synthesis pathways were active, and the increased expression of *SAD* and *FAD* genes was consistent with an increase in the synthesis of polyunsaturated fatty acids (PUFAs). As more fatty acids were presumably transported to the endoplasmic reticulum, most of the unigenes encoding the enzymes glycerol-3-phosphate acyltransferase (GPAT), lyso-phosphatidic acid acyltransferase (LPAAT), phosphatidic acid phosphatase (PAP), and diacylglycerol acyltransferase (DGAT) showed increased expression, suggesting that the endosperm would continue to accumulate oil after 70 DAP [Fig. 1f(ii)].

Our findings provide novel insights into the dynamics of the developing seed coat and endosperm in *P. ostii* from histological and transcriptomic perspectives. We propose that sucrose–starch interconversion probably occurs in the developing seed: a portion of unloaded sugar is stored temporarily in the seed coat cells as starch, thereby providing an energy source for oil accumulation in endosperm.

## Data availability

Data are contained within the article or Supplementary Data files.

## Conflict of interest

The authors declare no conflict of interest.

## Supplementary data

Supplementary data is available at *Horticulture Research* online.

## Supplementary Material

Web_Material_uhac106
