# Supplementary material for: Histology and transcriptomic profiling reveal the dynamics of seed coat and endosperm formation in tree peony (Paeonia ostii)
Source: Hortic Res. 2022 May 17;9:uhac106. doi: 10.1093/hr/uhac106 (PMC9297151; doi:10.1093/hr/uhac106)
Supplement: Web_Material_uhac106 [file web_material_uhac106.zip › Supplementary figures.docx]

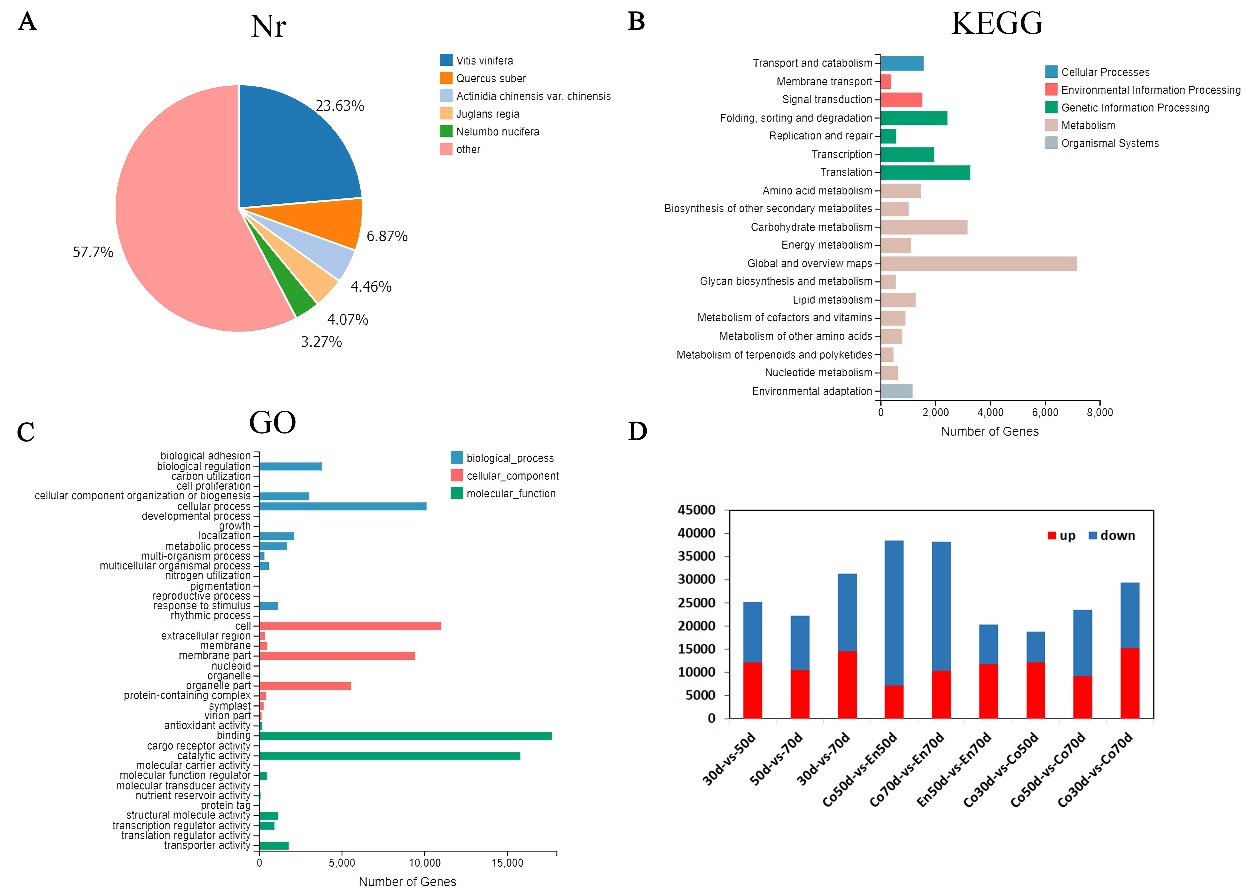


Figure S1 Functional annotation of unigenes. A Top blast hit species in the non-redundant protein database (Nr). B. KEGG annotations of unigenes. C GO annotations of unigenes. D Differentially expressed genes in different sample comparisons. Co, seed coat. En, endosperm.


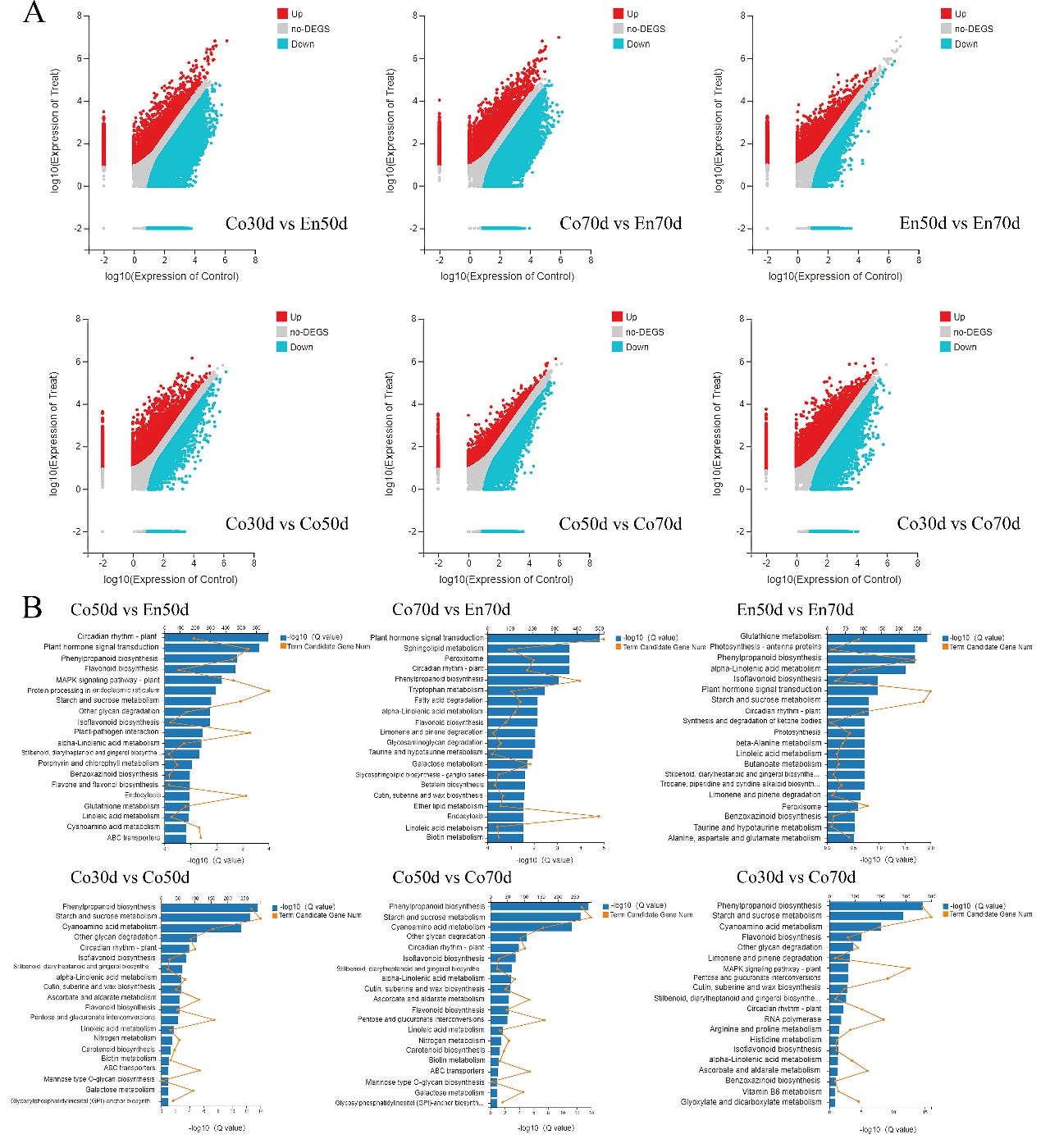


Figure S2 Differentially expressed genes in the seed coat and endosperm of *P. ostii* at different developmental stages. A Scatter plot of upregulated and downregulated genes between seed samples from different developmental stages. Red and blue points indicate up- and down-regulated genes. B KEGG pathway enrichment of DEGs. The enrichment levels of the top 20 enriched KEGG pathways are displayed as blue bars, and the numbers of DEGs in each pathway are shown with yellow points. Co30d, Co50d, Co70d, En50d, and En70d represent the *P. ostii* seed coat (Co) and endosperm (En) at 30, 50, and 70 DAP.


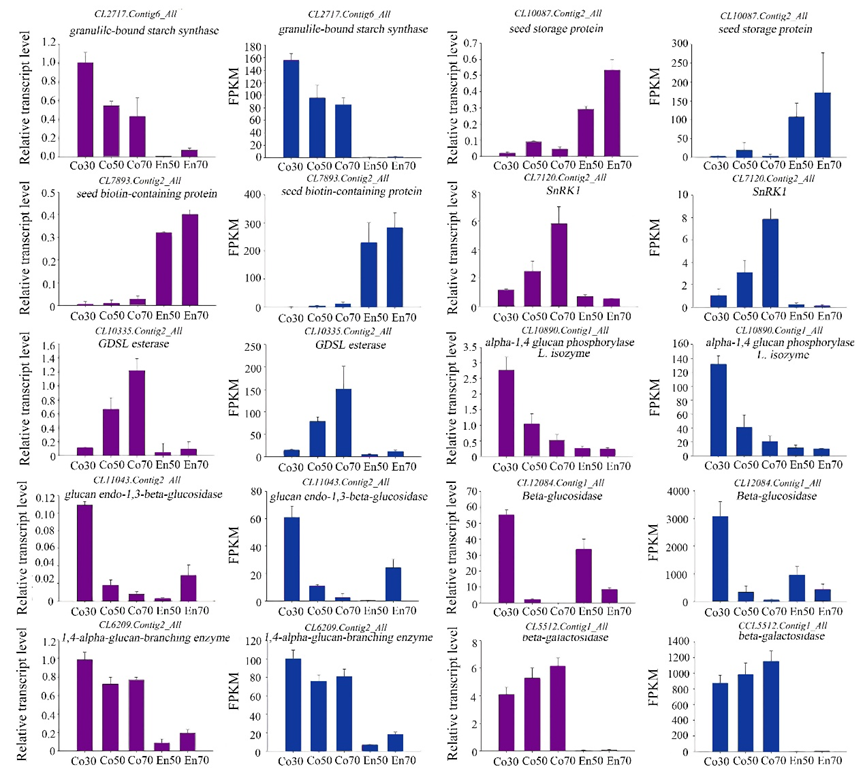


Figure S3 qRT-PCR verification of RNA-sequencing data for ten randomly-selected genes associated with seed development.
