# Supplementary material for: Histology and transcriptomic profiling reveal the dynamics of seed coat and endosperm formation in tree peony (Paeonia ostii)
Source: Hortic Res. 2022 May 17;9:uhac106. doi: 10.1093/hr/uhac106 (PMC9297151; doi:10.1093/hr/uhac106)
Supplement: Web_Material_uhac106 [file web_material_uhac106.zip › Table S1.docx]

Table S1 Summary of RNA sequencing data from *P. ostii* seeds

| Sample | Raw Reads (Mb) | Clean Reads (Mb) | Clean Bases (Gb) | Clean Reads Q20 (%) | Clean Reads Q30 (%) | | Clean Reads  （%） | |
| --- | --- | --- | --- | --- | --- | --- | --- | --- |
| Co30-1 | 71.88 | 68.05 | 10.21 | 98.37 | | 94.19 | | 94.68 |
| Co30-2 | 71.88 | 68.65 | 10.3 | 98.6 | | 94.81 | | 95.52 |
| Co30-3 | 70.24 | 67.18 | 10.08 | 98.63 | | 94.89 | | 95.64 |
| Co50-1 | 71.88 | 68 | 10.2 | 98.42 | | 94.31 | | 94.6 |
| Co50-2 | 71.88 | 68.37 | 10.26 | 98.53 | | 94.62 | | 95.12 |
| Co50-3 | 71.88 | 68.45 | 10.27 | 98.59 | | 94.77 | | 95.23 |
| Co70-1 | 71.88 | 67.14 | 10.07 | 98.27 | | 94.01 | | 93.41 |
| Co70-2 | 71.88 | 67.44 | 10.12 | 98.44 | | 94.39 | | 93.83 |
| Co70-3 | 71.88 | 67.34 | 10.1 | 98.28 | | 94.04 | | 93.69 |
| En50-1 | 70.24 | 67.44 | 10.12 | 98.79 | | 95.26 | | 96 |
| En50-2 | 70.24 | 67.21 | 10.08 | 98.69 | | 94.97 | | 95.68 |
| En50-3 | 66.43 | 62.82 | 9.42 | 98.72 | | 95.1 | | 94.57 |
| En70-1 | 71.88 | 68.32 | 10.25 | 98.5 | | 94.44 | | 95.06 |
| En70-2 | 71.88 | 68.08 | 10.21 | 98.49 | | 94.44 | | 94.72 |
| En70-3 | 71.88 | 68.05 | 10.21 | 98.62 | | 94.73 | | 94.67 |
| Average | 71.19 | 67.50 | 10.13 | 98.53 | | 94.60 | | 94.83 |
