# Supplementary material for: Histology and transcriptomic profiling reveal the dynamics of seed coat and endosperm formation in tree peony (Paeonia ostii)
Source: Hortic Res. 2022 May 17;9:uhac106. doi: 10.1093/hr/uhac106 (PMC9297151; doi:10.1093/hr/uhac106)
Supplement: Web_Material_uhac106 [file web_material_uhac106.zip › Table S4.docx]

Table S4 Primers used for qRT-PCR

| Gene ID | Gene name | Forward primer (5' - 3') | Reverse primer (5' - 3') |
| --- | --- | --- | --- |
| CL2717.Contig6_All | granule-bound starch synthase | TACAAATGAGAACCCGTGC | CAAAGACCAGGTTCATCCC |
| CL10087.Contig2_All | seed storage protein | CAAGACCAAAGCCACCATC | TGTTCAGTTCTGCCTCGTTAG |
| CL7893.Contig2_All | seed biotin-containing protein | GAAGGGTGATACTGCCAAA | CTTTCTCGGTCGTGTAATG |
| CL7120.Contig2_All | SnRK1 | GGTTATTTTCACATCGTCAC | GTCTGCCGTTCTCAGTAAT |
| CL10335.Contig2_All | GDSL esterase | TCAAGAACAAAGGACACCC | TGCCATCCCTCTTGCGTAG |
| CL10890.Contig1_All | alpha-1,4 glucan phosphorylase L isozyme | ATTTGTGAGGAGCGGTGTT | GGTCTCCATAAGCCTCGTC |
| CL11043.Contig2_All | glucan endo-1,3-beta-glucosidase | CCGCTTACCACCCATCAAC | TGAAGACGGGTAGGAGTTT |
| CL12084.Contig1_All | Beta-glucosidase | GGTGAAGATGATAAGGCTA | GTCCCAGAGTCTATGTGCT |
| CL6209.Contig2_All | 1,4-alpha-glucan-branching enzyme | TTCATTCCTCCTCCTGGCA | TAAGTTACACCTGTGGGAC |
| CL5512.Contig1_All | beta-galactosidase | CAACAGGCGTCTCATCCGT | CCTTTCCCATACTTCGCAT |

|  |
| --- |
