# Supplementary material for: Histology and transcriptomic profiling reveal the dynamics of seed coat and endosperm formation in tree peony (Paeonia ostii)
Source: Hortic Res. 2022 May 17;9:uhac106. doi: 10.1093/hr/uhac106 (PMC9297151; doi:10.1093/hr/uhac106)
Supplement: Web_Material_uhac106 [file web_material_uhac106.zip › Table S7.docx]

**Table S7 Distinct data on cell wall metabolites during endosperm formation**

| Gene ID | Gene name | En50 FPKM | En70 FPKM |
| --- | --- | --- | --- |
| CL5177.Contig2_All | SUS | 7.40 | 5.00 |
| CL5177.Contig6_All | SUS | 6.00 | 8.00 |
| CL8635.Contig2_All | SUS | 4.99 | 2.71 |
| CL1705.Contig3_All | INV | 4.80 | 3.35 |
| CL5669.Contig4_All | INV | 6.06 | 5.00 |
| CL5669.Contig5_All | INV | 7.20 | 8.00 |
| Unigene13486_All | INV | 1.59 | 2.46 |
| Unigene12859_All | HXK | 4.38 | 6.10 |
| Unigene23911_All | HXK | 8.00 | 8.00 |
| Unigene24242_All | HXK | 1.21 | 8.00 |
| CL2999.Contig5_All | PGM | 6.80 | 7.80 |
| Unigene22708_All | UGP | 2.63 | 1.33 |
| CL7879.Contig2_All | UGP | 0.80 | 0.80 |
| CL10451.Contig1_All | CSC | 3.22 | 5.45 |
| CL12349.Contig2_All | CSC | 2.54 | 1.04 |
| CL143.Contig15_All | CSC | 3.04 | 1.79 |
| CL1919.Contig3_All | CSC | 1.64 | 3.53 |
| CL1952.Contig1_All | CSC | 5.03 | 1.55 |
| CL1738.Contig1_All | UGD | 7.96 | 2.81 |
| CL1738.Contig2_All | UGD | 1.42 | 1.75 |
| Unigene21241_All | UGD | 6.90 | 2.53 |
| CL1051.Contig5_All | UGE | 8.00 | 8.00 |
| Unigene12925_All | UGE | 7.40 | 5.60 |
| Unigene14343_All | UGE | 0.96 | 0.86 |
| CL1736.Contig1_All | GAUT | 6.00 | 1.34 |
| CL4384.Contig1_All | GAUT | 4.25 | 2.25 |
| CL4552.Contig1_All | GAUT | 1.67 | 2.27 |
| Unigene23624_All | GAUT | 2.62 | 3.87 |
