# Supplementary material for: Histology and transcriptomic profiling reveal the dynamics of seed coat and endosperm formation in tree peony (Paeonia ostii)
Source: Hortic Res. 2022 May 17;9:uhac106. doi: 10.1093/hr/uhac106 (PMC9297151; doi:10.1093/hr/uhac106)
Supplement: Web_Material_uhac106 [file web_material_uhac106.zip › Table S8.docx]

Table S8 Nr annotations and sequences of genes mentioned in the manuscript

| Gene ID | Nr | Sequence |
| --- | --- | --- |
| CL2717.Contig6_All | PREDICTED: granule-bound starch synthase 1, chloroplastic/amyloplastic-like isoform X1 [*Juglans regia*] | ATGGCAACTGTGACTGCTTTACACTTTGTATCAAGGAGCTCACATGGCAACTGCCAAGGAACTTTGGGATCAGAAACTAAAGCAGCTGTGGGAAACTTGGGTTTAAGGAATCAAACCATATCACACAATGGGTTAAGGTCATTAAACACGGTAGATATACAAATGAGAACCCGTGCAAAAGCAATTCCCGGGCAAGTGAGGGCAAATGGGTACAAAAATGAAAATGGCAGGCATTCTGGAGCGATTATATGTGGAAATGGGATGAACCTGGTCTTTGTAGGAGCTGAAGTTGGTCCATGGAGCAAAACCGGAGGACTCGGTGATGTTCTAGGGGGACTGCCACCAGCCATGGCGGCGATTGGGCACCGTGTTATGACAGTTTCTCCACGATATGACCAATACAAAGATGCATGGGACACTGAAGTACAAGTTGAGATAAAACTTGGAGATAAAATGGAAAATGTTCGTTTCTTCCACTGCTATAAACGAGGAGTTGATCGCGTCTTTGTGGATCACCCGATGTTCCTTGCAAAGGTATATGGAAAAACTGGATCTAAAATATATGGTCCTAAGGCAGGAAAGGATTATGAGGATAACCAACTTCGTTTCAGCTTGCTGTGCCAGGCTGCTCTGGAGGCACCAAGGGTTTTAAATCTAAACAGCAATAAATATTATTCAGGACCATATGGAGAGGAAGTTGTCTTCATTGCAAATGATTGGCACACTGCTCTTCTTCCATGTTACCTGAAAACTATGTACAAATCTAGGGGGATCTACAAAACTGCCAAGGTTGCTTTCTGTATCCACAACATGGCTTACCAAGGCAGATTTCCCTTTCCAGACTTCTCACTTCTTAATCTGCCTGATCAATTTAAAAGCTCCTTTGATTTTATTGATGGTTACAACAAGCCTGTGAAGGGGAGGAAAATAAATTGGATGAAGGCTGGAATATTAGAATCAGACAGGGTTGTAACTGTGAGCCCATACTATGCCCAGGAACTAGTTTCTGGCGAAGATAAGGGTGTGGAATTGGATAACATCATTCGTAAAACTGGCATAACTGGTATTGTGAATGGCATGGATGTCCAGGAGTGGAACCCATCCAAAGACAAATACATTGATGTTAAATATGATACCACAACTGTGATGGATGCAAAGCCCCTATTGAAAGAAGCCCTACAAGCAGAAGTTGGGTTGCCTGTAGACAAGAATATCCCTTTAATAGGCTTCATTGGTAGACTAGAAGAGCAGAAGGGTTCAGATATACTGGTGGAAGCCATTCCAGAATTTATTGGGGAGGATGTTCAGATAGTAGTCCTTGGAACTGGCAAAAAACTTATGGAAAAGCAGATTGAGCAACTAGAGATATTGTATCCAGACAAGGCTAGAGGAGTGGCAAAATTCAATGTCCCATTGGCTCATATGATTATTGCTGGAGCTGATTTTATGTTGATTCCAAGTAGATTTGAACCATGTGGTCTGATTCAGCTGCATGCTATGCGCTATGGAACGGTGCCCATTGTTTCATCAACCGGTGGACTTGTTGACACTGTCAAAGAAGGTTTTACCGGATTCCAGATTGGAGCCTTCAGCGTTGAATGTGATGAAGTCGATCCAGCCGATGTAA |
| >Unigene21772_All | PREDICTED: granule-bound starch synthase 2, chloroplastic/amyloplastic-like [*Juglans regia*] | ATGTTCCCGTTTTCAAATTCCCAGCTTATTTGGACCTCATAACAACTCTCTATGATTTTCCATTCCGTGGGACCCGTCTCAACAAATGGCGTCCGTGGGATCTCTTCCTTTTACCATTGAAATGAGGTCAGAGAGTTCTGCACTTCTGAACTGTGGGAAGAGTCGACCCAGGTTTCGTTTCTTGGCCTATCAACCGCGGAAGTCGCTTGAATGCGCTGATTTGAATGATTCAACATTTGGTTATTTAAAAGCTGTGGGAAATAATACTGCGATGTATACTCTATTCCCCGAGAGAGTAATAATTGGGTCCAATAGAGGGAGGATGATTAAGCATCTGAGAGCAATAGGCGATGACTCTGTTGAACGGGAGAGCGGCGATGACTCAGAGGAGGCGCTTCAGGCTACAATTGAGAAAAGCAAGAAGGCTCTTGCTATGCAGAGAGATCTACTTAAACAGATTGCTGAAAGAAGGAAATTGGTTTCATCAATAAAAAGTAGTGTTATTGATCCAAAAGATGATGAAGTTTCCTATGAGGAAAGGGAAAATTCTGCGCAAAATCCAGGTCTTGCTTCTAGTGGTGATGATGGTATTAACAAAGAGTCTGATAATGACATTCATTCTAGTAGCTATGTTCATTCAACTGCAAATGAGGAGCCTGAAACCCTATCTCCAGCCAGTAGAGGTTTTGGCAAAGGCAAGAAGGAACCTGGAAAGAGCTTAACTCATGACAAGGCTTCCTTGGACAAAACAGATTGGTCTGATAGACTGCCATCTATTATTCCAAATTCCTCCAAGGTTTTAAGTGTGAATGGTGAAAAGCATGAAGTGTTTATAGAAAAAACTTTGCAAGAGTTGAATAGTGAGGCAAATGCTCCCATCATTGAAGATGTAAAATCTCCTCCTTTGGCTGGAGCCAATGTAATGAATGTTATATTGGTAGCAGCAGAATGTGCTCCTTGGTCTAAAACAGGTGGTCTTGGAGATGTTGCTGGGGCTTTACCGAAGGCTTTGGCTCGGCGTGGACACAGGGTTATGGTTGTGGCACCTCGGTATGACAATTATAACGAATTCAATGACACAGGAGTTCGGAAAAGGTATAAGGTGGATGGGCAGGATGTGGAAGTAAGTTACTTCCAGACCTATATTGACGGCGTGGATTTTGTTTTTATTGAGAGTCCCATGTTTCGCCATATAAATAATGATATATATGGAGGAAAACGAGAGGATATTTTGAAACGCATGGTTTTATTTTGCAAGGCAGCTGTTGAGGTTCCTTGGCATGTCCCTTGTGGTGGTGTCTGCTATGGAGATGGAAATTTGGCTTTCATTGCAAATGATTGGCATACTGCATTGTTGCCCGTGTATCTGAAGGCATATTATCGTGATAATGGATTAATGAAATATACAAGGTCTATTCTTGTAATACATAACATAGCTCACCAGGGTCGTGGTCCAGTGGATGATTTCAGATATGTGGATCTGCCACCACACTACATAGACCTTTTCAGACTGTACGACCCTGTTGGAGGTGAGCATTTTAATATCTTTGCTGCTGGTCTAAAAGCAGCAGATCGTGTGGTCACTGTTAGTCATGGATATGCATGGGAGCTCAAAACTTTAGACGGTGGTTGGGGTTTGCACGGGATCATAAATGATAGCGATTGGAAACTGAGAGGTATTGTAAACGGAATCGACATGAAAGATTGGAATCCGCAGTTCGATGTTTTCTTGACTTCTGATGGTTATACTAACTACTCCCTCGAGACTCTTCAGACTGGCAAACCCCAATGCAAGGCAGCGTTACAAAAGGAGCTTGGTTTACCCATTCGAGAGGATGTCCCGTTGATTGGTTTCATTGGCAGGCTGGATTATCAGAAAGGTGTAGATATCATAGCTGAGGCAATTCCGTGGATGATGGGACAGGACGTGCAACTAGTCATGTTGGGCACAGGGAGAGACGACCTGGAACAGATGCTCAGACATTTTGAGAACCAACACCGTGACAAAGTCCGGAGCTGGGTTGGTTTTTCTGTACAGATGGCTCACCGTATTACTGCAGGTTCAGACATTTTGCTCATGCCATCACGATTTGAACCTTGTGGACTGAACCAACTGTATGCAATGAGTTATGGGACAGTTCCTGTTGTACATTCTGTTGGTGGACTGAGAGACACTGTGCATCCTTTCAATCCGTACGAAGAATCAGGGCTTGGGTGGACATTTCATAGGGCTGAGGCGAGTCAGCTGATACATGCACTGGGAAATTGCATATATACTTATTGGGATTTCAAGAATAGTTGGGAAGGGATTCAGAGACGTGGAATGATGCAAGACCTTAGCTGGGATAA |
| CL7708.Contig1_All | soluble starch synthase 1, chloroplastic/amyloplastic [*Quercus suber*] | ATGGAGTCTCTGCAGATATCACGTATTACTACCTCTCCTCCAAAGTTAGCAGAAATTACTACTAATTTTAGAGTATTTAAGCAGTTGGGTTTTGTTCCTTGGTGGAGGCAGAGTAGTAGAACTGGGTCTTTGTGCGTTAGAAGATCAGTTTCTGGTAGGAAAAAAGGTGGAATTGGTAGTTCACGCGATCGTTCTTCTGCTGTCGAAGATGAAAAGGAGAGTCTTTTATTGGGCACAGAGAGAGATGGTTCTGGCTCCGTTATTGGGTTTCAATTGACTCCTCAATCCGAGCAAAAAGAAATGTCTTCAACAGCTGACTCAGAAATCAATGATTCTCATGAAAACATAGCTACTGGCAGAGAAGACGAATCTGAGGACATTGAAGGAGAAGAGAATATCAAATCTAAAGTAACCCGCAATATTGTTTTTGTTACTTCTGAAGCAGCACCATACTCAAAGACAGGAGGTTTAGGAGATGTTTGTGGTTCTTTACCTATAGCATTAGCTGCACGTGGACACCGTGTAATGGTGGTCTCTCCTAGGTATTTAAATGGTACTTCTTCAGATAAAAAGTTTGCTGGTGCATCTGATGCTAACTGTCGCGTTGAGTGCTACTGCTTTGGAGGGGTACAGAAGGTTGCCTTATTCCATGAATACAGGGAAGGTGTTGATTGGGTATTTGTGGACCATCCATCGTATCGTCGACCTGGAAATCCATATGGTGATAGTCATGGTGCTTTTGGTGATAATCAGTTCCGGTTCACCTTACTTTGCCATGCAGCATGTGAAGCTCCATTGGTGCTTCCATTGGGGGGGTTTACCTATGGAGAGAAATGCCTGTTCCTGGTTAATGATTGGCATGCAGGCCTTGTGCCAGTATTGTTGGCGGCCAAGTATCGTCCATATGGAGTTTACAAGGATGCTCGAAGTATTCTCGTTATACATAACCTTGCACATCAGGGGGTGGAGCCTGCAGTAACTTACAAGAATTTGGGATTGCCTCCCGAGTGGTACAAGGCCCTAGAATGGGTGTTCCCCACGTGGGCAAGGACGCATGCTCTTGACACAGGTGAAGCGGTTAATGTTCTGAAGGGCGCGATTGTGACAGCGGACCGAATACTGACAGTTAGCCAGGGCTATTCTTGGGAAATAACAACTGTTGAAGGTGGATATGGTCTAAATGAATTATTAAGTAGTCGAAAGATCGTCTTGAATGGGATCACAAATGGCGTTGATGTTACTGAGTGGGACCCATCTTCAGATGAGCATATTGATTTCCACTACTCTATCCATGATCTTTCTGGAAAGGTTCAGTGCAAGATGTCTTTGCAGAAGGAATTGGGTCTTCCCGTTAGGCCAGAGTGTCCATTGATTGGATTTATTGGGAGATTGGACTACCAGAAAGGCATCGATATTATTCGGATATCAATTCCAGAGCTTATGCAAGATGATGTTCAATTTGTTATGCTCGGGTCAGGAGATCCATTGTACGAAGATTGGATGAGAGCAACAGAGGCAACATATAGAGACAAATTCCGGGGTTGGGTTGGATTTAACGTTCCAATTTCTCATAAAATAACTGCTGGCTGTGATATACTATTGATGCCCTCAAGGTTCGAACCTTGTGGGTTAAATCAACTGTATGCAATGAGATATGGAACTGTACCAGTAGTTCATGGCACTGGAGGACTTAGGGATACAGTAGAGACCTTCAATCCATATGCTCGAGAAGGCAGTGGTGAAGGCACTGGGTGGACTTTTTCTCCATTATCAAAAGAGAGTATGTTGACGGCACTAAGACTTGCTGTTGGAACTTATAAAGAGCACAAGTCTTCATGGGAGGGATTGATTAA |
| Unigene11173_All | PREDICTED: alpha-amylase [*Vitis vinifera*] >CBI32409.3 unnamed protein product, partial [*Vitis vinifera*] | ATGATGATTCACGTACAAGGGCCGAAAAACCAAAGCAAGCTATAATAAACATAGTTGAGTAGAACTCTACACATTCTTCTCCCAAACAGCATAATTATTTCCAGAAGTAGAAACTTTGAAACCGGGTGGAATAAGACTCCCAACATTAAACTTTGGCCCAATTTTGGCAATGATTTTCTGGTCTATTATTGCTACATAGAGATCCGAATCGGCTGCTAGAATTTGCAAGTGACTGTTGGGATTGATCCCGTTCTTGGTTCTGATTGCAATCAGCTTAGAAATCTCAGCGCTCAAACCCCAGTCAAAGAAGTGATCATAGAAAATGGATGGGATCCCTGGATGGGTGAGAATATAAGCATATCCCTGCATGACTTTATCTTTAGGGAATGGCCACATATTTTGTGTAGAACCAGTGTCATGATTATCAATGAAAGTAACAGCGTTGGCGGGTAAAGAGCCAATCATTCCTGGAGGTTTTCCATTTGCATCTTTCAACCTCCCCAGCTCTCCTTGAACAGCAGCTTGAAGAATGCCTTTGGTCGTAAAATCAAAAGCAGTCACCTTACCACCGGCAGCTTTTACCCAATTCACAAGGTTTCCCCTATGCACATCCTGGTTATAATCTGGCTTCCCATCACTTCCATAAGCAAGATCATTCCAAATTTCCCCAACTGCAAAAGTTGGATTGGTGTTTTGCACATACAACTTGGTATAATCTGGGGCATACCCTTTGGAGAAGTCGAATCGCCATCCATCAAATCCAATATCAGTCTTCAACCAATTCATCCAATCGGATAGCTCTCTCTGGACTCGCGAGTTTACGTGGTCAATGTCTGGAGCAGCAGCAAAGCCTGCTCCGGTATCAGGGTTCCCTGTACCATCAGAATATTGAACATCATCTTTGCAGATAAAGGATGGACCCCAATCCAGGCGATCATCTGGAGTTCCTCCTTCAAAGATGCAATATATTCCTCTTGAGTCTTTCCTTTCTGCTGTTCTGTGGTTTATTACTATGTCTGCTATGCACTGGATCTTATTGTCATGAAGGGTGCTTATTAGTGCTCTTAAGTCAGCAGAGCTTCCATATTTCGATGAGTCAAGGTCATATAGCCTCCCTGGCATATACCCATCAGGTGAAACAGACTGGGAAGCTGGAGGAAGCCATACATGTGTAATGCCAGAAGCTGCCAGTTGAGGAACAGAGTTTTTCAAGATATTATACCAACCTCCTTTTTTCGATGACTCCCAGTTGAAGCCCTGAAATAATATTTGAGAAGTTGTAAAGTTGGGTAGGACAGAGACGAAGAGTAGGAGAAAACAGAGAGAATGGATAA |
| CL3562.Contig5_All | alpha-amylase [*Paeonia suffruticosa*] | ATGTTTATTCATGCAATTGGTTCCGGGCATTACAATAATTCCAATGGAAAACTGGAAACCGGATGTTTTATACATACAAGACTATTATTATATTGTAGCTCCTAAGAGCAATACTGTTATTCTAGAGAAACTGCTTCTCCCACACTGCATATCTGTGGCTGATGGTCGCGAGTGTCCACTCTCTACCGGCTGGACACCATGAACCGGATGTTTTATACATACAAGACTATTATAATAGAGTAGCTCCTAAGAGCAATATTGTTATTCTAGATATAGAGAGAAACTACTAGCTATAGAGAGAAACTACTTCTCCCACACTGCATATCTGTGGCCGCTGGTCGCGAGTGTCCACTCTTTACCGGCTGGACACCATGAACCATCCCCAATTTTCATTGAAACATTCTCTCCAATTATTGCAGCATACAGATTCGGCTGGGCCTCTGCAATTTTTATTGAGGATCGGCTGTGTAAGTCTTGATGCTTCCGAATATCAATCAGCTTCACAATTTGATCTTGAGTGGAGTTGCCCCAATCGTAAAAGTGGTCGTAAAAAACGGATGGAATCCCAGGATGTGTGAGTATATATGCATAGCCCTCCATTATATTATTGGAAGGGAAGGGCCAATGAGCCTGTGTTGAGCCAGTATCATGGTTGTCAAGGAATGTAACCGACCTTGAAGGCCACCAGCCCATTACACCAGGTGGCTTCCCTTGCCCATCACGCAGACGCCAGAGTTCCCCTTTAACAGCTTCCTGAAGAATTCCCTTGGTTGTGAAGTCAAATGCTGTTGAAAGCTGACCTGTGCCATCAATCCAATTGATAATCCGCTGTCTATGACTATCTTGGTTGTAATCCAAGTGGTGACCATTATAGTTGCAAGAATCCCAATACTCTCCAACAGAAAATACCGGCTTTGCCCCTTCAATGTACTCTTGCACGTATTTTGCTGAATAACCTCTTGCAAAATCAAAACGAAAATCTTGGAAGCCAACACTATTTCGTAGCCATCGTAGCCACTCTATAATGTCTTTTCGAACAAAATGTTGAGAATGATCAATATTTGGAACACCATTAAAGTTGTCACCAGTGCTTTTATTACCCAGGCCACCAGTACAAGATGTGACAGCACGTTCATCCCATGATAACGGAATTCCATCATATCGGTTATACATTCCCCCGTGCCCTCGAGTAGTCCCAACACGATGATTGATTACTATATCAGCCATTGCTCTAACTTTGTACTGCTTCATTTTTTGAAGTAAAGCTCTTAAAAGGTGCTCAGAACCATATGAAGAATTGAGGGAATAAATGTTCTGTGGAAGGTAACCTTGAGGTGCAAGAGAATGAGATGCCGGTGGCAACCAAGCTGATGTAAACCCAGATTTTGCAATATCAGGAACCTTCCTTTCTAAATTGCTCCACCAATCATATTTATGGGACTCCCAGTTGAAAGCCTGCAAAAGGATTTCTCTTCCACTGCGTAGTACTGCACCAGGATCAGTCTGCTGAGGAGAATCTTCATGCTCATGGTTCAGGTTACTCATATCAGATCACAGGATTGTTTACCAAAATTTGACGCTGGATCAAAACAAAAGAAAGGCACGTAGACCTATCCGCAGATAAAGTTCTCGTTAAGGCGATCGCAGCGTAGTTTCCCCTTACGACTACTTGGGTCTATTAAATAGGGTGAAAAGCTGAAAATTCGATTGATCCCACTTGAAATTGGAATCACGCAGTCTAATCCATACAAAAAGAAAAAGGGATTTAA |
| CL6483.Contig2_All | PREDICTED: alpha-amylase 3, chloroplastic [*Vitis vinifera*] | ATGGAATTTCAAAACCTCCTTTACATTCACTTTTGCTGAAGCCTCTCTCTCTCTTTTCTCTCTCTTTCCTCCCTCTCTCTCTCTCTCACAAATCCCTTCTTAGCCTTGGTGCTGGTTCATGGATATTGATCTCTTGTGGACTCTTGGGGGGTGGTTTCTAATTTATCCTGATTGCATGGCAGCATTTAGATCAACACCAATCTCTGAATCCTTCACAAAATCACACCCTTTTCTTCTCTTCCTATTTACCCTCCTCCTCATCTTTGTTCTTCTCGTCCACCCAGCGAACCAGATGGCGTCCTCAATTCCAACCACAGCCAGAAAAATTCTTTCATCTGAGCCCTCGGCTACAACTACCACCACAAATGGTTTTCATCCCCAAAAGTTTCACAGTTCTTCAACTAGAAGAGAGTTTGAAGCTGGTGCACATGAAGTTCCTAGCGGCCCAAACCCTATATCAAACAGGTAAACCAACCGGAGCCACTACTTGAAGATCGATGATAAGAGACGTAACGCCTTTAATATTTTATAGTCATCTTTATGGTCGTCATCTCCAGTTGCCATTAATACTGCTTGTCCTCAGACGAGAGGTTTGTTACCTTTGATGGTTATTGTCATCATCCTTAGCAATTTTTTTTTTTTTTTTTTGGTCCTAGAAATGTATAGCAAGAGGCTCTATTAATTTCATATTTGTATGTACCCGTCTATATATATATATATATATATATATATATATATATATATATAATGTGATAATGTAATAATATTACATGATCTTGAATATAACAATATTAAGATGCTTCCCAGACCTTGTAATCTCTTCCCTCGGTAGCCTTAGACCATCTTTGGGCCCCATTCGGTGGTTCATAATGACCCGGCCCGATCTTCATTGCTACTTTTTCATCTATGATGGCCGCGTACACATCCCTTTCTGCCTTGGTAATTTTGACTAAACTCCGACAGTGAATCTTATTCCGGTGTCTAACAGCAATAAGCTCTCCAATTTCAGATCGGTAGTGAGAGAATACGTGATCATAGAAAACTGCAGGAGTTCCTGGATGAGTCAAGATGTAAGCATACCCTTGCATTTCTTTTCCACCTGGAAATCTCCAATGACCCTGAGTAGAACCAGTATCATGATTCTCTACAAAGGTAACAGCACGAGACGGCCACCACCCCACAACCCCAGGAGGTCTTCCTTTCTGATCTGATAATCTCCAATACTCACATCTCTCCAGTGTAGAATGAAGAATCCCTTTTGTTGTAACATCAAATGCACCAGCGGCCCCGTTAGTAGCATTTATCCAGTCAACAATTCTTTGCCTATGTGCATCTTGATTGTGATCCATTTCACCATATGTGTAACTGAGGGAATCCCAATACTCGCCTACAGCAAAATGAGGTTCACTTGCATCCAAGTAGTCCTTGACATAACCTCCCCAAAATCCTCGAACAAAGTCAAGCCTCCATCCATCATAACCAATTTCTTCCCTCAGCCAGCACAACCATTCTTTAAGATCCTTCCTTACAAATTCTTGTGAATGGTCAATGTTTGGAGCAGCATGAAAATTATCTCCACTACTCTTGTTGCCCCTACCCTGAAAATGTGGATCATCCGCAACAACTGCACGGTCATCCCAATTCATGCGACCACCAAAGATATTCCAAACACCATTTTGATTCTGGTATTGAGCACAACGGTGATTCAACACAGCATCTCCAAGAACTTTTATACCAACTTCATGGAATTTTTTTACAAGAACCTTTAGTTCATCAATACTCCCATATCTGGAGTTCAAGTTATATAAATCGTTCGGCATGTAACCTTCAGGTGACACAGACTCTGTAGGTGGTGGTAACCAAACCACACTAAATCCAAGTGAAGATAATTCTGCAGCTTTTTCGTTGAGCTCCAAGTACCATTTTCCAGATTTATGTGATTCCCAGTTGAACCCTTGGCACAGTATTTCATACCCTGTGCCTGTCCCTGAGCTTATCTTTTGAGGCAGTTTTAGTACCTCAGGCTCTAAAACAGCCTCCTCTGAGAAAGTTGGAATGGAGCTTCTGAAGATACTATAGGCTTCTGCAGCCAACTTTTCAATTTCTTGAAGAATAATTTCTTGTGATTCTTTGGTTTTAGTTTTCCGACTCTTCCCCGAGGAAATACCACTCACCAAGTTTCTTATTTCACTGATGATTCCGTCAGTATATGCAGTAGGAGAAACTTCTTGATTTGCTTCTTCAGTCTTTCCAGATATCTGTGCAGAGTCTGCTATCTTTCCTTCAGAAACATCCTTTTCTTCCTTACCTTCAAACTGAACTTGTCTAGATTGAGCAAGCAAGCTACTTGAATTTGAAAGGGGGATGTAGAAATCATTTCCCATACATCTCAACCAAGTGTTCTCATTTAGCTTAAGCACAAAAAGAAATCCCATGAGTTCTTCATCTAAAGTAAACAGCGCCCCTCTTCCATTTCCACTCTCTTTTGGCTGTAACAGAGTCCGCAAAGCCTTTTTCTTAAAAACTTCTGTTTGTGGTGGATGTGGAGCAGATGGAACCTCCCATCGTTTGGCATCATCTTTGCAAACTCCCCAGTGAACAACAACATCTCCAGGTAGATCAGTTTCTAAAAACAGAAGATTCTTAGCTGTCTCGGGACATTTCCTGATAGAGACAACTAATGAGTTTTCAAAGGCAATTTCTTTTACAATAGAATGCTCCTCGTAGAACCCTTTAAGGCGCTTGTTCTTCTGACTAGGATCTGTGGACTGACTACTGCTGTTTTGACCTTTAGAACCAGATCCTTCTGGTTTAAGGAGAATATTAGACAGCTGTCCCAAAGCCCCTGGCCATGTACCCAAGGCCTTGTTTTCCCCTATAGTATTGCTGTCTTCGTGATGACTGTCCACAAGAACAACCTTAAAATCCCTCCCTCTATGCTGATACCAAGCTCCAGTTTCTTCATTCTTTAAAACAAAATTTATAGCTGCAATTGCACTGCTAGTATTAAAATCAATCTTCACTTCATCAAATGTATCATCTTTGGATGGTTTCTTCAAAGGTGTCTCTATCGCATAATCCTTTATGGCAACTGAACCAGGAGGTCTCATTTCAGTAGGAGGTTGATCCCATTCACTGCCAACATCGTCAACATAAGAAACTCCCCAATGGAGTATCCACTTCCCCGGAATACTACATCCAACAGTAAGCTGCCAATTATCTTCACCGTTACCATTATCTAATTTGATACAGATTTTCCCTTCCACCTTTTCGGTTCGTTTGAGTGGAAAAGACTCCTTGAAAACTACGTCTGCAGATTCAGAAGTCTCGACAATAGCCGTATCAGTTGAACTAGATTTGCAAATGTGAACCCTGGGAGGTTTAAAGGTGCAAAAGCTCCCGCCATTGGAGAATAGCTTCGAAGATTTGTTTAGAGAATATGGCTTGACTATCTTCGATGATGGCCGAAATCTGGGATTCTCTCTAA |
| CL10393.Contig2_All | PREDICTED: beta-amylase 7 [*Vitis vinifera*] | ATGCCAAGCAGAGAGCTTGTCATCATTCTCGCTTTCATTCATGTACTTACCATTTATAATGTATAGAGGAATAACTGCAGATCTAGACAACTTAACTGCTTTTTCCCTTCCAATTTCATTTACAGAGGTGTTTCTATAACTTCACTAACAATATACTTTGCTACAAACCCACTCTTGGGGGAAGACATTCATAATACACAACCCATGGAGCCTCTCTTCATTTCCCCACTATACTTGGAGATCATGAACCACTTCTCCATGCATCCTCTTCACGAAGCGTTCAAATTCCAAAAAGTTCAGTGCTTCAGTGAGAAGTGGGGTGAGCCTAAGGTAAGTAAAATTTGAAAAATGCCTCCCATCTGGATCATTAAAAGGCTTGGCACTTTCCAATATTCTGTTATACCCTTCTCTATCATGACAAGGTCGAAAATTCTCACTAACAATTGGTATGCATAGATCCCATGCAGCATTCAGCACTTGCCAGACTAATCCCTCAGGATCAGCCAGTGCATCAAAAAAGCCGTCCTGCTGTTCTAACATGGGCACTTCAGCACATGCAAAGTTTAAAGCAGTCCCGTGCTTCTTTAACATTGTCACAATGGGAGAATAACCATCCCGATTACATGGGTTGTAAAACCCAGCAGTTAATTCAGCAGCATGGCTGGTTGTCTTGTACCACCAGTGAATACCTGGTAGCTTGGCAGCAATGCAGGTTCCTTCAAAAGCTAGCTTGGCTAGAGAAAGCACTCGATCACCATGATCAACTAAAACCTGAGAATACCAATTAAGGAAGAACCTTCCGTAATAGCCATCATAATCACCTCCATCACAGAAAAACCCGGTTTCATATGGCTGGGAATTATAGCAACCCGCATTATCTGGTCCTCTAGCCCAAAATGAGTATCTTCTTGCTTCTGCTGCCTTCCTCAGGTTTTTCAACAGATACTGATCATAACACTGAAATTCACCAATGCCAGGATATCTCCAACCATGCTTGACAGGATTAGATGGGTAACGTAGCTCCCCACATGGACCTAGCCCAACTTCAACCATGGAGATGATACCATCTTCAAAGAAGTCGTTGAATTCTACTCGAAAGCTTCTCATGAAGTCAAAGTAAACCTCAACAGCAGTCCGGCCTCTTAAAACCCGTTCCTTGTCAATCCCCCATGAGAGACATTCAGGGTTGCGTCTTCCCTCTCTATCAGTGAAAAAGATGTCAGGGTTGCTTCGACCAATTTCTGCCACCCAATGGGGCAAAGGAATACAAACATCATCACCAAAGTTGCCCCCACATTCATGAAATGACATTACAACCTGTAACTTAAGCTTAAGATCGCGCACAATCTGAAATAGCTTTTTGTAACCATTCCAGTTGTACTCTTGCGGAGCATGCGCCTCTACTATTCCCCACCAGCAATCTACCATGATGCCATCAACATTAATCGATTTCATTATCCTTAGCTGCTTTAGTAGACTATCTGAATCAACGAGCTCGCACTTTATATTGATGACACCCAATGGTAGCATCAGAAAAATTGGAATGTAAGGAGTGCCAGCAAAATCACGCCCCTGTAACTTCGGGGGCAAGTTAACAACCTGCTTGTCATTAATAGTATTCAATGAGCCACCAATTAGAGACTGATTCTCAGTTTGTTCTCCTCTATCCCCCACCATGGTTGAAGTATGAGAGTGTGCACTTGAAGATAGATCATAAGGTGAAGAACTGGGCATAAAAACACCTTTTATTCGACATGTGTTGTACTCCACTGAGTTTTGATAACCAGGAGAGACACCTCTCAGGGAAACAGGAGTTTGTTGTGATGCGACGTGTGACGACGATGGAGCCACCACTATAGCAGAAACATCATCAAGAGCCCTTGGGACCTGAGATCTTGAAGGAAAGGTGGTTCCATCTGGAAGAACAATCCAGCCAGCTTCCCTCGCCAGAGCCGCAATGACATCATTAATATCAGCTCTAACCCTGAGATTGTAGTTTCCATGCCTTCTAAGGCCTGCCAAGATTCTTGAGGTAATCGCTCTCCGATGCCGCTCTCGTAGCTTTGTTCTCTCTTTCTCTTCTAGAGGCCTAGTCCTCCGAACTCCCCCTTGAGCAACCAATTGTTCTTGAGTTTGGTGGTCATGCTCAAATTGATTGCCGTTGCCTCTCAGTGGAATCCCTCCGTCAACACCTACCAAAGACGGAGTGGTGATGTTCTTCCCATTGTCATCACCATCATCTTCTTCTTTCACATCCATATCCATTTCCTCCTCATCGTCGTCTTCGCTTGTTCCAATCAATTTCTGCATCTCCGCCATAAGCCCCTTTTGTCGTTAGTCTCCCTAACCCTAAATTCCTAACACTTCTAAAAATTTGATCTTTTTGCATCAATTCCAATCAATTTCTGCATCTCTGACACCATAGGCTCCTTTAGCTGCCAATCTCCCTAATCCTAAATTCCTAACACTTTAAAAAATTGGATCTTTTTGTATCAATTCCAATCATTTTCTGCATCTCTGACACCATAAGCCCCTTTAGTTGCCAATCTCCCTAATCCGAAATTTTCAACACTGCAAAAAATTCGACCTTTCTGCATCAAATGAAGCACAATCCTGCACATAGTTAATGCTCTTAGATTTTCCATATGAAAGCCACAATCCCATTTTAAAATTTTGCTTTGCAGGCTGATGCTTGAGTTTCCTTCTTATCAGCTGGACTTCAACTTCTCGCGTAATTCTCGCATGTACCATTTCCTTCTACAAAATCTCAAGCCGCAAAGACCCGTAATTCATCATTTAATCACGAAATTTCACGTTTCTTCGATCAGAGCTCCTCCATCAGACCTTCGCAGAATAGATCTAGTTTTTCTGTTCAAAATCCCAACTGTACCATAGCTAACAAGAATTTCCGGCACGCGGTCGACGTTGTTTCACGGCGACTCGCCGGACTCCGGCGATAGTAATGACAAAGAGAAAGAAAGGCTTAA |
| CL6765.Contig3_All | PREDICTED: beta-amylase 1, chloroplastic-like [*Juglans regia*] | ATGGCATTGAGTATGACGCACCAGATCGGGACCCTAGCCGGAATACCGGTGGAGGCCGGGAATAATTCACACGACTCGTTGGCGACGTTGAGTGCAGCTGCGGTATGGAAGTCGCCGGTGTCGAATATCAGATGCAGGGTGCAGAATCCAGGTACGGAGATCGAATCGATCACGCCGCCGTTAAGTCCGCGCCTAGGTTCCTCGCCGGTGCTCGGCGGTATGAGGCCGGATCTGTCGGTGGCCTGCCGTGCTTTGGTGGCAGATGCTGAGGCTCCGGCGACGGAGGTGGTGAGGGAGTATAAGGAGGGAGGTACGCAGGTTAAGGAGAAAGGGGTGCCGGTTTACGTGATGATGCCGTTGGACAGTGTGACGATGGGGAACACGGTGAATAGGAGGAAGGCGATGAACGCGAGTCTGCAGGCGCTAAAGAGCGCCGGGGTGGAAGGGATTATGATGGACGTGTGGTGGGGGTTAGTGGAGAGAGATTCGCCTGGTGTGTATAATTGGGGCGGGTATGCGGAGCTGTTGGAAATGGCTAAGAAGCACGGCCTCAAGGTTCAGGCAGTCATGTCCTTCCATCAGTGCGGCGGGAACGTCGGAGATTCTTGCACGATCCCTTTACCTAAATGGGTTGGGGAAGAGATGAACAAAGATCCAGATCTTGCATACACTGATCAATGGGGGAGGAGAAATTTTGAATACTTGTCACTCGGCTGTGATAATCTACCAGTCCTCAATGGGCGATCACCCGTCCAGTGTTATGCTGACTTCATGCGTTCTTTCAGAGATAACTTTCAACACCTTCTTGGTGACACCATTGTGGAAGTTCAAGTAGGAATGGGTCCAGCAGGCGAGCTTCGTTACCCTTCATACCCAGAACAAAATGGAACATGGAGATTTCCAGGAATTGGGGCCTTTCAGTGTTATGATAAGTATATGATAAGCAGCTTACAAGCTGCAGCTGAAGCTTCTGGTAAGCCAGAATGGGGTAGCACTGGACCTAGAGATGCCGGTGAGTACAACAACTGGCCAGAAGACACTAGTTTCTTCAAACGGGAAGGTGGTGGTTGGGATCGCGAGTATGGCGAATTCTTCTTAACCTGGTATTCTCAACTCCTTTTAGACCATGGCGAGAGAATACTATCATCAGCCAAGTCCATCTTCGATAACATGGGTGTGAAGATCTCAGTTAAGGTAGCAGGCATTCACTGGCATTACGGGACTCGATCCCACGCCCCTGAGCTCACTGCTGGTTATTACAACACTCGTTACCGTGATGGGTACCTTCCTATTGCCCAGATGCTTGCACGTCATGGTGCCATATTCAACTTCACTTGTATAGAAATGCGTGATCATGAACAACCTCAAGATGCTCTCTGTGCACCTGAGAAACTTGTTAAACAAGTGGCTTTAGCAACCCGGGAAGCACAAGTACCGCTTGCTGGAGAAAATGCTTTACCGCGCTATGATGAGTTTGCACATGAGCAGATCTTGAAGGCAGCGTCATTGAATGTTGGTGGCTCTTCTAACGAAGAGCGAGAGATGTGTGCTTTTACTTACTTGAGGATGAACCCACAATTATTCCAGCCGGATAACTGGAGACGGTTTGTGGGGTTTGTTAAGAAGATGAAGGAAGGGAAGGGTGCAAATAAATGTTGGGAACAGGTTGAACGAGAAGCTGAGCATTTTGTTCATGTAACTCAACCATTAGTGCAAGAGGCTGCTGTGGCTCTTATGCACTAGAAGCTTCAGATTAAGCTTGGTCTTATGCTCGTATAGGGGCTTGGTTACGAAGGATAATATTTGTGGCAAATAA |
| CL6765.Contig4_All | PREDICTED: beta-amylase 1, chloroplastic-like [*Juglans regia*] | ATGGCATTGAGTATGACGCACCAGATCGGGACCCTAGCCGGAATACCGGTGGAGGCCGGGAATAATTCACACGACTCGTTGGCGACGTTGAGTGCAGCTGCGGTATGGAAGTCGCCGGTGTCGAATATCAGATGCAGGGTGCAGAATCCAGGTACGGAGATCGAATCGATCACGCCGCCGTTAAGTCCGCGCCTAGGTTCCTCGCCGGTGCTCGGCGGTATGAGGCCGGATCTGTCGGTGGCCTGCCGTGCTTTGGTGGCAGATGCTGAGGCTCCGGCGACGGAGGTGGTGAGGGAGTATAAGGAGGGAGGTACGCAGGTTAAGGAGAAAGGGGTGCCGGTTTACGTGATGATGCCGTTGGACAGTGTGACGATGGGGAACACGGTGAATAGGAGGAAGGCGATGAACGCGAGTCTGCAGGCGCTAAAGAGCGCCGGGGTGGAAGGGATTATGATGGACGTGTGGTGGGGGTTAGTGGAGAGAGATTCGCCTGGTGTGTATAATTGGGGCGGGTATGCGGAGCTGTTGGAAATGGCTAAGAAGCACGGCCTCAAGGTTCAGGCAGTCATGTCCTTCCATCAGTGCGGCGGGAACGTCGGAGATTCTTGCACGATCCCTTTACCTAAATGGGTTGGGGAAGAGATGAACAAAGATCCAGATCTTGCATACACTGATCAATGGGGGAGGAGAAATTTTGAATACTTGTCACTCGGCTGTGATAATCTACCAGTCCTCAATGGGCGATCACCCGTCCAGTGTTATGCTGACTTCATGCGTTCTTTCAGAGATAACTTTCAACACCTTCTTGGTGACACCATTGTGGAAGTTCAAGTAGGAATGGGTCCAGCAGGCGAGCTTCGTTACCCTTCATACCCAGAACAAAATGGAACATGGAGATTTCCAGGAATTGGGGCCTTTCAGTGTTATGATAAGTATATGATAAGCAGCTTACAAGCTGCAGCTGAAGCTTCTGGTAAGCCAGAATGGGGTAGCACTGGACCTAGAGATGCCGGTGAGTACAACAACTGGCCAGAAGACACTAGTTTCTTCAAACGGGAAGGTGGTGGTTGGGATCGCGAGTATGGCGAATTCTTCTTAACCTGGTATTCTCAACTCCTTTTAGACCATGGCGAGAGAATACTATCATCAGCCAAGTCCATCTTCGATAACATGGGTGTGAAGATCTCAGTTAAGGTAGCAGGCATTCACTGGCATTACGGGACTCGATCCCACGCCCCTGAGCTCACTGCTGGTTATTACAACACTCGTTACCGTGATGGGTACCTTCCTATTGCCCAGATGCTTGCACGTCATGGTGCCATATTCAACTTCACTTGTATAGAAATGCGTGATCATGAACAACCTCAAGATGCTCTCTGTGCACCTGAGAAACTTGTTAAACAAGTGGCTTTAGCAACCCGGGAAGCACAAGTACCGCTTGCTGGAGAAAATGCTTTACCGCGCTATGATGAGTTTGCACATGAGCAGATCTTGAAGGCAGCGTCATTGAATGTTGGTGGCTCTTCTAACGAAGAGCGAGAGATGTGTGCTTTTACTTACTTGAGGATGAACCCACAATTATTCCAGCCGGATAACTGGAGACGGTTTGTGGGGTTTGTTAAGAAGATGAAGGAAGGGAAGGGTGCAAATAAATGTTGGGAACAGGTTGAACGAGAAGCTGAGCATTTTGTTCATGTAACTCAACCATTAGTGCAAGAGGCTGCTGTGGCTCTTATGCACTAGAAGCTTCAGATTAAGCTTGGTCTTATGCTCGTATAGGGGCTTGGTTACGAAGGATAATATTTGTGGCAAATAATGATGTATGGGTATAGCTGCTCTTTTTTCTATAGCTCTGCCTCTGCCTTGTGTTTCTTTTTAATGTGAAATGTTAGATTTTGGTAA |
| CL5177.Contig6_All | PREDICTED: sucrose synthase 2 [*Vitis vinifera*] >CAN68704.1 hypothetical protein VITISV_035889 [*Vitis vinifera*] | ATGTCAAACCCTAAGTTTGGACGAGTACCAAGCATCAGAGAACGAGTGGAAGACACCCTCACTGCTCACCGCAACGAACTCGTTTCTCTTCTCTCCAGGATTTCAATGTTATAGTTTATTTTCTGTGTTTTCTGAGAACCGATCGGATGGTATAGAGCTTTATGCTCCGGGAGGAATCGAAAGAAAAGAAAACCAAATGCGGATGGTTTAGGGTTTTATATATTGTTGATGATGACGTGTTTAATAAATGTTTTGAAGGTTCGTTGAGCAAGGGAAGGGGATTCTGCAACCACATCATCTCATCGACGAACTTGACAAAACTGTCGGAGATGATCACGCTAAGCTGGCGCTTATCGATGGCCCCTTTAGTGAAGTATTAAGATCGGCGCAGGAAGCCATAGTTCTGCCTCCTTTTGTGGCTATAGCAGTCCGTCCAAGACCTGGTGTTTGGGAATATGTTCGCGTTAATGTTTTTGAGCTCAGTGTGGAGCAATTAACTGTTTCAGAATACCTTCATTTCAAAGAAGAACTCGTTGATGGGGAGGCTGATGACAAGTATTTACTTGAGCTTGATTTCGAGCCATTCAATGCCTCATTTCCTCGCCCGAACCGGTCTTCATCCATTGGGAATGGCGTTCAATTTCTTAACCGTCACCTCTCTTCAATTATGTTCCGTAACAAAGACTGTTTGGAGCCATTACTTGAGTTTCTTCGAGCACACAGATATAAAGGGAATGTGATGATGTTGAATGATCGGATACAAAACATATCTCGACTTCAGTCTGCTCTGGCCAAGGCAGAGGATTATCTTTCTAAGCTTCCAGCTGATGCACCCTTTTCAGAGTTTGAACATGCATTCCAAGGCATGGGTTTTGAGAAAGGATGGGGTGATACTGCAACACGGGTATTGGAGATGATTCATCTGCTCTCGGACATCCTTCAGGCTCCTGATCCCTCTACTCTAGAGACATTCCTTGGGAGAGTTCCCATGGTATTTAATGTTGTTATTTTGTCTCCACATGGATTCTTTGGCCAAGCAAATGTTTTAGGCTTGCCTGACACTGGTGGACAGATTGTTTATATACTGGATCAAGTTCGTGCTCTGGAGAATGAAATGCTTCTTAGAATAAAGCGTCAAGGGCTGGATTTTGCACCCAGAATCCTAATTGTGACTAGATTAATACCTGATTCTAAAGGGACTACATGCAACCAACGTCTCGAAAGAGTCAGTGGAACAGAACACACGCATATTTTGCGAGTTCCTTTTAGAACAGAGAATGGGATTCTTCGTAAATGGATCTCAAGGTTTGATGTTTGGCCTTACCTGGAGACTTTTGCTGAGGATGCGGCAAGTGAAATGTCTGCCGAGTTACAGGGTCTTCCAGATTTAATTATTGGCAACTACAGTGACGGGAATCTTGTTGCATCTTTGTTAGCATATAAGATGGGAGTTACACAGTGCACCATTGCGCATGCATTGGAGAAAACAAAATATCCAGATTCTGATATCTATTGGAAGAAGTTTGAGGAAAAGTACCACTTCTCATGTCAATTTACTGCTGACATAATAGCTATGAATCATGCAGATTTTATCATCACCAGTACATATCAAGAGATTGCAGGAACGAAGAATACTATTGGTCAGTATGAGAGCCATACAGCTTTCACTCTTCCAGGGCTCTACAGAGTCGTTCACGGCATTGATGTTTTTGATCCAAAGTTCAATATTGTATCTCCTGGGGCAGATATGTGCATTTACTTTCCGTATTCTGAGACCGAAAAAAGACTAACTTCCCTACATGGTTCAATTGAGAAATTGTTATATAATTCCGAGCAGAATGAGGAGCACGTTGGTACCTTGAGTGAGAAGAAGCCTATAATCTTCTCCATGGCAAGGCTTGATGGGGTGAAAAACATAACTGGGCTGGTTGAGTGCTACGCCAAGAATGCTAAACTGAGGGAACTGGCGAACCTCGTTATTGTAGCTGGTTACAATGATGTTAAGAAATCCAATGATAGAGAAGAAATCTTGGAAATTGAGAAGATGCATTCCCTTATGAAGGAGTACAAGTTGGACGGTCAGTTCCGATGGATATCAAACCAAACAAACCGAGCTCGTAACGGTGAGCTCTATCGTTATATAGCAGACACAAGAGGTGCCTTTGTGCAGCCTGCTTTTTACGAAGCTTTTGGGCTTACAGTAGTTGAGGCCATGACTAGTGGCCTTCCAACTTTTGCAACTTGTCATGGCGGTCCAGCGGAGATTATTGTAGATGGCGTGTCAGGTTTCCATATCGATCCATATCATCCTGATCAGGTGTCTGAACGCATGCTGAATTTCTTTCAACAGTGCAAGGAGGATCCTAGCTACTGGGGTCATATTTCTGATGGTGGACTTCAAAGGATCCTAGAAAGGTATACCTGGAAGATTTATTCCGAAAGGCTAATGACATTGGCTGGGGTCTATGGTTTCTGGAAGTATGTTTCAAAACTGGAGAGGCGCGAGACGCGACGTTATCTTGAGATGTTCTATATACTCAAGTTCCGCAATTTGGCAAAATCCGTTCCTTTGTCAATTGATGAAGACCATCATTAAGATAGAGGTGCTTAATGAATCAGAAGACCCCTTCCAATGGGTTATGGTATTTTGAACTTGATTTAAGTTTGCTTGTTTTGTACATGGACGTGTGTAAAAATCCACGTCATTGTGATTTGTATAATAATCAATGTACATCACTCTTTGAGGGTGCTTTTATGTTGGTTTCGCATTGTAAAAAACCTTGGAATTGTGTGTATCATTACTAGTTTGTGTTTATATTTGGAAGGGGACTAA |
| CL5177.Contig2_All | PREDICTED: sucrose synthase 2 [*Vitis vinifera*] >CAN68704.1 hypothetical protein VITISV_035889 [*Vitis vinifera]* >CBI36885.3 unnamed protein product, partial [*Vitis vinifera*] | ATGATCACGCTAAGCTGGCGCTTATCGATGGCCCCTTTAGTGAAGTATTAAGATCGGCGCAGGAAGCCATAGTTTTGCCTCCTTTTGTGGCTATAGCAGTCCGTCCAAGACCTGGTGTTTGGGAATATGTTCGCGTTAATGTTTTTGAGCTCAGTGTGGAGCAATTAACTGTTTCAGAATACCTTCATTTCAAAGAAGAACTCGTTGATGGGGAGGCTGATGACAAGTATTTACTTGAGCTTGATTTCGAGCCATTCAATGCCTCATTTCCTCGCCCGAACCGGTCTTCATCCATTGGGAATGGCGTTCAATTTCTTAACCGTCACCTCTCTTCAATTATGTTCCGTAACAAAGACTGTTTGGAGCCATTACTTGAGTTTCTTCGAGCACACAGATATAAAGGGAATGTGATGATGTTGAATGATCGGATACAAAACATATCTCGACTTCAGTCTGCTCTGGCCAAGGCAGAGGATTATCTTTCTAAGCTTCCAGCTGATGCACCCTTTTCAGAGTTTGAACATGCATTCCAAGGCATGGGTTTTGAGAAAGGATGGGGTGATACTGCAACACGGGTATTGGAGATGATTCATCTGCTCTCGGACATCCTTCAGGCTCCTGATCCCTCTACTCTAGAGACATTCCTTGGGAGAGTTCCCATGGTATTTAATGTTGTTATTTTGTCTCCACATGGATTCTTTGGCCAAGCAAATGTTTTAGGCTTGCCTGACACTGGTGGACAGATTGTTTATATACTGGATCAAGTTCGTGCTCTGGAGAATGAAATGCTTCTTAGAATAAAAAAGCAAGGACTGGATGTTATCCCTAAAATTCTCATTGTCACTCGACTAATACCCGATGCAGAAGGGACAACTTGCAATCAAAGACTTGAAAGAATTAGTGGGACAGAATACACACACATTCTGAGAGTGCCTTTCAGGACTAAGAATGGTATTCTTCGTAAATGGATCTCAAGATTTGATGTCTGGCCTTATCTGGAAATATTTGCAGAGGATGCATCAAATGAAATTGCTGCTGAGCTGCAGGGAGTCCCAGATTTGATCATAGGCAATTATAGTGATGGAAATCTTGTAGCATCAATATTATCTTTCAAACTGGGAATTACACAGTGCAACATTGCGCATGCATTGGAGAAAACAAAATATCCAGATTCTGATATATATTGGAGAACACACGAGGACAAATATCATTTCTCAAGTCAATTTACAGCTGATTTGATTGCAATGAACAATGCAGATTTTATAATCACCAGTACATACCAAGAGATTGCCGGAAGCAAGAATAATGTTGGACAGTATGAAAGCCACACGGCTTTCACTCTTCCAGGGCTATATCGAGTTGTTCATGGCATTGATGTTTTTGATCCCAAGTTTAACATCGTCTCCCCAGGAGCAGATATGTGCATATACTTTCCATATTCAGAAAAGGAAAGGAGACTTACTGCTCTACACGGTTCAATTGAAAAACTCCTATATGATCCAGAGCAGAATGATGAACACATTGGCATGTTGAATGATCGATCAAAGCCCATCATCTTTTCCATGGCAAGACTTGATAGGGTGAAAAACACGACGGGACTAGTTGAGTGCTATGGTATGAGCGCTAGGCTAAGGGAACTCGTAAATCTTGTTGTGGTTGCTGGTTACATTGACATAAGCAAATCCAGGGATAGAGAAGAAATTGAGGAGATAAAAAAGATGCATAGCCTCATAGAAAAATACAACTTACATGGTCAATTTCGATGGATTCCTGCCCAAATGAACCGTGCTCGTAATGGCGAGTTATATCGCTACATTGCAGACACAAGAGGTGCTTTTGTGCAGCCTGCTTTCTACGAGGCTTTCGGACTAACAGTTGTGGAAGCCATGACGTCCGGCCTTCCTACATTTGCCACTTGTAACGGAGGCCCAGCTGAGATTATTGAGCATGGTATTTCAGGATTTCACGTTGATCCATATCACCCTGAGAAGGTTGCCACAACCATGGTGGACTTTTTTGAACGGTGCCAGAAAGATCCTAGCTACTGGAACAAGATCTCTGATGCAGGGCTCAAGCGAATTTATGAGAGGTACACATGGAAGATATATTCTGAAAGATTACTGACGTTAGCTGGGGTTTATGGCTTTTGGAAGTATGTTTCAAAGCTTGAGAGGCGAGAGACCCGACGGTATCTGGAGATGTTTTACATTCTCAAGTTCCAAGAACTGGTGAAGTCTATTCCACTATCCGTTGATGAGCAACACTGAAGTTTTATCTGCTGGCAAAGTTCTGTATGTCCTTTTTCAATTGAATAAAATATGAATGTGTGAAATATGTAGTGACTTTTTATTGGCAAGTTTCTGAGTTGAATAAACAGCAATGACTAAGCAGCTTGACAAACTGCTACTATTGCTTGTAATAAGTATTAA |
| CL1705.Contig3_All | PREDICTED: neutral/alkaline invertase 3, chloroplastic [*Vitis vinifera*] | ATGGTCTGAGCTTTCCTGAGATTTTTTTTTTTTTTTTTAAATTATTTTTTATGTATATTCTCGAATCAGGTACCTCAAACAAACTGGAGCTCTTGTATTTTCATTGATCTACTTGATTTTAAGTTAACATACGGATTACATTAAATAAAAAATATGTCGATTGGAGCTTCTGAAGCCGTTTTGCAAGTTTTGTCTGGCTCTGTACCTCGCTTTTTTGATTCTGATTCATGTTTTAGCAAATCAGATTCAATATTTCTTTCTAAATCCTACATGAAATGCGTAAAGAAAAGGGCCTCAAGGTATATGAAATTGCTCAGCTGTTCGAGTACACGACAAAATCCCATAGGGACATATCGTTTTCATAGTATAGGTGGTGGTTTGTATGGAAATTTCATCATTGACCGATTTCAGTTATTGAGTTGCAAATGCCAACGAGCTGAAAGTGTAAGTGAGGTAAATGTGGAGGACGGGAATGGAACTTGGTTCGTGGATGAAGCAAAGAAAATAAGTCCAATTAATGGCATGGTGAATACCACAAATGTTCTAGACTTAAAGGAGGTTCAAAAATTGGATCAGGAGGATTTCTCATCCAATGGCAACATACCCACAGCTGAAACAGTCAGAGATACCTTTCACAAGACTAGTGTTGATTCTATTGAGGATGAAGCATGGGATCTACTGAGGGAGTCTATGGTTTATTATTGTGGCGGTCCTATTGGAACAATTGCTGCTAAGGACCCAACCAGTTCCAGTGTTTTGAATTATGATCAGGTCTTCATCCGTGACTTTATACCTTCTGGTATAGCTTTCTTATTAAAGGGAGAGTATGATATTGTCCGGAACTTCATCCTTCATACACTTCAGTTGCAGAGCTGGGAGAAAACAATGGACTGTCATAGTCCTGGACAAGGGTTGATGCCTGCTAGTTTCAAGGTGCGAACGGTTCCTTTGGACGGTGATGATTCTGCAACGGAAGAAGTATTGGATCCTGACTTTGGAGAGGCAGCAATTGGCCGTGTTGCACCAGTTGACTCTGGATTATGGTGGATTATATTATTACGTGCATATGGAAAATCCTCTGGGGATCTTTCAGTTCAGGAGAGAGTTGATGTCCAAACTGGAATCAGGATGATTCTAAGGCTATGTCTGGCAGATGGTTTTGATATGTTCCCAACTTTATTGGTGACAGATGGTTCTTGCATGGTAGATCGCCGCATGGGGATTCATGGCCACCCCCTGGAAATACAGGCGCTATTTTATTCAGCGTTACTTTGTGCGCGCGAGATGCTTGCACCTGAGGACGGATCAGCTGATCTTATCCGAGCGCTGAACAATCGATTGGTTGCTTTGTCTTTCCATATTAGGGAGTATTATTGGATTGATATGAAAAAGCTGAATGAGATTTACCGTTACAAGACGGAAGAATACTCATATGACGCAGTTAACAAGTTCAACATTTACCCAGATCAGATTTCTCCTTGGCTGGTGGAATGGATGCCCAATAAAGGAGGCTATTTAATTGGAAATCTGCAACCAGCTCACATGGATTTTCGTTTCTTTTCCCTAGGAAACTTGTGGTCTGTTGTAAGCAGTCTTGCAACGTTGGATCAGTCGCATGCCATATTGGATCTAGTTGAAGCTAAATGGGCAGATTTGGTGGCTGACATGCCATTCAAGATATGTTACCCAGCTCTTGAAGGTCAAGAATGGCAGATCATCACAGGCAGTGATCCCAAGAACACGCCTTGGTCCTACCACAATGGAGGTTCCTGGCCTACTTTGCTCTGGCAGCTCACAGTTGCGTGCATTAAGATGAATAGACCAGAAATTGCTGCAAAAGCTGTTAAGATTGCAGAGAAACGCATATCACGAGACAAGTGGCCGGAATACTATGATACCAAAAGAGCCAGGTTCATTGGGAAGCAGTCACGGCTGTTTCAGACTTGGTCAGTTGCCGGATACCTTGTATCAAAGCTCCTCCTTGCTGACCCAAGCGCAGCCAAAATTCTCATGACTGAAGAGGATTCTGAGCTTGTTAATGCCTTCTCTTGCATGATCAGTGCCAACCCAAGAAGAAAACGTGGCCGCAAGTCTTCCAAACAGACTTATATAGTATGACTAGTTTCTGTCGGTCTTGCAAGTTGCAACTACTCGATTCCATAATTATTTATTCATACGTTATTTGAATATGGGTAATTATCTGAATATGGGCCATTCTAGTGTAGTCTGATTTTATTTGATTTTGTGTCACAAACTATTCATTGAAGTATACACTTTTGTAAAGTTATTTAA |
| CL5669.Contig4_All | probable alkaline/neutral invertase B [*Quercus suber*] | ATGAAATACTCAACTCTAAGTCACATCCTCTCCAAATCATAATAAACCAGGTTAAGACTAAAATGGGGGAAAGATAAAACCCACCATTAAAAAGTCCATGAAGCTGATCTCTTCATGACAGGCTTCATCATCTGTTTATCTTCTTCAAGTGATATCATACCCAAATGAGATGGATCTTCCAGCATCATCTTAGCCACCAAATAACCCGCAATCGACCACGTTTGTGATTTACGAGCCTGCTTCCCAATATATCGACCAAGCTTCCCATCATAATATTCAGGCCAGCTGTCTTTCAACAACCTAGTTTCAGCAAGTTCAATGGCACGTCTAGCAATTTGGGGTCGTCCAGTCTTGATACACGCAGCCGTAAGAAGCCATAAAAGCACTGGCCAAGATCCTCCATTATGATAACTCCACCTAGTATTTTTTGGGTCACATCCTGTCACAATCCGCCATTCATGGCTTTCAATAGCTGGATAACAGACCTTAAGCGGCATTTCGCCGACCAACTCTTCCCATCGCGATTCTATAAGATCCATAATTGCGGTGGATTGTTCAGGGGTTGCTAAAGATGACAAGATTGCAATACAATTACCCAATGCAAACCAGCGAAAGTCCATTCTTGCAGGACTGACATTTCCAATAAAGTAGCCACCGTGAGTTGGCATGAAATCAAAAACCCATTCTGGGAGAGAATCGGGAATCACGTTGAATTTATTAACTGCAGTGTGAGAGTACTCCTCAGTTTTGTATCTGTATATATCGTTTAGCTGTTTCAAGTCTAACCAGAAATAGCTTCTCATGTGATAACCCAAGGCATGGAGACGTTTAACTATCCGTTCCACAAAGTCCTTTCCTTCAACATCTTGTTTTAACAAAACCAAAGCACATCTTAAAGCCATGAAGAAAAGGGCCTGTATCTCAATGGGATACCCATAAACACCCATCCTGCGATCAATCATACAGCATCCATCAGCACAAAGGAGAGTCGGGAATGTGTCAAACCCTTCTGAAAGACACAAACTAAGAATAAGGCGCATACCCTTCTGACATTCAGGCAATTCAGCCAGGGTAGAGTCTCCTGTAGACTTTGTGTATGCCCGAAGCAATATAATCCACCAAAATCCAGAATCAACAGGTGCGACTCTTCCTATTGCACTCTCGCCAAAATCGGCTATCAAAGTCTCAGTGTTCCTGATTGGATCATGTAATACTTTGAAACTAGCTGGCATTACTCCTTCTCCTAGCTGGAACCGGTCAATCTTTTTCTCCCAAGATTGAAGACGAAGTGTCTTCAAAATAAAATTTCTAACTATTTCTGGCTCCCCATTCATCAAAAAAGCCAATGCACTCGGAACGAAGTCTCTTACAAAAACCTGATCATAGTTAAGTTCTTCTTCAGAACTATCTAATGCTGCAATTGTCCCAACCGGTTGACCACGGAAGTATACGAGTGACCGCCTCAAGGATTCCCAAGCTTCACAAACCATTGGATGAGGCTCAAATCCCATCTGTGACCTTGGGGTATTAAAGCCAGACCTTCTACCTGGTGAAAATATATTCTCTAAATGGTCCATTATTCGGGACGAATATTCGGCAGTTCTGGAAGGGGGAAGAGGGGACAATCCCATAGAAAATTCGTTGAGCGACCTTTCATCGCATGATCTCTGTCTTTCCATATTCAGAGGCCTTGGCCTGTCCAATAGCCTTGAGAAATCACATTCCTCAAGATCAGCCATAGCACTTGATGTATCAGTACCTTTTATACTTCCATTCTGACACACATCCACAGAGAGTACAGACATTTCCAAAGTCTCAAATGTAGAAAAATACCAACTTATTGATGATTTTCAACACACTGCATTTTAACTAACATAAATTAGCTTTCCAGAAAAATATAAGTAATAAGGAAAACATTCCTATGCACCAAGCAGTGCACAAATCCAATCAGGCAATTGAGCACCGTATATCTTAA |
| CL5669.Contig5_All | probable alkaline/neutral invertase B [*Quercus suber*] | ATGAAATACTCAACTCTAAGTCACATCCTCTCCAAATCATAATAAACCAGGTTAAGACTAAAATGGGGGAAAGATAAAACCCACCATTAAAAAGTCCATGAAGCTGATCTCTTCATGACAGGCTTCATCATCTGTTTATCTTCTTCAAGTGATATCATACCCAAATGAGATGGATCTTCCAGCATCATCTTAGCCACCAAATAACCCGCAATCGACCACGTTTGTGATTTACGAGCCTGCTTCCCAATATATCGACCAAGCTTCCCATCATAATATTCAGGCCAGCTGTCTTTCAACAACCTAGTTTCAGCAAGTTCAATGGCACGTCTAGCAATTTGGGGTCGTCCAGTCTTGATACACGCAGCCGTAAGAAGCCATAAAAGCACTGGCCAAGATCCTCCATTATGATAACTCCACCTAGTATTTTTTGGGTCACATCCTGTCACAATCCGCCATTCATGGCTTTCAATAGCTGGATAACAGACCTTAAGCGGCATTTCGCCGACCAACTCTTCCCATCGCGATTCTATAAGATCCATAATTGCGGTGGATTGTTCAGGGGTTGCTAAAGATGACAAGATTGCAATACAATTACCCAATGCAAACCAGCGAAAGTCCATTCTTGCAGGACTGACATTTCCAATAAAGTAGCCACCGTGAGTTGGCATGAAATCAAAAACCCATTCTGGGAGAGAATCGGGAATCACGTTGAATTTATTAACTGCAGTGTGAGAGTACTCCTCAGTTTTGTATCTGTATATATCGTTTAGCTGTTTCAAGTCTAACCAGAAATAGCTTCTCATGTGATAACCCAAGGCATGGAGACGTTTAACTATCCGTTCCACAAAGTCCTTTCCTTCAACATCTTGTTTTAACAAAACCAAAGCACATCTTAAAGCCATGAAGAAAAGGGCCTGTATCTCAATGGGATACCCATAAACACCCATCCTGCGATCAATCATACAGCATCCATCAGCACAAAGGAGAGTCGGGAATGTGTCAAACCCTTCTGAAAGACACAAACTAAGAATAAGGCGCATACCCTTCTGACATTCAGGCAATTCAGCCAGGGTAGAGTCTCCTGTAGACTTTGTGTATGCCCGAAGCAATATAATCCACCAAAATCCAGAATCAACAGGAGCGACTCTTCCTATTGCACTCTCGCCAAAATCGGCTATCAAAGTCTCAGTGTTCCTGATTGGATCATGTAATACTTTGAAACTAGCTGGCATTACTCCTTCTCCTAGCTGGAACCGGTCAATCTTTTTCTCCCAAGATTGAAGACGAAGTGTCTTCAAAATAAAATTTCTAACTATTTCTGGCTCCCCATTCATCAAAAAAGCCAATGCACTCGGAACGAAGTCTCTTACAAAAACCTGATCATAGTTAAGTTCTTCTTCAGAACTATCTAATGCTGCAATTGTCCCAACCGGTTGACCACGGAAGTATACGAGTGACCGCCTCAAGGATTCCCAAGCTTCACAAACCATTGGATGAGGCTCAAATCCCATCTGTGACCTTGGGGTATTAAAGCCAGACCTTCTACCTGGTGAAAATATATTCTCTAAATGGTCCATTATTCGGGACGAATATTCGGCAGTTCTGGAAGGGGGAAGAGGGGACAATCCCATAGAAAATTCGTTGAGCGACCTTTCATCGCATGATCTCTGTCTTTCCATATTCAGAGGCCTTGGCCTGTCCAATAGCCTTGAGAAATCACATTCCTCAAGATCAGCCATAGCACTTGATGTATCAGTACCTTTTATACTTCCATTCTGACACACATCCACAGAGAGTACAGACATTTCCAAAGTCTCAAATGTAGAAAAATACCAACTTATTGATGATTTTCAACACACACAAATTCAGCAGCAAATTCTCAACGAACCAACTCAGAAATGAAAGAAAAATGAATCACAGCAGAAATAGTGTGAATTTAAACGAGCATTTGGTGAAACAATCAGGTGGGTCACGCCAGCAATCGACCAAACCTCCGTATTGTGTATGATTGGTAGAGTGTGACGGGCAGGTGGGTCGCCTTCAAAATGGCCTACCCGTCTGTATCTATATGTATGTATTCGCACCAATCAAAGCTGGAATGCACGAATCACGATGAGTTTCAGTACACCTCGAGTCTCTGTGACGTTTAA |
| Unigene13486_All | PREDICTED: putative invertase inhibitor isoform X1 [*Pyrus x bretschneideri*] | ATGCCACATATGCGCCTCCAAATCTCCGACCTTTAATTACAACTTCTGTTCAACTTCTCTTCAATCGATTCCCATTAGCCACGCCGCGAACCTCCAAGGACTTGCGGTTGTTGCAATGGAGCTAGCACTAGAAAATGCCACCAGTACAATCTCAGCCATTAAGAAGATGTTAGACGGTGGAGTTTTCGATCCGTTTGCGTTGAAATGCTTAGATGATTGCTTGGGACTGTACTCGGATGCTGTCAACACATTAATAAACTCAGTTGGAGCCTTTTTGAGTCAGCAGTATGGTACTGCTAATGTCGGGGTGAGTTCGGTCATGGAAGCGACCAACACTTGTGAGGGAGGGTTTGTGGAGAAGGAAGGTGAAGTGTCTCCAATGACAAAGGAGAATAATAGTCTCTTTGAGTTGTGTGGTATTGCGTTGTGTATTTTTCACTTGCTTACTTTAAACCCTGCTTTCTGATAGGCTGCTTCAGTGCATTTGGTTGAGTTGGTCCCTTTAATTCGATTGCTTTTGAATTGATGGTTCTGTAAATATTGGCTGAGCTTCTTGATCATTTTGGAAGTCTACCATCGCCATTCTATCCACTGACTTCTTGATATTCCCCAATAGGTGTTTCAATCGGCTCTGGACTCTGGTAGTCTTTGAGGGTCTCAGTTTGCTCTTGTTTAATAGAGGACAGACATGTTGTATTTGCCACTGGTACATTGTGCTTGTATTGAGTTCCAAATATTTAAATTTTAGACGTAGTTAAGGACTACAATTTCAGCCTTTCAGAATACGAAGTTTTTTGTCAAAGTTTGTAA |
| Unigene12859_All | Hexokinase_1 domain-containing protein/Hexokinase_2 domain-containing protein [*Cephalotus follicularis*] | ATGATGGCGTGATTTAATAATCACATATATATCTATTAACATATAGATATAGAGAGAGATGGGGAAAGTGGGGTTGGGAGTGGCGGTGAGTTGCGCTGTGGCGACGTGCGCGATCGCAGCAATACTGGTGGGGAGGAGAGTGAGGAGTAGGCGCAAGTGGAGCAGGGTGGTGGGGGTGTTGAGAGAACTGGAGGAGGCGTGTGAGACTTCGGCGGGGAGGCTGAGACAGGTGGTGGATGCCATGGCTGTGGAGATGCACGCTGGTTTGGCTTCTGAAGGTGGTTCTAAGCTCAAAATGCTCCTTACTTTTGTTGATAGCCTTCCTAATGGGAGTGAGAAGGGAACTTATTATGCACTACATCTTGGAGGTACTAATTTTAGGGTTTTGCGGGTTCAGCTAGGAGGTCAAAGATCTGCAATCATGGCGCATGATGTGGAACGGCAACCCATTCCCCGTCATTTGATGACAAGTACGAGCAAGGATCTCTTTGATTTTATTGCTTCTTCACTAAAGCAGTTCGTTGAAAGTGAAGAGAAAGATTCTGAGTTTTCAGCAGTCAAAAGAAGGGACCTTGGATTTACATTCTCTTTTCCCATGAAACAAACATCTGTTTCATCCGGCATTCTAATTAAATGGACGAAAGGGTTTAGTATCCAAGACATGGTTGGAAGCGATATTTCTGATTGTTTACAGCAAGCAATGACTAGAAGAGGACTAGACATGCGGGTAGCAGCACTGGTAAATGATACTGTGGGAACATTAGCTCTCGGACATTATCATGATGTGGACACTGTTGCTGCAGTGATAATTGGAACAGGCACAAATGCCTGCTATTTGGAGCGGACAGATGCCATTATTAAATGTCAAGGCCTTCTTACGACTTCTGGAGGCATGGTTGTCAACATGGAATGGGGGAATTTCTGGTCATCTCATTTGCCAAGAACTTCTTATGATATAGATCTGGATGCCGACAGCCCCAATCCAAATGATCAGGGTTTTGAGAAAATGATATCAGGAATGTATCTAGGTGACATTGTAAGGAGAGTGATTCTCAGGATGTCACAAGAGTTGGATGTATTTGGGCCTGTCTCTTCCAAGTTATCGGAGCGCTTCATTCTGACGACACCATTAATGGCTGCAATGCACGAGGACGAATCGCCCGAGTTAAAAGAAGTTGCAAGAATCTTCAATGATATCCTAGAGATCCCTGACGTGCCTTTAAAAGTCCGAAGACTCATTATTAAGGTGTGTGACGTTGTGGCTCGAAGGGCCGCTCGTTTGGCAGCTGCGGGTATAGTTGGTATTTTGAAAAAGATTGGGCGGGATGGAAGTGGAGGTATCACAGGTGGAAGAATGAAAGGGAGTGATAGTAAGATGAGAAGAACGGTGGTTGCCATTGAAGGAGGTTTGTATACAAGCTATTCATTGTTCAGGGAGTACTTAAATGAAGCTGTGACTGAAATTCTAGGGGATGATATTGCCCCCCATGTCATTCTTAAAGTCACCGAAGATGGGTCTGGTACTGGAGCAGCTCTCCTTGCTGCCTCTCATTCTTCCCTTACTGTAGATAGCTAGGTTCCAGTATACATAATTATATAATTATATATATAGCCCCCTGTAAATGTAGATTTCATTTTACATTTTTTATATTTCATATATATATTTATATAAAGGTTTCTGGTGTTTGTACAGTAACCGCAGCCTTAAGATCCCATTGTATTTTGTGCGATAGTGGCTGTCTATAGGGTCTCCAGTTCAAGCTGTATTTCTCCCTATAA |
| Unigene23911_All | Hexokinase-2 like [*Actinidia chinensis* var. chinensis] | ATGTCGTTGTCTCTGTTTCCACCTCGACGCCAAAGCTCGTTCTTCTTCATTGACTCCCCCTTTTTCTTCCTGGTTCTTACTGTCTTTCGACAATGTCGGTTACCGCAAGCCCACCCACCGTCGGATCCTTTGGTCATTATCGGTCTTATAGACGGACGGCTCGGATCAAAATGTGCATCCGGTCTAACACCATCTCCGTGGCTCCAATTCTGACTAAGTTACAGAAGGAGTGTGCAACTCCTTTGCCAATTTTGCGCCACGTGGCAGACGCCATGACTGATGATATGCGAGCTGGCCTTGCTGTTGATGGTGGTAGTGATCTCAAGATGATACTCAGCTATGTTAATAGCCTTCCAAGTGGGAATGAGAAGGGCTTGTTTTATGCTTTGGATCTTGGCGGCACAAACTTCCGGGTGTTGAGAGTGCAATTAGGTGGCAAGGATGACCGTGTTATTGCCACTGAATTTGATCAAGTATCAATCCCTCAAGAGCTTATGTTTGGTACCACTGAGGAACTTTTCGATTTTATTGCTTCTGGGCTGGCAAAATTTGAACAAGAGGAGGGTGGAAAATTCCACCTCCTGCCTGGAAGGAAAAGGGAGATTGGATTTACATTTTCTTTCCCAGTGAAGCAGACGTCCATTGATTCTGGCATACTAATGAAGTGGACAAAGGGATTTGCAGTCTCTGGAACGGCAGGAAAGGATGTAGTAGCATGTTTGAATGAAGCTATGGAAAGACAGCAACTAGATATGCGGGTGTCTGCCCTGGTTAATGATACTGTGGGAACATTAGCTGGAGCCAGGTACTGGGATGACGATGTCATAGTTGCTGTCATTTTGGGTACTGGAACCAATGCATGCTATGTAGAACGCATGGATGCTATTCCCAAACTACAAGGCCAGAAGTCTACTTCTGGAAGAATGATTATTAACACTGAGTGGGGAGCATTCTCAAATGGCCTTCCTTTAACTGAGTTTGATAGGGATATGGATGCTGCGAGTGTCAATCCTGGTGAGCAGATATTTGAGAAGACAATCTCTGGTATGTACCTTGGTGAAATCGTAAGACGAGTACTACAAAAGTTGGCTGAAGCAGATGCTTTGTTTGACGAATATGTCACAGAGAAACTATCCACACCTTTTATACTCAGGACCCCAGATATATGTGCAATGCAGCAGGATGACTCAGACAATCTAGAGGCTGTTGGATCAATCCTATATGATGTAGCTGGGGTAAAGTCAAACTTAAGTGCAAGGAAGATTGTTTTAGAAGTTTGTGACACTATTGTGAAGCGAGGAGGGCGCCTGGCTGGTGCTGGAATTGTGGGGATTCTCCAAAAGATGGAGGAGGATTCAAAAGGTCTCATCTTTGGAAAGAGGACAGTGGTGGCTATGGATGGAGGTCTTTATGAGCACTACCCCCAGTACAGAAAATACCTCCAAGATGCGGTCACGGAACTTCTAGGGTTAGAAATATCAAGGAATGTAGTTATAGAACATTCAAAAGACGGGTCTGGGATCGGTGCTGCTCTCTTGGCAGCTTCAAACTCAAAATATGAACACGATTTCTAGAGCAGGACATTAA |
| Unigene24242_All | hexokinase-1-like [*Hevea brasiliensis*] | ATGGAAAAGGAAAATACTATCCAAACAAAATTAATACAAAGAGAGAGTATTATGACTATTATCTACAAATCTTAAGTCGATCTTCATTTCTTATCTTCCGAGAAGATAGCTCGGATTTGTTGATTTTCCATAAAATTTCATTCAGTCTACGGTTTTTCTGACCTTTATATTATGAATCTTTGAACCCCCAAACGACAACTTACTCACTACCAAATTCCAATCCATATTTTATCCATTTCAATCAATCTCAATTCCGTGGATTGCTTCGATCTGTATCTTGTGTAATTTTTTTGTCCATTCAGTTCATTTTTAGGATTTTTAGTTGGTGGTGATGGGCAAAGTGGCTGTAACAGCGGCGGTGGTATGTGGGGCTACAGTGTGCGCTGCGGCGGCGCTGATCGTGCGCCACCGTATGCAGAGCTCGGGGAAGTGGGCTAAGGCCATGGCAATACTGGACGACTTTGAGGACGAGTGTGCAACTCCGATTGGGAAGTTAAGACAGGTTGCCGACGCCATGACTGTTGAGATGCATGCAGGTCTCGCATCCGAAGGTGGAAGCAAACTCAAGATGCTCATCAGCTACGTAGATAATCTCCCCACCGGGGATGAAGACGGATTATTCTATGCGTTGGACCTTGGTGGCACAAATTTTCGTGTGCTGCGTGTACTATTGGGTGGGAAAGATAAACATGTTATCAAACAAGAATTTGATGAAGTTTCAATTCCACCACATTTGATGGTTGGAAATTCAGCTGGATTATTTGATTTCATTGCTGAAGCACTTAAAAAATTTGTTGCTACAGAAGGTGAAGGTCTTCATCCTTTCGCAGGTAGACAACGAGAGCTGGGTTTTACTTTCTCATTTCCAGTTAAGCAATCATCAATTGCATCCGGGTCTCTTATTAAATGGACGAAAGGCTTCTCTATAGAAGACGCGGTTGGAGAGGATGTGGTCGGGGAATTGACAAAAGCCATGGAAAGAGTTGGTGTTGATTTGCGCGTGGCAGCATTGGTCAATGATACAATTGGAACATTAGCGGGAGGTAGATACAACAATCCGGATGTTGTTGCTGCTGTGATCTTAGGTACAGGAACAAATGCAGCTTATGTAGAGCGGGCGCAAGCAATTCCCAAGTGGCATGGTGTTCTACCTAAATCAGGAGAGATGGTTATCAACATGGAGTGGGGTAACTTCCGGTCATCTCACCTTCCACTGACAGAATACGACCACGAACTGGATTTAGAGAGTTTAAACCCCGGGGAGCAGATTTTTGAGAAAATTATTTCCGGCATGTACCTAGGAGACATCGTACGCAGAGTCTTGCTCAAAATGGCCGAGGAAGCCGAATTTTTTGGCGATACTGTTCCTCCAAAATTGAGAATCCCATTCATATTAAGAACCCCGGACATGTCTGCTATGCATCAAGACACGTCTTCTGATCTAAAAGTAGTTGGGAGCAAACTAAAGGATATCTTAGAGATACCTAATACTTCTTTGAAAACGAGGAAGATTATTGTGAAGCTTTGCGACATTGTTGCTGTACGTGGGGCTCGACTATCTGCTGCGGGGATTTTGGGTATTCTCAAGAAATTGGGAAGAGACGTGACGAGGGAAGGGGATAGCGAGAAGCAGAAGTCGGTGATTGCATTGGACGGCGGATTGTTTGAGCACTACACAAAGTTCAGCAAGTCAATGGAGAGCACTCTGAAGGAGTTGTTGGGTGAGGAAGTGTCTGAACACATTGTTATTGAGCAATCCAATGATGGTTCGGGTATTGGAGCAGCTCTCCTTGCGGCTTCTCACTCTCAGTATCTTGAGTGTTGACAATGGTCAGAAGATTTCAGTTTTCCTTTGTTTGTATATTTACTTTCCCCCTTCCCTTCATAATTTACTATCGTGTTTTAGAGGAAGGGCCAGCATATCCTTGGTTTATGGTATTGCAGAGTGTGACTGGGCAGTGGGTGATGAGTTAGGTGGTTTGCTTGGCATTGTGCCCCCTTGGGTGGGGGGGAAGTGTATTGGTATTAGTATCAAAGTTTCTCCTGCTGTATTAGGGAATAAAATGGGGTTGTAATTTTTGTTTTCTTTAGTTAAATTTCTAGAGGCCTTTTTTTGTGCCCTCTATTGCAATAA |
| CL2999.Contig5_All | PREDICTED: phosphoglucomutase, chloroplastic-like [*Gossypium hirsutum*] | ATGGCGCTTTCATACGCCCAGTTCCACCAACAATGCAGAATAATTTCTGGTTGCCAAACGTACCCCTTAAGTGCCTTGAACAAGTGTCATAGAGATGTAGTGGAGAATCACAAATATTAAACCTTCAATAAAAGATACTGATTTCCAAGTATTCTTATTTCCCATAGAGTAATAACAGAAATCAGAAACTACATACGATTATTCTCTTATTTTCTACTTATCAATATTTCTTATATTTTACACACTCTGACTAGTTGTTCGATAAAGCCAGATTTTACCAAAACTAAAATGAACAGAAAACAAAAAAAAAAAAAACAAACAAAAACAAAAAAGAAAGATATATATGCATCGCTAGAAAAAAGAAAACAACGGGCAATTCCAACCTGGGCATGTGCTTTATGTGATGACTGTAGGTTTCTCCCTTCCTGTGAAGTCCTTCAGCTTCGATACAGACAACGCCAAATCTATCAATGGTTTTAGTGCTATTTGGGCATCCACGTCGTGTTTAGATGCATCCGGCTCAAATTGTTCAATGTAAATTCTTACTGTTGCACCTGCCGAACCGGTTCCCGATAAACGAAATATGATCCTTGATCCATCTGTAAATACAAACCGAACTCCTTGCTTTGATGCCACACTTCCATCCACAGGATCAGTGTATGTAAAGTCATCCGCAAATTGAAGAATGTAACTTCCGTAATTAACGCCAGCCTTGCTCTTTGAAACCAAATCTCTCAGATATTCCAGCATTTTATTGGCTCCTTCAGATTCACATTCTTCATAGTCATATCTAGAAAAGAAATTCCTTCCATAAGTTGCCCAATGCTCCTTCACAACATCAGAGGCAGAAAACAATTTCTCCCCTGGCTTTTTGTCCTTGTTCCGATATGCGATAATTGAAAGCCATGCTAACACAGCCCATATGCCATCCTTTTCCCGGATGTGATCAGAACCTGTTCCAAAACTTTCTTCCCCGCAAATCGACAATTTCCCAGCATCCATAAGATTCCCAAAAAATTTCCATCCAGTGGGGACCTCAAAGAAAGGAAGATTCAATTTATCAGCAACACGATCGAGAGCACCACTTGTTGGCATAGATCGAGCTAAACCCTTAGAGCCACTCTGAAAATATGGGATGGCTTTTTGTGCATTAGCTGCAATAATTGCAACAGAGTCTGAAGGAGTAACAAAAAAACCTTTTCCCAAAATCATGTTTCTATCACCATCTCCGTCACTTGCAGCTCCAAAATCAGGTCCATTCTCAGCATACATAACGTCGACTAAATCCTTAGCATATGTAAGATTGGGATCTGGATGGCCATGCCCAAAATCTTCCAGAGGCACTCCATTTGAAATAGAATCCAGAGTAGCTCCTAGCTTCTCGACAAATATAGGTTTTGCGTAAGCACCTGTAACTGCATGCATGGCATCAAATATAAACCTGAAATCTGGTCGTGAAAGAAGACTTTTGACAAGCTGGAAATCAAATACATTCTCCATAAGTTCCAAATAGTCAGAAACTGGGTCAACTACTTCAACACTGAAGTTCCAATATTTTGAAATTCCAAGACGAGAAAGGTCGACATCTGGAATGTCTGCCATCTTAATTTCAGAGATAGAAACGGTGTTCCCATAGATCTTGTCGGTAATGGATTCAGGTGCAGGTTGTCCACTGTTGTAATTGAACTTGATACCCCAATCATAATCAGGCCCGCCGGGATTATGGCTTGCACTCATTATAAAACCACCGTTGGCCTTCCTCTTCCGAATTACAGCAGAAACGGCAGGTGTTGACAAAATACCTTCCTTGCCAACCAAAATCTTGCCAACGCCATTGCCAGCAGCGATTTTGATAATTATCTGTGCAGCTTCTTTGTTGAAATATCGACCATCACCTCCTAACACCAACACCCCGTCCTTAAAATCCTCAGGCGGTAATGAATTAAACAACGCCTGGATCCAGTTCGCAAGGTAATTCTCTTGCATAAAAACTTTAACCTTCTTCCGGAGACCGCTCGTTCCGGTCTTCTGGCCTTCGATCGGCTTAGTCAGAACCGACTTAATCTTAATGCCTTCGGGTTCGATGAAGGTGATGGAAGGAGAAGCAGAAGAAGAAGCTTTAATGTTGTGTGAAGGCGAAGTTCCAAAAGCGAGTTTTGCAGGAAATAGGAGAGGTGAGTGCGAGAAATGTCTGTTTTGATTAGAGAACGTAGCATTTGTTCTTGCTAAAGATGAAAACGGTGTGAGCTTGAAGACATTCTCCAATCTTACTGAGAACGCCATGGATAA |
| Unigene22708_All | UDP-N-acetylglucosamine pyrophosphorylase [*Actinidia chinensis* var. chinensis] | ATGACTGGGAGGTTCGTAAATCGACCCCATATCCTCGTAATTATTGCGGATCACAGATATCTGAGGCTTCGTCTAGTAGTCATGTGTCTGAATACATGGCTATGAAATATCCTGAGCTTGTGGGTATACAACCGGAGCGTAAACTGGGTAATGGAAAGAAAGATGTAGGAGCTTCACTGGATTGGTTTATGTCACCTCCTAAAACTTGTGTTTTAATGGAACCACCTGAAGAAAAGTCATTGAACCAGTTGCCTAGTACTCCTTGTGTCTTCAATCAGCAAACAAATCTGAAATCTTTTAATCAAGGAGTTTTTACTCCACTGTGGAAAGAAGCTGAAAGCACGATTCAAGGGGGGAGACGACCTGGGGAGAATACATTAAAGAAAGAGTTGTGGACAAGATTTGAAGCTGCATCAACTAATGAGGTCTCTTTTAATGTTTCTCTACTCAAAGAGACGAATCAGAGAGGGTTTTTGGCTAGATTGGACGAGGTTTCTCGCAATGAAACTAGTAAAAATGATTTGAGATAAGAAATATTATGTGCCTAAAGTTGAGCAGTTTTTGAGTTTGTTAA |
| CL7879.Contig2_All | Retrovirus-related Pol polyprotein from transposon TNT 1-94 [*Cajanus cajan*] | ATGTTTTCTTCATGATTTGTCATTAATTTGGCATTGAGGCAAGCTTGTAATTCCTTGTTTATTAGTGGATTGGACTCATCGTCCCGTGGAGTAGGAATTTTCCGAACCACGTTAAATTCTTGGTGTTCTTGTTCTTTACTTTAATTATTATTTGCTATCAACAATTCTGCTAAACGTAGGGCTGTGTTAATTAGATTGCCGTGACAAAACCAGTGCTAATTGTGGTATTGTTAGGGGTTTGATAATTCTCTACAATTGGTATCAGAGCCCAGGTTACCTATAGGCGTTGATTGGTGAATAAGGATGGCGGAGTCGAATAGAAAATCGTCAAAGGTACATGGATTTGATATTGTCAAATTCGATGGAAAGAACAACTTCACAAATTGGCAGACGGAAGTAAAAGATATTTTGATTTCCTTAAAGCAATTTAAGGCTTTGAAGGGAAAACCATCGACACTTCCAGAGGATTGGACAGATGAGGAATGGGAAGAGTTTGATTTAGAGGCTTGCAGTACAATCAGACTATGTTTAACGCGTGAGATTATGCACAATTTTGCAAGCGAGACGTCGGCTAAAGAGCTTTGGGATAAATTACAGTTGATGTACATGAAGAAAGATTTGTGCACGCATCTGGCGTTAAGGCAGAAACTCTACACGTTCTCTTGGGTTGCTGGAAGATCTTTATCCGACCACATTAATGCTTTCACAAAGATTACATGTGCTATGGAGGACATAGGGATAAAGTTGGATGAATGTGAGAAAGCGATGATTTTGCTGTGTAGGTTACCTCAACAGTATGAGGCAACAATTGACAATCTGTTAACCGGTAAAGATGAAGAGACACTGAAGTACGATGAGGTTGTAACAGCTTTACAAGCTAAAGCAATCAGGGATACTGAGACAACGTCTAGTAGTGAGGCGCAGAGTCTTTTCGTGGAGAGAGGTCGACAGACTAACAAAAGGGGAAAGAACCAGAATAGGAGTCGATCAAAGTCTAGACCGAGGAAAAATAAGGAAGAAGAGGAATGCCATTGGTGCCATCAGCTGGGACATTACAAGTTTGAATGTCCAAAGTATCTCAAGAAAAAGGAGAAAAATAAGAAAAACGACAAGGAAGAAGCTTCGGTAGCTCAGGTTGGGGATGAGGTGCTGGAATACTTGCTGGGTGGAGATGACTCAGAAGTTTTGACAGCAGAGACATCTGAGGATGTCATGTATGAGTGGATATTTGATACTGGTGCATCCTACCATATGTGTCCCGCACGGGAAAACTTCTCTACTTATAGGGAGGTGGATGGAGGTAAGGTTTTGATGGGTGACAAAGGTTCTCTTAAGACCGTCGGTGTAGGTTCTATCAAGTTTCAGATGTCTGACTCAGCAGAACGGACTCTTACAGAGGTTCGACATGTACCTGGCTTGAGGAAGGGGCTGATTTCTTTGGGCGTTCTAGAGGCAGCTGGTTATGGATTCTCAGGTAAGGACGGAATTCTCACAGTCACGTGGGAGGGCAGAATTATCATGAGAGGTGAGAGGTATGGCAATCTCTACTTCCTACGAGGGAAGCCACTTCTTGGTGTTATGCAGACATTGGTTTCACAGGTTGATGAGAAACGGTATCTACAGACTCAGTTGTGGCATATGCGTCTTGGACATATGAGTGAGAAAGGGTTGACAGTATTGAGTAAGCAAGGCTTACTAGGAGATGACAAGCTTTGTAAGCTTGATTTTTGTGAGCATTGTGTGTTTGGCAAGCAGTGCAGAACAAAGTTTGGGAATGCAGAGCATCGCACTAAGGGGATTTTGGATTATATCCATTCAGATGTTTGGGGGCCTTCTAGAGTTACATCTCAAGGAGGAAAAAGGTGGTTTGTTACCTTCATTGATGACTTCTCCAGGAGAGTATGGGTTTACACTATGAGGCAAAAGAGTGAAGTCAGAGAAATTTTCATCAAGTGGAAAGCACTTGTTGAAAAGCAGACAGGCCGTGCAGTCAAGAGGATTCGATCAGATAATGGTGGAGAATACACAGAAGATCCTTTGAAGGAGTTTTGTGAGCAGCATGGCATTGCCAGACATTTTACAGTGAAGGGTACACCACAACAGAATGGTGTGGCAGAGCGGATGAACCGCACCTTATTGGAGAAAGCACGGTGCATGAGGTCCAATGCTGGACTTAGTAAATTTTGGTGGGCTGAGGCGGTTAACACGGCGTGCTATCTCATTAACAGATCACCATCTACAGCAATTGGGTTGAAAACCCCAATGGAGGTATGGACTGGAGGTATGGACTGGTACACCTACTACTTATGATAATCTTCGTATTTTTGGATGTCCTGCATATTATCACATTACAGAGGACAAGATAGGAAAGAGAGCTATGAAGTCTATCTTCTTAGGCTATCCTGAAGGAGTTAAGGGTTATCGCTTGTGGTGCCTTACGACTAAGAAGATTGTTGTGAGTAGAGATGTGAAATTTGATGAAACAGTCATGGCAAATGTTCAGAGATTCACAGGTTGGGACTCGCCGACAGAGAACAGTGAATTGAGTAGAGATGTGGATGATGATCAGGTGGAGTATATTGCTCCTAGGACTTCTAGACCAATTCGGGATCCAGAGTCAGTTTCAGATTCAGATATGGGCCAGACATCACCTTCACAGGGTGAGATATCGACAGATTTAGAGGATAACAGAGCAGACTTGGATACTGAGGGTACAGAAGCAGAGACAGAGGTTCCTACAGAGGAGGAGCCTATTCAGCAACATGATGAGGAGGATCAGCAAGACGGCCTATCCTTGGCAGAAAGGCGAGGAAGAAGACAGAATAGAAGAGCACCTGTTCGGTTTGGTTATGAAGAGTATGCCTCTTGTGCTTTCATAGCTTATGATGAGGAGGAGCCTACTACCTACAGAGCAGCTTTGAGGAGTTCTCACAGTGCAGAGTGGAAGCAGGCTATGAGTGAAGAGATACAATCTCTACACAAGAATAAGACTTGGGAGCTAGTCAAAGTTCCTAAGGGAAAGAAGCCGATTGGTTGCAAATGGGTCTTCGTGGTAAAGGATGGGATTCCAGATTCCAAGGGTATCGTTGATGGTGTACGGTACAAAGCAAGATTGGTAGCTAAAGGGTATGCACAGAAAGAAGGTGTGGATTATAATGAAATTTTCTCTCCAGTGGTTAAGCACACTTCTATCCGGATCCTGTTAGCATTGGTGGCGCAGTATGACATGGAGCTCGAGCAAATGGATGTCAAGACTGCATTCTTGCATGGTGATTTGGAGGAGGACATTTACATGTCACAGCCCGATGGTTTTATTGTTGCTGGGAAGGAAGACCATGTGTGCAGATTGAAGAAGTCGTTGTATGGGTTAAAGCAATCGCCTAGGCAGTGGTATCTTCGGTTTGACAGGTTCATGGTGAAGCAAAGGTACAAGCGCAGTGAATATGATCATTGTGTGTACTATAAGAAGTTGAACAGTGGATCTTACATCTATCTCTTACTTTACGTTGATGATATGCTCATTGCTGCCAAGAGCAAATATGATATAGACTTGCTGAAGGATCAGTTAAGTAAGGAGTTTGAGATGAAAGATTTGGGAGCTGCGAAGAAGATCTTAGGCATGGTGATCAGTCGTGACAGGAAGCTGGGCAGGTTGACAGTCTCTCAGAGGGACTATGTGGAGAAGGTTCTTAGTCGGTTTGGGATGTCAGATTCTAA |
| CL8635.Contig2_All | PREDICTED: sucrose synthase [*Nelumbo nucifera*] | ATGTAGTAGCTGGACTACAAAACATGAAGTAGGTGGACGAAATTCACAAATTGTAACTCGAATCGATGGGCTTTCATGCCCTAGTACAAACAATGCCAATAAGAATATCCTTAAATGTCATGTTTTCCCTCGGCGTTCCAAAACAATGACAGTTATTCCACACAACTCAGAAACCCTCCACTTGCAACAACACAAGTCTTTCGTCTACTCAACAGCAAGAGGAACTGATTGAGCCAGCTTACGGTACTTGAGGGCATAAAACATTTCAAGATAGCGGCGAGTCTCACGACGATCAAGGTTGGAAACATACTTCCAGAAGCCATACACACCAGAGAGTGTCAACAACCTCTGAGAGTAAATCTGCCATGTATACTTCTCATTGATTCTCTGCAGGCCTGCCAATGAAATATTGTCCCAATGTGTTGGGTCTTCCTTACATTTCTCAAAGAAGTCGGCAAGAAGCACAGCTGCCTGATCACCGTGGTAGGGATCAATATGGAAACCAGATTTTCCATGCACTATTATCTCAGCTGGGCCACCATGACAAGTTGCAAATGTTGGCAAGCCACAGGTCATGGCCTCAACAACAGTCAATCCAAAAGCCTCATAGAAAGCAGGTTGCACAAAAGCCCCCTTTGTGTCTGCAATGTAACGATAAAGTTCACCATTCCTCACGCGGTTCATCTGGGAAGAAATCCATCTGAACTGGCCATTCAACTTATAGGTCTCTATGAGCCCATGCATCTTCTTCATCTCTGCTTGCTCTTCCAAATCTTTGGATTCCTTCCTTCTATCACCGGCAACCACAACAAGGTTAACCAACTCACGAAGCCTCTTGTTCTTACCATACCACTCAACTAGTCCCGTCATGTTCTTCACATTGTCCAACCTTGCCATGGAGAATAGGATTGGCTTGCTTCGGTCTTTCAAAACACATAAGTGCTCTTTATTCTCAACAGAGCTGTAAAGAAGCTCTTCGATTTCTGGGTGGAAGGACTTCAGTCTCCTTTCCTCCTCTGTATAGGGGTAGTAGATGCTCATATCTGCTCCTGGTGAGACAATGTTGAACTTGGGGTCGAACACGTCAATCCCGTGGACAACACGATAGAGCCCGGGGAGAGTGAAAGCTGTGTGACTCTCATACTGCCCAACAGTGTCCTTACTTCCAGCAATCTCTTGGTAGGTGCTGGTAATGATAAAATCAGTATGGTTCATGGCTATAAGATCAGCTGTAAACTGGCATGAGAAGTGATACTTCTCATCAGATTTTTTCCAGTAGATGTCAGAATCTGGATATTTAGTTTTCTCCAGAGCATGCGCTATAGTGCACTGAGTTACTCCAAACTTGTGTGCTAACAAAGAAGCAACAATGTTTCCATCACTGTAATTGCCAATGATCAGGTCTGGCTTTGCCCGCAACTCTCCAGCAACTTCACTTGCAACATCCTCAGTAAACTTTTCAAGGTATGGCCACACTTCAAATCGTGAGATCCATTTACGAAGGATACCCTTTTCTGTTCTAAAGGGAACTCGAAGAATATGTGAATGTTCTGTACCAAAAACTTTTTCAAGACGCTGATTGCAAGTAGTCCCTACTGCATCAGGGAGCAGTCGAGTAATAATGAGAATTCGAGGAATGATATCTAATCCTTGTTGCTTTATACGAAGAAGCATCTCGCTCTCCAAGGCACGGACTTGGTCCAAAATATAAACAACCTGGCCACCAGTGTCAGGATAACCCAAAACATTGTCTTGGGCAAAATATTCATGAGGAGAAAGGATAACAACATTGAAAACCATTGGAATTCTCCCAAGGAATTTCTCAAGACTGCTTGAATCAGGAGCCTCAAGAAGGTCCAAAAGCAGTGAAACACTCACTGTTCTCCCAAACTTGATCCAAGAGTTTATGATGGAGTTACAAGTCGTCCCTGTTGCATCAGGGAGCAGTCGTGTCACAATGATGATTTTTGGAATGATATCCAGTCCTTGCTGCTTTATACGGAGAAGCATTTCACTCTCCATGGCACGAACTTGATCCAAAATATAAACAACCTGGCCACCAGTGTCAGGATAACCCAAAACATTGTCTTGGGCAAAATATTCATGAGGAGAAAGGATAACAACATTGAAAACCATTGGAATTCTCCCAAGGAATTTCTCAAGACTGCTTGAATCAGGAGCCTCAAGAAGGTCCAAAAGCAGTGAAACACTCACTGTTCTCCCAAACTTGATCCAAGAGTTTATGATGGAGTTACAAGTCGTCCCTGTTGCATCAGGGAGCAGTCGTGTCACAATGATGATTTTTGGAATGATATCCAGTCCTTGCTGCTTTATACGGAGAAGCATTTCACTCTCCATGGCACGAACTTGATCCAAAATATAAACAACCTGGCCACCAGTGTCAGGATAACCCAAAACATTGTCTTGGGCAAAATATTCATGAGGAGAAAGGATAACAACATTGAAAACCATTGGAATTCTCCCAAGGAATTTCTCAAGACTGCTTGAATCAGGAGCCTCAAGAAGGTCAAGGAGTAAATGAATCATTTCCAACACACGTCCGGCGGTATCGCCCCACCCTCGTTCCAAACCAAACTCTTGGAAATTGTGTTCAAATTCGGAATACAGAGTATCCGGAGTGAGGTCGGTCAGACATTCATCCGCCTTCCTTAAAACATGCTGGAGGGTGTTCAGGTTTTGTATTCTGTCATTCAACATCATTGTCTTTCCATTGTAGTGGTGAGCACGGAGAAATTCAAGCAGTGGGTGAAGGCTCTCCTTGTCATGGAACAGTTTAGCAGAGAGATGACGATTGAGGAACTCCACACCATTTCCAATGGACTTGGAAAGAGTTGGGCGAGGAAAGGATGCATTGAATGGTTCAAAGTCCAACTCCAGCACAAAATTGCCATTGGTGCTTCCATCTACAAGTTCTTCCTTGAAGTGCAAATACTCACTCGCAGTCAGCTCATCAACAACAAGGGCATTCACATTCACACGTATGTATTCCCAGACACCAGGCCTTGGACGAACAGCAAGTGCAACCCATGGAGGCAAAACTATTGCTTCCTGTGTTGATTTCCAAACATCTCCAAATGCACCATCAGTTAACTTCTGTCTTTCCACTTCAGGTATTTCTTCAATAGCAGATATAAGATGATGGCGCTGTAAAATTGCTTTTCCTTGGCTTTCAACCCTTGTCAGGAAGGCCAAGATCTCATTTCGATGAGTAATCAGAGTTTCATCCACACGCTCCCGGAGGCTGTGAACGCGAGTAAGAAACCTTTCAGCCATGAATGACTTCTTTGTGATCTCTTAATTTTCAGAAACCTTCTTTTGCAAAGGAACAGAGAATGAAACAGAGGAACTAA |
| CL10451.Contig1_All | PREDICTED: cellulose synthase A catalytic subunit 2 [UDP-forming] [*Vitis vinifera*] | ATGCATATGTATATTTCTTTCTCTCTCTAGAATTCTTCCTTTCTTTGTTCTGTTTAATCGGTGCTGCTTGAGAGAAGAGTACATACAGAGCCCAGAAGAATCTGTGAGTTTTCCAGTGACGAGTGAGGCAAATAGGCCAACTTGGCGAGGTCTTGATTTGTGTAATCTGGGGTGTGGATTTGAGGCAGGTTTGTTTTGATGGACACCAAGGGAAGACTCATTGCTGGTTCTCACAACAGGAATGAGTTTGTTCTCATCAATGCTGACGAGATTGGTCGAGTGACATCTGTAAAAGAACTGAGTGGGCAGATTTGCCAGATCTGTGGGGATGATATTGAGATTACAGTGGATGGAGAACCATTTGTTGCCTGCAACGAATGTGCATTCCCCGTGTGCAGACCTTGCTATGAGTATGAAAGAAGAGAGGGCAATCAGGCTTGCCCTCAATGCAAAACCAGATACAAGCGCTTAAGAGGGAGTCCCAGGGTTGAAGGTGATGACGAAGAGGATGATATTGATGATCTTGAGAATGAGTTTGATTTGGCGAGCAATAACAGGAGAGATCCCCGCCATATTAACGAGGCAATGCTTTCTACCCGCTTCAGTTCTCAAGTCAATGCTTTTGGGGTCTTTACGCCGTCTGAGCTGGATTCCTCTTCCGTTGCTCCTGATATCCCCCTCCTGACATATGGGCAAGAGGATGTTGGGATTTCTTCTGATCAACATGCTCTTATTGTTCCCCCATATATGGGTCGTGATAAACGAATCCATCCAATGCCATTTCCTGAATCTTCCATGTCATATGTTTCAGTGCAACCCAGACCCATGGACCCTAAAAAAGATTTAGCAGTATATGGCTATGGGAGTGTTGCATGGAAGGAAAGAATGGAGGATTGGAAGAAAAAGCAGAATGATAAGCTTCAGGTTGTTAAGCATCTAGGAGGAGGTGGTGGTGGGAATGATAATGGAGATGAACAGTATGATCCCGATTTACCCAAGATGGATGAAGGCAGGCAACCTCTTTCAAGGAAGTTACCAATTCCTTCAAGCAAAATAAACCCATACAGAATGATAATTATACTCCGAATTGTGATTCTTGGCTTATTCTTTCACTATAGAATTCTACACCCAGTTAATGATGCATATGGACTGTGGCTGACATCAGTTATATGTGAAATATGGTTCGCTGTATCTTGGATATTCGATCAGTTTCCAAAGTGGCAACCAATTGAGCGAGAAACATATCTTGATCGGCTATCACTGAGGTATGAGAAAGAAGGGAAGGCATCCGAGTTAGCGGCTGTAGACATTTTTGTAAGCACAGTTGACCCTACAAAAGAACCTCCACTAATCACTGCGAACACGGTGCTGTCCATCCTTGCCGTAGATTATCCGGTGGACAAAGTTGCATGCTATGTCTCAGATGATGGAGCTGCCATGCTTACTTTCGAAGCACTCTCTGAGACATCTGAGTTTGCAAGGAAGTGGGTCCCATTTTGCAAGAAATTCAACATTGAGCCCCGGGCACCGGAATGGTATTTTGCTCAGAAGGTTGACTATCTGAAAGACAAAGTACATCCAGCATTCGTCGGGGAACGCCGTGCAATGAAGAGGGAATATGAAGAATTCAAAGTTAGGATAAATGGCTTGGTTTCCACGGCACAAAAGGTTCCTGAGGAGGGTTGGACAATGCAGGACGGGACTCCATGGCCTGGCAATGATGTCAGGGATCATCCTGGAATGATCCAGGTTTTCCTTGGCCATAATGGTGTTCGTGATGTTGAGGAAAACGAGTTACCCTGTCTGGTTTATGTTTCTCGTGAGAAAAGACCAGGATTTGATCATCACAAGAAAGCTGGAGCTATGAATGCTCTGATGCGGGTTTCAGCAATCATCTCAAATGCCCCGTACTTACTGAATGTTGATTGTGATCACTATATAAACAACAGTAAAGCACTTCGCGAAGCCATGTGCTTCATGATGGACCCAACGATAGGAAAGAAAATATGTTACGTGCAGTTTCCTCAAAGATTTGATGGGATCGATCGTCATGATAGATACTCGAACCGCAATGTTGTTTTCTTTGATATCAACATGAAAGGACTAGATGGGATCCAAGGACCAATTTATGTCGGAACAGGGTGTGTGTTTAATAGGCACGCTCTCTATGGTTATGATGCTCCCATCAAGAAGAAACCCCCACAGAGAACATGTAATTGTTGGCCAAAATGGTTCTCCTGCTGTTGTGGATCTAGAAAGAAGAATATGAAGGGTAAGACAACTGAGAAAAAGAGGAAGATCAAGAACAGAGAGGCTTCAAAGCAGATACACGCACTTGAAAACATTGAAGAAGGAATTGAAGGAATAGATACTGAAAAATCATCTCTCATGCCCCAAGTAAAATTTGAGAAAAAATTTGGACAGTCACCAGTTTTCATCTCTTCAACACTACTAGAAGAAGGTGGGATTCCAGAAGGAACATCTTCTGCATCACTTTTGAAAGAAGCTATCCATGTCATTAGCTGTGGGTATGAAGATAAAAGTGAATGGGGGAAAGAGGTGGGATGGATATATGGCTCTGTTACAGAGGATATACTAACAGGCTTCAAGATGCACTGTCACGGCTGGCGCTCAGTGTATTGCATACCCAAACGGCCTGCATTTAAGGGATCAGCTCCGATAAACCTCTCCGATCGTCTACACCAGGTTCTGCGGTGGGCCCTTGGATCTGTTGAGATTTTATTAAGCAGGCATTGCCCAATATGGTATGGCTACGGATGTGGTTTGAAAAGTTTGGAGCGTTTTTCGTACATAAACTCAGTAGTTTATCCCTTGACATCAATTCCCTTGATCGCGTACTGTACCCTGCCGGCTGTCTGTCTGCTTACAGGAAAGTTCATCGTCCCTGAGATTAGCAACTATGCCAGTATTGTGTTCATGGCCCTCTTTTTATCCATAGCTGCAACGGGTATCCTTGAGATGCAGTGGGGAGGTGTTGGCATCGATGACTGGTGGAGAAATGAACAGTTCTGGGTAATTGGTGGCGTCTCAGCACACCTATTTGCTCTCTTCCAGGGTTTGCTCAAGGTTTTGGCAGGAGTCAATACAAACTTCACTGTTACATCTAAAGGAGGAGATGATGGAGGGTTCTCTGAGCTCTACCTCTTTAAGTGGACATCTTTGTTAATCCCTCCCATGACCCTGCTGGTCATAAACATAATAGGGGTCATGGTTGGGGTTTCAAATGCTATCAATAACGGCTACGAGACATGGGGACCATTGTTTGGTAGGCTTTTCTTTGCCCTGTGGGTGATTGTCCATCTTTACCCTTTCCTTAAGGGATGGATGGGGAAACAAGAAAAGCTTCCCACCATTATTGTTGTCTGGTCAATTCTTCTAGCCTCAATCTTTTCACTTCTATGGGTCCGAGTCAACCCATTTGTGTCAAGAGGCGGTATCGTATTGGAAGTTTGTGGGTTGGACTGCGACTAAATTTGCACAAAATTAGAAGTTTTAGATGGTTCCAAGTTATTTGTAGGATTTTGTTTGGGATTTGCAGAGGGGTGTTTGTTTTTGTCCAATATGAAGACATCCCTCTACACGGTAGTACTTATTATGTGCTGTGAAGGAAATTTGTTGTAGGGTGAGTAGATACAAAGGACTGGGACTGGGAATTTTACACAATTTTTTTCATTTCTTTTCTCTTATTGTTTTGTGTTAATCGATTCTTTTGATGCATGTGATTTTTTGGGTTAGAGGGGATTGAATTTAGAGGTTGTGTCTGTAATCCCTAGGGAACATAAGCTTAGTTGTTTATACTTTAAATCCCTTCCCATAAAAGTAA |
| CL12349.Contig2_All | Cellulose synthase [*Macleaya cordata*] | ATGCGTGAAAGATAGTTTGGAGGTGGATCTAACTGAGAACTATGCTGTAGATCTCAGTGAAATTGAATTTGCAGATTTGATCCGAAGAAGCTATGGAGTCAGAAACAGATGCGAAGTCAAAACCTTTTCAACTCTCGGCTGCCCGCGTATGCCAGATTTGTAGTGATGATGTTGGCGCAAATATAGATGGTGAACCCTTCACTGCTTGTAATGTTTGCTCCTTTCCTGTTTGCCGAGCTTGTTATGACTATGAGATGAAAGATGGTAACCAGTCTTGTCCTCAATGCAAGACCAAATACAAGAGGCATAAAGGAGGTCTTCTCGTTGTGGGTAACAAAGTTGGAGATGCCAATGCTGATGATGTTGCCAATGACCAGAAGCAAAAGATTGCAGAGCGCGTTCTGAGCTGGCATATGAGCCATGGAAGAGGGGACGATGTTGGACCTCCAAACTATGATAAAGAGATTTCTCGTAATCATATTCCTCTGCACACTAATGGACACACGGTTTCTGGAGAGTTCTCGGCTGCATCTCCCGAGCATCTTTCAATGGCATCTCCTGAAAGTGGCGGTGGAGCTAAGCATATGAATCCAGTCCCTTATTCGACATACATCAATCAATCACCTAATATTAGGGGGGTAGAATCCGCAAGGGAATTTGGGTCATCGGGGTTTGGCAATGTAGCATGGAAAGAAAGAGTTGATGGTTGGAAGATGAAGCAGGAGAAGAATATGGCTCCACCTAGTGTTGGTCACGCACCTTCTCATGTGGCTCCAATGAGTGTTGGCCATGCACCTTCTGAAGGCAGGGGTGGTGGTGATATTGATGCTAGCACTGATGTGGGGGCTATGGATGATGCTTTACTAAATGATGAAGCCCGCCAGCCTCTTTCAAGGAAGGTTTCTATACCTTCCTCTAGAATTAACCCTTATAGGATGGTCATAGTTCTGCGGCTTGTTATTCTTTGCATTTTCTTGCACTACCGTTTAACAAATCCAGTTCCCAATGCTTTCGCTTTGTGGTTAGTATCTGTGATTTGTGAGATATGGTTTGCATTTTCATGGATATTGGATCAGTTTCCAAAGTGGTTACCTATAAACCGTGAAACTTATCTTGACAGACTGTCTTTGAGGTATGACCGAGAAGGAGAGCCATCTCAGCTGGCTGCTGTTGACATTTTCGTTAGCACGGTCGACCCTCTCAAAGAGCCTCCCCTTGTCACAGCCAATACTGTCCTATCTATTCTTGCAGTCGACTATCCAGTTGATAAAGTCTCGTGCTATGTTTCTGATGATGGAGCTGCTATGTTGTCATTCGAAGCTCTGTCCGAGACATCAGAATTTGCAAGAAAATGGGTTCCTTTTTGCAAGAAATACAGTATTGAACCACGAGCTCCAGAATGGTACTTTGTTCAGAAAGTTGACTACTTAAAAGATAAGGTTCAACCATCATTTGTCAAAGATCGCAGAGCAATGAAGAGAGAATATGAAGAGTTCAAAATTCGTATCAATGGTCTTGTTGCGAAGGCGCTAAAGGTTCCCGAGGAAGGATGGGTCATGCAAGATGGAACACCTTGGCCTGGAAATAACACCAGAGATCATCCAGGAATGATACAGGTTTTCTTAGGCCACAGTGGAGGAGTTGATAGTGCCGGTAACGAGCTTCCAAGATTAGTTTACGTGTCTCGTGAGAAGCGTCCCGGTTTTCAACATCACAAGAAGGCCGGTGCTATGAATTCACTTGTTCGTGTATCAGCAGTCCTTACCAATGGGCCCTACATGTTGAATCTTGATTGTGATCATTACATAAATAACAGCAAGGCGTTGCGGGAGGCTATGTGTTTTCTAATGGACCCAAACCTCGGAAAATCAGTTTGTTATGTTCAGTTTCCACAGAGATTTGATGGGATTGATAGGAATGATCGATATGCTAACCGCAACACCGTTTTCTTTGATATAAATCTTAGAGGGTTGGATGGCATCCAAGGTCCTGTCTACGTGGGTACAGGATGTGTTTTCAACAGAACAGCCTTGTATGGCTACGAGCCTCCTCTCAAAACTAAGAATAAGAGATCAGGTTTTCTTTCCACATGCTTTGGCGGATCAAGAAAGAAGAGTTCTAAATCAACTAAAAAGGATTCAAATAAGAAGAAATCAGGAAAGAACATGGACCCTACTGTGCCAGTATTCAATCTGGATGATATAGAAGAAGGATTTGAAGGTGCTGGATTTGACGATGAGAAGTCACTGCTCATGTCACAGATGAGTCTGGAGAAAAGGTTTGGTCAATCAGCTGTCTTTGTTGCTTCAACACTAATGGAATATGGCGGTGTTCCACAGTCTGCTACTCCCGAGATCCTTCTTAAAGAAGCTATCCATGTTATTAGTTGTGGGTATGAGGATAAGTCGGATTGGGGAAGAGAAATAGGATGGATCTATGGGTCTGTTACAGAAGATATTCTCACAGGATTCAAGATGCATGCCCGTGGATGGCGATCAATCTATTGCATGCCAAAACTCCCAGCCTTTAAAGGATCTGCTCCCATTAATCTTTCTGATCGTTTGAATCAAGTGCTTCGGTGGGCTTTAGGTTCAGTTGAAATCCTTCTCAGTCGGCATTGTCCCATATGGTATGGTTATGGTGGAAGGCTCAAATGGCTTGAGAGGTTTGCTTATATCAATACAACCATTTACCCAATCACAGCTCTTCCCCTTCTTGCATATTGTACATTGCCAGCTGTTTGTTTGCTCACTGGAAAGTTCATAATTCCACAGATTAGTAACATTGCCAGTATCTGGTTTATCTCCCTCTTTCTTTCCATCTTTGCAACGGGTATACTGGAAATGAGATGGAGTGGTGTTGGGATAGACGAGTGGTGGAGGAATGAACAGTTTTGGGTTATTGGAGGTGTTTCTGCTCACTTGTTCGCCGTCTTTCAAGGCCTTCTCAAAGTCCTTGCCGGAATTGACACCAATTTCACCGTCACTTCCAAAGCATCCGACGAAGGGGATTTTGCCGAGCTTTATATGTTTAAATGGACAACTCTACTCATCCCCCCTACCACATTACTCATAATCAATCTGGTGGGAGTTGTTGCCGGAATCTCGTATGCCATTAACAGTGGGTACCAGTCTTGGGGTCCTCTGTTTGGGAAGCTGTTTTTTGCTTTTTGGGTGATTGTTCATCTATACCCCTTCCTTAAGGGTCTCATGGGACGTCAAAACCGAACGCCAACAATTGTTGTTGTGTGGTCGGTTCTTCTGGCCTCTATCTTCTCCTTGTTATGGGTTCGCATAGATCCCTTTACAACCAAGGTGACGGGTCCAGATGTTGCAATTTGTGGAATCAATTGCTAGAGGATGAAGAAAAAGAATAGAATCACGGGCAAAAGCATAAACGATTTTTCTTTCAGTATTTTTTTCCGACACACTTCAAGTTAGTGTGCTAATTATTATGTAGGTATATTATTCATTGTTTTAATTATTTGATTTTTTTTTGAAGTCTTTACACTTTGGTAAAAGTTAAATGAAAGAAACTTGAGTTTTTTTTTTTTTTAATTGTATCTTTCAATCTTTGTATAATAA |
| CL143.Contig15_All | LOW QUALITY PROTEIN: protein CELLULOSE SYNTHASE INTERACTIVE 1 [*Carica papaya*] | ATGAATCCTGCTCTGAAACATAGCCTTGATGAATCCTGCGCTGTTCATATAGGCAGGCAACCCTAGTGTGATAGAATTGGTAAGTCCTAGCCCACCGGACTAAGACAGAGAATTGGCAAGTCCTAGCCGCGCACGCTGCTGTTTCTTTACCATCCAAGGTTTATTGTATTATAATCCTAGTTTCACTGGGTTCTTTATTAAAAAGCCATCTTCCACCGCCTTTAGTTCACTAGTCATTCCACAAACGGTTTCTTTATAATTATGTTATTAACGCAAGGATCCGTTTTGTGTTACTATAATACCCTTTTTGTGTTTGCTTGGGTTTTCATTCTCAGTCGTCCCAACAACTAAACCTCTGGAAGACGGGTCTCTCGAATTCAAAGAAAGCGCAGCGAAGCGAAATTGAAGATTCTTGTCGAGTCCTAAAATCAGAGTTGAAGACATATCTCTCGATCATTACAGGTTGCAGATGGATGCTCAGGCTAGTATTGAAGGAGAAGCGGTATTGAGCTTAGTTCGACATGTTCGAATATTGGCACGAGAATGTGAGTCCAGTTACAAGGATGGAGTAACAGAGGGATTTGGTTTTGTCAAGGAAATTATTCGATCCTGTGACCAAGTTCTTGAGATGGACATGACTGGATCCGACATTGCTAGAAGTGAGCAGCCCGAAAATGTCATCCCTACAGAAGAAAAAGCCATCGCTACCGAAGTGAGTTCTGTCATAGATAATCTTCGATCAACAGAAGAAGGGAAGAGAAAGCAAGGGGCTATTGGTCTGCGATCTATTTATAAATCACATGTACGAATAGAAAAAGTGTTGCTTGAGGATTGCATTCGACCATTGATCGATCTTCTCAATTCTGAGTCGGAAGGAGTTCAAGATGAGGCAGTAACAACCATCCTTGATATTTCAAAAGGTGGTGATGTGAGGGACACCCATTGGACTGCAGAATGCAAAATCTTTTTAACCAAACTTGTGCCTGCCCTCTGGAAGAAGCTTGAACGTGCAAGCTCATTTGACAAGCCCCTAATTCTTGCATTGAGAAATATCTGCACTTGCTGCGAAACATATTATTATGGCCCTCGTTTTCTGGTTAAAACTACGGAAGCTGGTGGGTTGGATATACTTTTGAAGTTGGAAAGTACAGAAGATACATCATTTCTTATAGGTTATATATGTGATGAGCTAGTTTTTTTTTTTAAATCGTGGCATTCAACTTTCTAAAAAGAAAGAATCTGCAGACACTCTACTTTATTATTCTGTTCGGCATAAAGAAGTACGGAAGGTACTAGTTAAATCTAAATATATGGATTTTATAATAGAAGCTGCTGGCGAGCACGGGCAAGAGTATGAAGCACTGAGGGAAAATGCAATGGCCTTGCTCATAAATATTCATGGTGGTTTATGGGAGGCGATATTAAGCATTTGTGGAAGACTGACTTTAAAGACATTACCGGCGTTGGCTTGTGCTGTTGCAAGATATGATAGCAAAACTGAATCAGAATCTTCTGGAGTATCAGACCCTCTGATTATTGTGAAGGCAATGGATAGGGTGTTGAGTTGTCTTTTTGTACCATCTGGCATTGAAGTCCTAGCCAGATTGTATGAGCATGCCGTGTTCTCAAAAAAACTTGCATCAACTAAGAAGAGTTGTCTGGCTAAAGTTCATGATAAGCTGCTAAGTTCCCTTAACAACTGCAATGGGCTAGACTGGTGTGTGGCAAAAGGCCGTGGCCATGAGGTGGTTCAGGTGCTGCTTTCTATCCACCGGCTTTCGTCGAAGCAGAAGCAGCAGCAGCAGCAGGGATGTATTAAAGGGATAATTGAGCATGTATGCAGGGAGTATGTTGAAACTGGACAGGATATCAGGGATATAGCTCCTCTAGTTGAAGAATTCCAGATGATGGCATCACCAGCTCCGGATGCCAAGTCGTTGGAGATGGAGACTTATACCCGAGAGCAGCAGGATCCTATGCAGAACAAGGAGTCCATTATCCAGGTGTGCGGGTTACCAAGTATGCCAGACATCCAATGGGAAAAGGAGAAACTTCCTCTGTTCCGAAGTCCATGACTGAGAAATAATCAGAAGAGTGCCAAGGCAGGGCTGAAGAGAAGTAATACGATATTATCATTCATACCAGAACACAGTCTCTTGAAAAGCCCTTCATCTGTCATATCTATGGAAACAAAGGTTTAGGCATGTCGTATTTTTGGTACATCTGCTGGACTAAGATGATATGTTACCTTTGTAGTTCATATACTGTCTGTCTACTGATTGTCATTTTTATTGTTTTAGTTAATATCACTTTTTTTTGGTTTGATAA |
| CL1919.Contig3_All | cellulose synthase 6 [*Paeonia lactiflora*] | ATGTAATGTATTGTAAGTTAGGGGATTATATTGTCTTAGATTAATAAATTTATTTAATTTATTCAGAGTAGGTATTATTTTGCATTCTCTTTGCTGAGATGCGTTGGTATTTCGCAATCAACATGCTCTGCAATTTCTAGAGATTTTTTTTCAGTGGTGATGATTTGAAGTTCAGAGTCTGTAAGAGCAACCACAGAAGAGATCAATCTTTATATATATTCATATAATCTGTCGGCAAAGGGGATTGGAAATCAGTTCCCTCCCCCCCTTTCATCGCGCTTTAGATTGGGGGTGGAGAGCAGTTCTCAGATTTTCTCTGCGATTACAAATGCTTGCAACTTGTAGCTGATATTTTCTTCCAATTTACGATTAAACTTCATCTGGGGTTTGATTATTGGGGGTTTTCTTCTGGGAGGGCGCTTAGATCTGGGACTCAGACTGGCCAATTGTCGATAAATTTCAAGTAAAATCAAAGATGGAGGCGGGTGCCGGATTAGTTGCAGGTTCTCACAACAGGAACGAGCTTGTCGTCATTCGTCGCGATACAGAATCGGCGCGAAAGGCGTTGGAGCAGTTAACTGGGCAAATATGCCAGATTTGTGGGGATGACGTTGGACTTACAGTTGATGGAGAGCTCTTTGTAGCCTGCAACGAGTGTGCCTTCCCCATTTGTAGGACATGCTATGAGTACGAACGCAACGAAGGAAGCCAGGTTTGCCCGCAGTGCAAGACTCGATTCAAACGTCTCAAAGGTTGTGCTAGAGTTGAGGGTGATGAAGATGAAGATGATGTTGATGACCTCGAGAATGAGTTCAATTTTGCAGGCAGGGATAATTCGGATATGCAGTACCTTGCAGAGGCTATGCTTCACGGCCACATGAGCTACGGCCGTGCTGGGGATTCTGACATGCCTCACGTTGTCAATACCATGCCTCAAGTCCCTCTATTGACCAATGGCGACATGGTTGATGATATTCCTCCTGAACATCATGCCTTGGTCCCTTCTTTCAGCGGTGGCGGAGGGAAGAGAGTCCACCCTCTTCCTTTCTTAGATCCCAGTCTCCCAGTGCAACCTAGATCAATGGATCCGTCCAAGGATTTGGCTGCTTATGGTTATGGAAGTGTAGCTTGGAAGGAGAGGCTGGAGAGTTGGAAGCAGAAGCAAGAGAGATTACAGTTGAGGAAGAATGAAAATGGTGGCAAAGATTGGGATAATGATGGAGATGGGCCAGATCTACCCCTGATGGATGAGGCCAGGCAACCATTGTCAAGGAAAATACCTATTGCATCAAGCCGAATCAATCCATATCGAATGATCATAGTAATTCGGCTTGTAGTTCTTGGGTTCTTTTTTCATTACCGTGTCTTGAATCCTGTGAAGGACGCTTATGCATTGTGGCTTATATCTGTGATCTGTGAAATCTGGTTTGCTGTTTCGTGGATTCTTGATCAGTTTCCGAAGTGGCTCCCAATTGACAGGGAAACTTATCTAGATAGGTTGTCCTTAAGGTACGAGAAAGAGGGCCAACCTTCTCAACTTTCTTCAGTTGATATATTTGTGAGTACAGTGGATCCTTTAAAAGAACCTCCTCTGGTTACTGCAAATACGGTGCTGTCCATTCTGGCTGTGGACTATCCAGTTGATAAGCTTTCCTGCTATGTTTCTGATGATGGTGCTGCTATGTTGACATTTGAGGGCTTGTCAGAAACATCTGAGTTTGCAAGAAAGTGGGTGCCTTTCTGCAAAAAGTTCAACATTGAGCCTCGGGCGCCCGAGTTTTATTTTTCTCAGAAAATGGATTATCTTAAGGATAAAGTCGTGACTTCATTTGTGAAGGAGCGAAGGGCTATGAAGAGAGAGTATGAGGAGTTCAAAGTTAGAATTAATGCTTTGGTTGCCAAAGCTCAAAAGGTCCCAGAGGAAGGATGGACAATGCAGGATGGAACCCCTTGGCCTGGAAATGATGTCCGAGATCATCCTGGAATGATTCAGGTTTTTTTGGGTCAAAGTGGAGGACATGACACAGAAGGAAATGAATTACCACGCCTAGTATACGTGTCTAGGGAAAAGCGACCTGGATATAATCACCACAAAAAGGCTGGTGCCATGAATGCTTTGGTCCGAGTCTCTGCTGTTATCACAAATGCACCATACCTGTTGAATTTGGATTGTGATCACTACATCAATAACAGCAAGGCTCTCAGGGAATCAATGTGCTTTATGATGGATCCATTGCTAGGAAAGAAAGTTTGCTATGTGCAGTTCCCGCAGAGATTTGATGGGATTGATAGGCATGATAGATATGCCAATAGGAACACTGTTTTCTTTGATATCAACATGAAAGGCTTGGATGGGATTCAAGGCCCAATATATGTCGGGACTGGATGTGTCTTTAGAAGGCAAGCGCTCTATGGGTTCGATGCTCCAAAAACAAAGAAGCCTCCAACAAGAACCTGTAATTGCTGGCCAAAGTGGTGCTGTTGTTTTGGGAGGAAGAAAAAGAAGGCTACAAAGCCCAAGTCTGAGAAGAAGAATAAAAATTTTATGAGAGGAAATTCGGGAGCACCCGTGTTTGCTTTGGAGGGCATTGAAGAGGGAATTGAAGGCATTGAGAGTGAAAGAGCAACTGTGGTCTCGGGGCAGAAATTGGAAAAGAAGTTTGGGCAGTCTTCTGTGTTTGTTGCATCAACCCTACTTGAAGATGGAGGGACACTGAATAGTGCTAGTCCTGCATCTCTGTTAAAGGAAGCCATCCATGTGATAAGCTGTGGTTATGAAGACAAAACTGATTGGGGAAAAGAGGTTGGCTGGATCTATGGTTCAGTTACAGAAGATATTTTGACAGGTTTTAAGATGCACTGCCATGGATGGAGATCCATATACTGCATACCTAACAGACCAGCTTTTAAAGGGTCTGCGCCTATTAATCTTTCTGATCGTTTGCACCAAGTCCTTCGATGGGCCCTTGGTTCTGTTGAAATTTTCTTGAGCAGACACTGTCCCCTCTGGTATGGTTATGGATGTGGTCTGAAATGGTTAGAGCGTCTCTCTTACATCAACGCAACTATATACCCTTGGACGTCAATTCCTTTGTTGGCTTACTGTACCTTACCTGCAGTGTGCTTGCTCACTGGAAAATTCATCACTCCAGAGCTTACCAATGTTGCGAGTTTGTGGTTTCTGTCTCTCTTCATCTGTATTTTCGCAACTGGTATCATGGAAATGAGATGGAGTGGTGTCGGAATTGACGATTGGTGGAGAAATGAGCAGTTTTGGGTCATCGGAGGTGTTTCGGCTCATCTTTTTGCAGTCGTACAAGGACTGTTGAAAGTACTCGCCGGTATAGACACAAACTTCACTGTCACTTCAAAATCCGGCGATGACGATGACTTCTCCGAGCTCTATGCATTCAAATGGACAACGCTTCTCATCCCCCCAACCACGCTTCTAATCATCAACCTGATTGGAGTGGTTGCCGGAATATCAAATGCAATCAACAACGGGTACGAATCGTGGGGACCTTTGTTTGGTAAGCTCTTCTTTGCCTTCTGGGTGATTGTCCATCTCTATCCTTTCCTCAAAGGTCTACTTGGACGAAATAACAGAACGCCGACCATTATCATTGTGTGGTCGATTCTTCTTGCTTCGATTTTCTCTCTTTTGTGGGTTCGAGTTGATCCTTTCTTGGCCAAGTCAGATGGTCCTGTACTTGAAGAATGTGGGCTGGACTGCAATTAATCAACACTCAACTCTGGTCCAGCAAAACAAGTTTATAATATACATTCTGTCACACGTTTGGCACTGCCTTCGGAAGTTCTCGAAGCCTGTTGTTGTGAATCGGTTCCTTCAAAGAGGGGAGGGTATCGGAGAGTGCTGGGAAATTGATACTGAAGATACGAAAAGTGGTAAATTCTTTATTATTTGATGTGTTTTTTTTTATTTTTTTTTTAATGTTGAGAACAGAGATGTGCAGAGGATGGAGTTATTTGCAATCAAGTTTAAGGTTTAGGTAAATTAGAAATGGGTTTTGTGTTGTACGAGGGTTTGAGAAGTTAAGAATCAGTTGGTGTGGTTGGAAAGAAAAAAAAAATGTTTGTAAAAGAGGTATTTATTGTCACGTGGGGTTTTTGTATTTGTAATAAGTTTTAGGATTTAGGAAGCATTGTATTGTATGTTGGGGAATGGAATTTGTAATTTGTTTTAAACTGAGCCTCATTTGGAAGAGAATAA |
| CL1952.Contig1_All | cellulose synthase-like protein D3 [*Ziziphus jujuba*] >XP_015878547.1 cellulose synthase-like protein D3 [*Ziziphus jujuba*] | ATGTAATTTTTTATGGGTTAGTGTTACAGAAAGTTTATGCTTTGTTTGGTTGCTGAGAAAATGTGTAAAAGGTAAGGGGTATATAATTTTAGATCTTAGTTTTTTCACAAGCCATGAATAAAAGTAAGGAGAATATTCACGTGGGTTTAATTAGATTTAACATTCAATTTTCTTTAAACCAAACGGCGGTAAATGTTCATGTATATATATTTGTTCATAATTTTATGGAATTACAGAATCTGGTAATAAACCTGTATACTATTCAAAACCATGTTGATTAAGTTCATAATTTTATTGAATTACAGGATCTGGGGGATATATCTGTTCTGGTGGTAAAACCATGGCATCAAGAATGTACAAGGCAAGCCGATCATCTTTATCCACATCCTCAGATGCACCCGATGGGCAAAAGCCTCCGCTTCCCCCCACTGTGACATTTGGCCGGAGAACTTCATCTGGTCGGTACATTAGCTACTCAAGGGATGATCTCGATAGTGAACTTGCCAGTAGTGAGTACATGAACTACACGGTACACATACCCCCTACACCCGACAATCAGCCCATGGAAACAACAATGGAGCAAAAGGTTGAAGAGACACTTACATCGAGTTCACTCTTTACAGGTGGATTTAATAGTGTTACAAGAGCCCATCTCATGGACAAGGTGATTGATTCTGAAGCAAGCCATCCCCAGATGGCTGGTGCGAAAGGATCTTCATGTGCCATTCCAGGGTGTGATGGTAAGGTCATGAGTGATGAACGCGGTGTAGATATACTCCCTTGTGAATGTGATTACAAGATATGTAGGGAGTGTTATAGGGAAGCAGCTAGAGCAGGTGATGGGATTTGCCCTGGATGCAAGGATCCATACAAGAACACGGATTTGGATGAGGTGGCTGTGGATGATAGTCGTCCGCTACCACTTCCGCCTCCAGGTGGGATGTCCAAAATGGAGAGGCGGCTTTCATTGATGAAATCAACGAAGTCTGTGCTGATGAGGAGTCAGACAGGGGATTTTGATCACAATCGGTGGCTGTTTGAGACCAGAGGTACTTATGGGTATGGAAATGCTATATGGCCCAAGGATGGTGGTTTTGGGGATGGGGATGGAAATGGGAATGGGAAAGATGACGATGTAGTTGAGGCAAGGGATCTAATGAACAAACCATGGAGGCCACTAACTCGCAAATTAAAGATACCTGCTGCTGTTCTCAGCCCATATAGGCTTTTGATATTTATTCGATTTGTTGTTCTTGGATTGTTCTTGCATTGGAGGATAACCAACAAAAATGAGGATGCCATCTGGCTGTGGGGGATGTCCGTGGTTTGTGAGCTCTGGTTTGCATTCTCTTGGCTTCTTGACCAGCTTCCCAAGCTGTGCCCTATCAATCGCGCTACTGATCTAAATGTTTTGAAAGAGAAGTTTGAGTTGCCCAGCCCCAACAACCCCACTGGAAAATCTGATCTCCCAGGCATAGACATCTTTGTTTCTACTGCAGATCCTGAGAAAGAACCCCCACTTGTCACTGCAAACACCATCTTGTCCATTGTTGCTGCTGATTACCCTGTTGAAAAGCTTGCTTGCTATGTTTCTGATGATGGAGGTGCACTTCTAACTTTTGAGGCCATGGCAGAAGCTGCCAGCTTTGCTAATATATGGGTTCCCTTTTGCCGTAAACATGATATTGAGCCCAGGAATCCAGAGACTTATTTCAGTTCAAGGAGGGATCCTTATAAGAACAAAGTTAAACCAGATTTTGTTAGGGATCGTAGACGGGTAAAGCGTGAGTATGATGAGTTCAAGGTACGGATTAATGGCTTGCCCGAATCTATCCGTCGGAGGTCTGATGCCTATCATGCTCGGGAGGAGATCAAGGCTATGAAGCTTCAGAGACAAAACAGAAATGATGAACCCGTAGAGAGTGTGAAGATTCCAAAAGCAACCTGGATGGCTGATGGAACCCATTGGCCTGGGACCTGGATTCATTCTTCAACTGAGCACTCTAGGGGTGATCATGCCGGAATAATTCAGGTCATGTTGAAGCCTCCGAGTGATGATCCACTACTAGGTATTGCCGATGATACCAAGCTAATTGACCTTACAGATATTGATATCCGTCTTCCCCTGCTAGTTTATGTTTCCCGTGAGAAGCGTCCTGGCTACGATCACAACAAGAAGGCAGGGGCCATGAATGCTCTAGTTCGAGCATCAGCCATCATGTCCAATGGTCCCTTTATTCTCAACCTTGACTGTGATCATTACATTTACAACTCCCAGGCTATGAGGGAAGGTATGTGTTTCATGATGGATCGAGGCGGTGACCGCATTTGCTATGTCCAGTTTCCTCAGAGGTTTGAAGGTATTGATCCTTCTGATCGCTATGCTAATCACAACACCGTGTTCTTTGATGTCAACATGAGGGCCCTTGATGGGCTACAGGGACCTGTCTATGTGGGAACAGGATGCCTCTTTCGAAGGATTGCTCTTTATGGATTTGACCCTCCTCGTGCAAAAGAACACCACCCTGGTTGCTGCAGCTGCTGCTTTGGTCGTCGCAGAAAGCACGTCACAATTGCCAACACCCCAGAAGAGCATCGTGCCTTAAGAATGGGTGATTCAGATGATGAGGAGATGAACCTTTCCCTTCTCCCAAAAAGGTTTGGCAACTCAAGCTTCCTCATTGATTCAATCCCAGTAGCAGAATTCCAAGGTCGTCCCCTCGCTGATCACCCAGCCGTAAAACATGGAAGGCCACCTGGTGCTCTCACCATTCCAAGAGAGCTGCTTGATGCATCAACTGTTGCAGAAGCTATTAGTGTCATCTCTTGCTGGTACGAGGATAAGACCGAGTGGGGAGATCGTGTAGGATGGATTTACGGGTCGGTGACAGAAGATGTTGTTACTGGATACAGAATGCACAACAGAGGATGGAAGTCGGTTTACTGTGTTACCAAACGTGATGCATTTCGTGGGACTGCTCCTATCAACCTCACAGATCGTCTTCATCAGGTCTTGCGTTGGGCTACTGGTTCAGTTGAGATTTTCTTCTCTCGAAACAATGCTCTTCTAGCTAGCCCAAGAATGAAAATTCTTCAGAGGATTGCATATTTAAACGTTGGAATCTACCCTTTCACTTCGTTCTTTCTCATTGTCTACTGCTTCTTACCGGCACTTTCTCTCTTTTCCGGCCAATTCATCGTCCAAACGTTAAACGTTACTTTCCTTGTTTACCTCCTGGTTATCACTCTCACTCTCTGCATGCTTGCTGTTCTTGAAATCAAATGGTCTGGCATTGAACTAGAAGAATGGTGGAGAAATGAACAGTTTTGGTTGATAGGAGGCACTAGTGCTCATCTTGCTGCTGTGCTTCAGGGTCTACTTAAAGTCATTGCCGGGATTGAAATCTCCTTCACCTTGACTTCAAAATCGGGCGGTGATGATGTCGACGACGAGTTTGCTGATCTCTACGTAGTGAAATGGACTTCTCTGATGATTCCGCCCATCACCATTATGATGACCAACTTAATAGCTATAGCAGTGGGAGTTAGTAGAACGATATATAGTGTGATACCTCAGTGGAGTCGTCTGATTGGTGGGGTTTTCTTCAGTTTCTGGGTTTTGGCTCATCTTTATCCTTTTGCCAAAGGTCTTATGGGAAGAAGAGGAAGAACTCCGACCATTGTTTTTGTTTGGTCTGGACTTATTGCTATCACGATATCGCTGCTTTGGGTGGCTATTAGTCCACCGGATGGTACTGATCAAATTGGTGGCTCGTTTCAGTTCCCTTGAGTTGATTATGGGTTGATCAATTTCTCTCTTGTAGTTATGTTCTTTGTGATATCGTTTAATTGATTCTTTTCACAAGGTGTTTTCATGTTTTTTTAAAGAGACTAGGTTTTTTATTCAGAGATGTTGGGACGATGAAAAATCAAGTAATATGCATTTTGTTGGCGTAA |
| CL1738.Contig1_All | UDP-glucose 6-dehydrogenase 1 [*Manihot esculenta*] >XP_021634637.1 UDP-glucose 6-dehydrogenase 1 [*Manihot esculenta*] | ATGAGTGTGGGTATTTAAGGATGTCTTGATCCCATAGATTTCCTGCACAAGTAGTAATCTCTACAAGTTGTGATCTATCTTACACTCTAGACCACTCCTTTTACCTCTATTTCCTTCTGTCCATATTTGAAGAAAATGGTGAAGATATGTTGCATTGGAGCTGGATACGTTGGTGGTCCTACAATGGCAATAATTGCACTCAAGTGCCCCTCAATTGAAGTAGCTGTTGTCGATATCTCTGTTGCTCGGATTGCAGCCTGGAACAGTGACCAGCTCCCTATTTATGAACCGGGTTTGGAAGAGGTGGTGAAGCAGTGCCGCGGAAAGAACCTCTTTTTCAGTACTGCTGTGGAGAAACATGTCTCTGAGGCAGATATAATCTTTGTTTCAGTTAACACCCCAACGAAAACTCGTGGCCTTGGAGCTGGCAAAGCTGCAGACTTGACATATTGGGAGAGCGCAGCCCGAATGATTGCCAATGTCTCAAAATCTGACAAGATTGTTGTTGAGAAATCAACAGTCCCGGTGAAAACAGCTGAGGCAATTGAAAAAATTCTGACCCATAACAGCAAAGGAATCAACTATCAAATTCTCTCAAATCCAGAATTCCTCGCTGAGGGAACTGCAGTCGAAGACCTCCTTAGTCCAGACCGAGTTCTCATTGGAGGGAGGGAAACCCCTGGTGGCCAAAAGGCAATCAAAGCACTAAAAGATGTTTATGCTCACTGGGTGCCAGAAGACAGAATCATTACCACCAATCTCTGGTCGGCTGAGCTCTCGAAACTAGCCGCCAATGCCTTCTTGGCTCAGAGGATTTCATCTGTCAATGCAATGTCAGCTCTTTGTGAGGCAACTGGAGCAGATGTTTCTCAGGTTTCCCATGCAGTTGGTAAGGACACCAGAATTGGCCCCAAGTTCCTCAATGCCAGTGTTGGTTTTGGTGGCTCTTGCTTTCAGAAGGACATTCTCAACTTGGTCTATATTTGTGAATGCAATGGCCTCATCGAAGTTGCGAATTACTGGAAACAAGTGATTAAGGTGAATGACTACCAAAAGAACCGATTCGTCAACAGGGTAGTTGCTTCAATGTTCAACACAGTTTCAGGCAAAAAGATTGCTGTTCTTGGCTTTGCTTTTAAGAAGGATACCGGTGACACAAGAGAAACTCCAGCTATTGATGTGTGCAAGGGTCTGTTGGGGGACAAGGCGAGGTTGAGCATCTACGATCCTCAAGTCACCGAGGATCAAATCCAGAGGGATCTCTCAATGAACAAATTTGATTGGGACCATCCGGTTCACCTTCAGCCAATGAGCCCAACTTGTGTGAAACAAGTGAGTGTGGTCTGGGACGCCTACGAAGCGACCAAAGATGCTCATGGGATCTGTATTCTTACAGAGTGGGACGAGTTCAAGGAGCTGGATTACCAAAAGATTTTTGATAACATGCAGAAGCCTGCATTTGTGTTTGATGGGAGGAACGTGGTGAATGTGGAGAAGCTGAGGGAGATTGGATTTATTGTCTACTCAATTGGGAAGCCGTTGGACCCTTGGCTCAAGGACATGCCTGCTGTTGCTTAGGGGAATGGAAAGAAGCTTAATGTGCTGGAGTGGTTTGTGGAGGGGTTGAGTTTAGTCAAAGGAACTTTCATCCTATACTATTTACCATTGTTATTATTTGTCATTTTTGCCTTTCAGTTGCTTCTAGCTTTTGCTATTATTCTGTTGTGTCTCACTTTTTTTTTTCCTCTGAATATCTTTAGTTAGAAGAATATCTGGATTTGTAA |
| CL1738.Contig2_All | PREDICTED: UDP-glucose 6-dehydrogenase 1 [*Vitis vinifera*] >CAN62897.1 hypothetical protein VITISV_020300 [*Vitis vinifera*] | ATGGTCAAAATTTGTTGCATTGGAGCCGGGTATGTCGGAGGCCCAACAATGGCAGTGATTGCACTCAAGTGCCCATCCATTGAAGTAGTTGTTGTGGATATCTCCGTCTCACGTATCACAGCGTGGAACAGTGAGCAGCTCCCAATCTATGAGCCTGGTCTCGAAGGTATAGTGAACGAGTGCCGAGGCAAGAACCTCTTCTTCAGCACTGATGTGGAAAAACACGTAGCTGAAGCCGATATAGTCTTTGTTTCCGTCAACACCCCAACCAAAACCCGTGGCCTTGGAGCAGGCAAAGCAGCCGACTTAACATATTGGGAGAGTGCTGCTCGCATGATTGCCGACGTGTCAAAATCTGACAAAATTGTAGTCGAGAAATCAACTGTCCCAGTTAAAACAGCAGAAGCAATAGAAAAGATTCTAACCCACAACAGCAAAGGTATTAAATTCCAAATCCTCTCAAACCCGGAGTTTCTCGCTGAAGGAACTGCAATTCAGGATCTATTCAACCCGGACCGTGTACTCATCGGGGGCAGGGAAACCCCAGAAGGTCAAAAAGCAATCCAAGCATTGAAAGATGTTTACGCCCATTGGGTTTCTGACGACAGAATCATAACTACTAATCTCTGGTCTGCTGAGCTCTCGAAGCTTGCTGCGAATGCCTTCTTGGCACAAAGAATCTCGTCTGTTAACGCCATGTCTGCTCTTTGTGAAGCTACTGGAGCTGATGTTACTGAGGTTTCATATGCTGTTGGCAAGGACACTCGTATCGGACCCAAATTTCTAAATGCCAGTGTTGGATATGGCGGGTCTTGTTTCCAGAAAGACATCTTGAATCTAGTCTACATTTGTGAATGCAATGGTCTTCCAGAAGTAGCTGAATACTGGAAGCAAGTGATCAAGGTGAATGATTATCAAAAGAACCGCTTCGTCAACCGTGTCGTTGCTTCTATGTTCAACACAGTTGCGAACAAGAAGATTGCAATTCTAGGGTTTGCTTTTAAGAAAGATACCGGTGATACGAGGGAGACTCCAGCTATTGATGTTTGCAAAGGGCTGTTGGGTGACAAGGCGATGTTGAGTATATACGACCCACAAGTCACCGAGGATCAGATTCAAAGGGATTTGTCGATGAAGAAGTTTGATTGGGATCATCCTATTCATCTTCAGCCAATGAGTCCTACCACTGTGAAGAAGGTTAGTGTGGTTTGGGATGCCTATGATGCAGCAAAGGATGCTCACGGTATTTGTATTATGACAGAGTGGGATGAGTTCAAGAGTTTGGATTACAAGAGGATATATGATAATATGCAGAAGCCGGCCTTTGTGTTTGATGGGAGGAATATCGTGAATGTGGATATGTTGAGGGAGATTGGGTTTATTGTGTATTCTATTGGGAAGCCTTTGGATCCGTGGCTCAAGGACATGCCTGCTGTGGCATAAGATGACAGAAGCCAGAGAGATGCAGTTCAATTCTTTGAATTTTTTTTTCCTGGATGTATCTTATTTTTACCTTCAAAACCGTCTTGTTTTAGGATCATGAGTGTTTCATTGGAAACCTCATCTTACCTCTGCCTGCTTTTATTTTTGTTGTTATATTATACAATAATTATGAGAATTTTCATTAA |
| Unigene21241_All | PREDICTED: UDP-glucose 6-dehydrogenase 1 [*Vitis vinifera*] >CAN62897.1 hypothetical protein VITISV_020300 [*Vitis vinifera*] | ATGTTGCTAGACACACTTAACTAAAGTTGCATATCAAATCAAACAAAAATCAAATTAAGTTCGGTAACAAACTCAAGGACGAGGATATTACAATCAAATACATTGTCAGACAAATCGTAAAGTCTCTTACTCCAGACAAGTAAGAAACATCAGTAAACAAAGTAGTAAAGCATCAAACAGACTGTCCCCATACTATGGTTCTCCAAACAAGGCCACCCTCAAATTTCTTCCACTCTATTGGTGCACGTCTCTTAAGCCACAGCAGGCATGTCCTTGAGCCACGGATCCAAAGGCTTCCCAATAGAATACACAATAAACCCAATCTCCCTCAACATATCCACATTCACGATATTCCTCCCATCAAACACAAAGGCCGGCTTCTGCATATTATCATATATCCTCTTGTAATCCAAACTCTTGAACTCATCCCACTCTGTCATAATACAAATACCGTGAGCATCCTTTGCTGCATCATAGGCATCCCAAACCACACTAACCTTCTTCACAGTGGTAGGACTCATTGGCTGAAGATGAATAGGATGATCCCAATCAAACTTCTTCATCGACAAATCCCTTTGAATCTGATCCTCGGTGACTTGTGGGTCGTATATACTCAACATCGCCTTGTCACCCAACAGCCCTTTGCAAACATCAATAGCTGGAGTCTCCCTCGTATCACCGGTATCTTTCTTAAAAGCAAACCCTAGAATTGCAATCTTCTTGTTCGCAACTGTGTTGAACATAGAAGCAACGACACGGTTGACGAAGCGGTTCTTTTGATAATCATTCACCTTGATCACTTGCTTCCAGTATTCAGCTACTTCTGGAAGACCATTGCATTCACAAATGTAGACTAGATTCAAGATGTCTTTCTGGAAACAAGACCCGCCATATCCAACACTGGCATTTAGAAATTTGGGTCCGATACGAGTGTCCTTGCCAACAGCATATGAAACCTCAGTAACATCAGCTCCAGTAGCTTCACAAAGAGCAGACATGGCGTTAACAGACGAGATTCTTTGTGCCAAGAAGGCATTCGCAGCAAGCTTCGAGAGCTCAGCAGACCAGAGATTAGTAGTTATGATTCTGTCGTCAGAAACCCAATGGGCGTAAACATCTTTCAATGCTTGGATTGCTTTTTGACCTTCTGGGGTTTCCCTGCCCCCGATGAGTACACGGTCCGGGTTGAATAGATCCTGAATTGCAGTTCCTTCAGCGAGAAACTCCGGGTTTGAGAGGATTTGGAATTTAATACCTTTGCTGTTGTGGGTTAGAATCTTTTCTATTGCTTCTGCTGTTTTAACTGGGACAGTTGATTTCTCGACTACAATTTTGTCAGATTTTGACACGTCGGCAATCATGCGAGCAGCACTCTCCCAATATGTTAAGTCGGCTGCTTTGCCTGCTCCAAGGCCACGGGTTTTGGTTGGGGTGTTGACGGAAACAAAGACTATATCGGCTTCAGCTACGTGTTTTTCCACATCAGTGCTGAAGAAGAGGTTCTTGCCTCGGCACTCGTTCACTATACCTTCGAGACCAGGCTCATAGATTGGGAGCTGCTCACTGTTCCACGCTGTGATACGTGAGACGGAGATATCCACAACAACTACTTCAATGGATGGGCACTTGAGTGCAATCACTGCCATTGTTGGGCCTCCGACATACCCGGCTCCAATGCAACAAATTTTGACCATTTTTCTTTTCTATCTATAACCTGGATCAAAAGAAATTTACCAATCAGCAGATGCACCAATTTCCAGATTTTAGCAAGATCAAAAAGTATATCCAAGAAATAACCCACTACTCATGTAATAAGAAAACAATACATGTTAAGTAGATTTCGCTTCATCAGAAAAATAGGACTGACATTTCTTTTGGGAAAATATATACTAGCAACAAGCACTCTCACAGGGTTTTGTGATTCAATCCTTTCTGGCTCCTAAACTTCCAAAAATAATCCTAATAATAAAACATATTAATTAAATGAATTATTTCACATAATGGGTATATTAAGTATTTAAAAAATATTAATAACTTCCCAGGACTCATCCTCATAACTTCACCTATAACTCCCTCTAACTTCCTCTCTTCGCAACTTCCTTACTACAACTCCCGCTAATTGGACATGCACTGCATTTACAGGAATATTAGTACATTACTTACATAGCATAGCAACGCAATTTAGGAACCTTTTATCCAAATCATTCTTATGTATAGACGCATCATTGCACCTCAACGTCCAAAACAGCAAAACGAACAATTGTCATAAGTAATTCTAATGCGCAATCATCTCCAAACTATAGAGTTGCATAAAATAAGGCCAGATGTCTGAGCCTCAGATTCAATTTCGTTTCATGAGTGCTGTCATCTGTGATGCTACGATACGCGAGAAATGATTGGAATTTTTTAAAAATGAAAATGCATCCTAATGAAGTTGCGGTGGATTCAACCAATAATTTTTCAACATGTTTCATCACTTCAAGTACTTGAGAAGGCTGGCTTAAAACACAATCGTGTTTATTCATCCTGGAAAAGGATGAGACAAACCAATTCCAAGAATATAGAGACGCATAAAACGATTAGCAATCAATTCCAGTTCAAATAAATCGACCGACTGAATAATCAAATGTGAGAGTACTTACAAACTAAGATACCATCAGATCTACACCAATGACCATAGATTTACTAAATATAGAAACATAAACCACGGATTACGAGTACACAAGCACGAGCATTACAGCAGATCTTGAATCTTCAGAAATTGATTAAAGTTTTCATAGGATTTACTAGATCCATCAACAAACTGATCACTTAAAAACACTAATGCTACTTTTTTACGCGAAATGGAGAAATCATAAAGAAGAGAAATCGAGTTCAGATCGAGATTGAAAATGGCAACAGATCTGAAGATCATGCATTCCAAACCATAAAAAATTATAACAGAACACAAACAGGAAAGTGAAATTTAATACAAGTTGAGATAAAAAAAAAATCAGAACAGAAAAAAGCACACCACCAATGCAAAATGAAACGAAATTGCAAACATATCATGAAACCAAAATCTTTCCTGAAGTTTCAGGTTCGGAATTGAGATCGAGCTTACCTCCCGGATTGCTCTTTTGGCCTTCTCCAATGAAGTTAGAGAGAGAATGCAGATAGGATAGAGATTAGAATTCAAACAGAGTATTTAGAAAGGAAGTTGAGAAGATTTAGTGAGAGAAAGAGAGGGACGATCTTGCGGCTTCCTTCTGCGAGAGCGGGGAGGTCCGCCTTAA |
| CL1051.Contig5_All | PREDICTED: UDP-glucose 4-epimerase GEPI48 [*Vitis vinifera*] >CAN63477.1 hypothetical protein VITISV_009458 [*Vitis vinifera*] >CBI34495.3 unnamed protein product, partial [*Vitis vinifera*] | ATGGCGAAAAGCGTCCTGGTGACCGGTGGAGCGGGTTATATCGGGAGTCACACCGTTCTTCAGCTATTGTTAGGAGGATTCAAGACCGTTATCATCGACAATCTCGACAATTCTTCTGAAATCGCCGTCAAGAGAGTCACCGAACTTGCTAAAGAACATGGCCAAAATCTTTCATTTCATAAGATGGACCTCCGGGACAAACCAGCACTTGAAAAACTTTTTGCTTCAACAAAATTTGATGCTGTTATACACTTTGCTGGACTGAAAGCAGTTGGTGAAAGTGTGCAGAAACCATTGCTTTACTATGACAACAACCTTATTGGTACAATTGTTCTTTTGGACGTCATGGCTGCTCATGGATGTAAAAAGCTTGTGTTTTCATCATCAGCTACTGTTTATGGTTGGCCAAAGGAGGTTCCATGCACAGAAGAGTTCCCTCTGTCTGCAGCTAATCCATATGGGCGAACCAAGCTTATCATTGAAGAGATGTGCCGTGATATCTATGCATCAGACTCTGAATGGAAAATCATATTGCTAAGATACTTCAACCCAGTCGGAGCTCATTCTAGTGGGTATATTGGTGAGGATCCCCGGGGTATTCCAAACAACCTTATGCCCTTTGTGCAGCAAGTCGCTGTTGGCAGGCGACCAGCTTTGACAGTTTTTGGCACCGACTATTCAACAAAAGATGGCACTGGGGTACGTGATTACATTCACGTTGTTGATTTAGCAGATGGGCACATTGCTGCTTTGTGCAAGCTATTCGATTCCAAGATAGGTTGTGAAGTGTACAACTTGGGAACAGGTAAAGGAACATCAGTTTTGGAAATGGTTGCAGCTTTTGAGAAGGCGTCTGGAAAGAGAATTCCTATCACAAAGGCTGGGCGGCGAGCTGGTGATGCCGAGGTTGTTTATGCGTCTACAGCAAAAGCAGAACGTGAATTGAACTGGAAGGCGAAATTTGGTATCGATGAGATGTGTAGGGATCAATGGAACTGGGCTAGCAAGAATCCTTATGGCTATGGATCCCCTGACGCTAAAAACTGAAAGAAGTTATGCCTTATATATATATATATATATATATATATATATATATATATATATATATATATATATATAAGGCATAACTTCTTTCAGTTTTTAGCGTCAGGGGATCCATAGCCATAAGGATTCTTGCTAGCCCAGTTCCATTGATCCCTACACATCTCATCGATACCAAATTTCGCCTTCCAGTTCAATTCACGTTCTGCTTTTGCTGTAGACGCATAAACAACCTCGGCATCACCAGCTCGCCGCCCAGCCTTTGTGATAGGAATTCTCTTTCCAGACGCCTTCTCAAAAGCTGCAACCATTTCCAAAACTGATGTTCCTTTACCTGTTCCCAAGTTGTACACTTCACAACCTATCCTGGATTCAAATAGCTTGTTCAATGCAGAAATGTGCCCATCTGCCAAATCAACCACATGAATGTAATCGCGTACCCCAGTGCCATCCTTGGTTGAGTAGTCATTTCCAAAAACTGTTAGTGCCGGTCGCCTGCCAACAGCAACTTGCTGGACAAAAGGCATAAGATTGTTTGGAATGCCTCGGGGATCTTCACCAATATACCCACTAGGATGAGCACCGACCGGGTTGAAGTATCTTAGTATTATGATTTTCCATTCAGAGTCCGAACCGTAGATATCACGACAAATCTCTTCAATGAAAAGCTTGGTTCGTCCATATGGATTTGTTGCAGACAAGGGGAACTCTTCCGTGCAAGGCACCTCCTTTGGCCAACCATAAACAGTAGCTGATGAAGAAAAAACAAGCTTTTTACATCCATAAGCAGCCATGACTTCCAACAGAGCAATTGTAACATTAAGGTTGCTATTATAGTATAGCAATGGTTTCTGTACACTTTCACCAACTGCTTTCAGTCCAGCAAAGTGGATGACAGCATCAAATTTTGTTGAAGCAAAGAGTTTCTCAAGTGCTGGTCTGTCCCTTAGGTCCATCTGGACAAAGGTGAGATCTTGACCATATTGACCGGCCAGTTCGGTGACTCTTTTAATGGCGATTTCGGAAGAATTGTCGAGATTATCGACTACAACGGTCTTGAATCCTCCTAGTAAAAGCTGAAGAACAGTGTGGCTGCCGATGTAA |
| Unigene12925_All | PREDICTED: trifunctional UDP-glucose 4,6-dehydratase/UDP-4-keto-6-deoxy-D-glucose 3,5-epimerase/UDP-4-keto-L-rhamnose-reductase RHM1 [*Vitis vinifera*] | ATGGGTACGTATACCCCCAAAAACATTCTTATAACGGGAGCTGCTGGTTTCATAGCATCCCATGTTGCCAACCGGCTCATCCGGAATTACCCTGATTACAAGATTGTTGTGCTTGACAAGCTTGATTATTGTTCGAATTTGAAAAACCTTCTCCCCTCTAAATCGTCCCCCAACTTCAAGTTTGTCAAGGGGGACATTGGCAGTGCTGATCTGGTCAACTTCCTCCTCATCACCGAGTCCATTGACACAATAATGCATTTTGCTGCCCAAACCCATGTAGACAACTCCTTTGGTAATAGCTTTGAGTTTACCAAGAACAATATCTATGGGACTCACGTGCTTCTAGAAGCCTGCAAAGTTACTGGCCAGATCAGGAGGTTCATCCATGTCAGTACAGATGAAGTGTATGGTGAGACAGATGAAGATGCCATTGTAGGAAACCATGAGGCCTCTCAACTCCTCCCAACAAACCCATATTCCGCGACGAAAGCTGGAGCAGAAATGCTTGTTATGGCTTACGGTAGGTCATACGGCTTACCTGTGATAACTACCCGAGGAAACAATGTTTATGGACCCAATCAATTTCCCGAAAAGTTGATTCCAAAGTTCATTCTTTTAGCCATGAGAGGGAAGCCTCTTCCTATTCATGGGGACGGTTCTAATGTGCGTAGCTATCTGTATTGTGAGGATGTTGCTGAGGCATTTGAAGTTGTTCTTCACAGGGGAGAAGTTGGCCATGTTTACAATATTGGGACAAAGAAGGAAAGGAGAGTGATTGACGTGGCCAAAGACATATGCAGACTTTTCTCAATAGACGCAGAGACAAGCATCAAGTTAGTAGAGAACAGACCATTTAATGATCAGAGGTACTTTCTTGATGATGAGAAGCTGAAGAACTTGGGCTGGTCCGAAAGAACTACGTGGGATGAGGGGTTAAAGAAGACCATGGATTGGTACATCAAGAATCCTGATTGGTGGGGCGATGTGAATGGAGCATTGCTACCTCATCCACGAATGCTAATGATGCCCGGTGGTATCGAAAGACACTTTGACGGGTCTGAAAATGACACTTCCTCTCACGTATCAAGCACTCCTAACCAAACCCGAATGGTGGTTCCACCTTCCAAAACCAGCAGCTCTCCTCGAAAACCATCTTTTAAGTTCTTGATTTACGGCAGGACCGGGTGGATTGGTGGTCTACTCGGGAAACTCTGCGAGAAACAAGGGATTCCTTACGAGTATGGAAAAGGGCGTCTAGAAGAACGATCTACCCTTGTATCAGACATTCAGAACATCAAGCCAACTCATGTTTTCAATGCAGCTGGTGTGACTGGTAGACCCAATGTTGATTGGTGCGAATCTCACAAAACTGAAACCATTCGCACCAATGTAGCGGGTACACTTACTTTAGCTGATGTTTGCCGAGACCACGGACTTTTAATGATGAATTTTGCTACCGGCTGTATTTTTGAGTACGATGCTGCTCATCCAGAAGGTTCAGGCATTGGTTACAAAGAAGAAGACAAACCTAATTTCTTTGGTTCTTTCTATTCAAAAACCAAAGCCATGGTGGAGGAGCTATTGATAGAATATGAAAATGTATGCACGCTTCGAGTCCGAATGCCAATATCATCAGACCTGAACAACCCGCGAAACTTCATTACGAAGATTTCTCGGTACAACAAAGTGGTGAACATTCCAAATAGCATGACGGTGTTGGATGAGCTTTTACCGATTTCAATCGAGATGGCGAAGAGAAATTGTAAGGGTATATGGAACTTTACAAACCCTGGAGTTGTGAGTCACAATGAGATCCTGGAGATGTACAAGAGCTATATTGATCCGAGTTTCAAGTGGGCGAACTTTACGTTGGAAGAGCAAGCTAAGGTTATCGTTGCAGCAAGGAGTAACAATGAGATGGATGCGTCTAAGTTGAAGAATGAGTTCCCGGAGTTGTTGTCAATTAAGGAGTCGCTGATAAAGTATGTGTTTGAACCCAACAAGAAGAATTTGGCAAAGGATTAGTAATGAGTCTGGGTTTATCTTCATAATTTTTGTCAGTTTCAGTCATTATTCATTGTTCTGTTGTCATAGATAGAGATCATCATTCTACTACTACTACTTTTTTTTTTTTTCCATTTATATTTTAGTTTGCTTATGTTATTATTATTATTTTTTCTAGAAGAACTTATGAATCAATTTTTTATATGTTATAATATCTAAATATCATTTGTAGTTTTGTGTGTAATTTTCTTTTTGTTTTTTAGTTAATTTAATTTTAA |
| Unigene14343_All | PREDICTED: UDP-glucose 4-epimerase GEPI48 isoform X1 [*Vitis vinifera*] | ATGCATAGACTGCTCTAATTTTCTCGTGGGTTGCTTGTCTTACTAGCCCAATTCCATTGATCCCGGCACATCTCATCGATCCCAAACTTTGCCTTCCAGTTCAATTCACGTTCTGATTTTTCGTTGATGCATAA |
| CL1736.Contig1_All | probable galacturonosyltransferase 4 [*Arachis duranensis*] | ATGCAGTACCACATTTCTCTACGGATGACAACAATAAATATAGAATAGAATTGAAGCTCCAAATCTACTGCACCAAAAAAGAACAAAAATTTGTAACAAAAAGAATGAAAAAAGAACGAACTAAATTTGGGGTTGGGAGCAATATTTAACATCGTGAGATACCATTTCGCACCAATTCAAGGGTTGATATTGCATTCTCGTAAATACACATGATCGTAGTTGATGAATTTTGCCCAGAAGTTCCGGAACTTGGGCATCCCTATCTCTAGCCAGGGTTTCAGGTTTCCATTGTAGTGGATCACAGCTGCTCGTTCAATATCCCTCTGATTGACATTTGGGTCGTAGCCAAGCCCGAGTACATGCCATGACCGGTTAAGAGGAAATGTTCGTTTCCAGAATGTGATGAGACCGATTGGTAAAGTTCCCAACTTCCACAATTGTCTATCATGGTTCTGTACACAACAAAAATTTCATTCAAACAATCTAACTTTATGTCTATAAAAAGAAGAGTAAAAAGTATAA |
| CL4384.Contig1_All | PREDICTED: probable galacturonosyltransferase-like 3 [*Vitis vinifera*] | ATGTCTCCACAATGTTACCTCTCGATCTTCTTGCTTTTCATCATTTCCGTACATTTTCCGGCGAATGCCGCCACCGGTGACCTCCCATCATTCCGTGAAGCCCCGGCATTCCGAAACGGCAGAGGTTGTCCCAAAACGACATGGCCCGTCTCTCCTTTACGCAAATCAAACGACGATCTTTCATCGTCCGTCATTCATATAGCAATGACCATCGATGCCACCTACCTCCGGGGTTCAATCGCCGCCGTTTTCTCCGTTCTCCAACACGCCTCCTGTCCGGAAAACATTGTCTTCCACTTCCTCGCCACCCATCGCCGCGCTGACCTCCGCCGTATTATTATCACCACTTTCCCTTACCTCAAATTTCGCCTTTACCATTTCGATACCAACCGCGTCCGTGGAAAGATTTCATCGTCAATTCGTCGAGCACTCGATCAACCCCTCAACTACGCACGAATCTACCTCGCCGACCTACTTCCCTCAACCGTCACTCGTATCATCTACTTCGACTCAGACCTAATCGTCGTCGACGATGTTGTGAAGCTATGGGGAATCGATTTAGGCGGACACGTATTGGGTGCACCAGAATACTGCCACGCCAACTTCACCCACTACTTCACATCAAAATTCTGGACCAATCCGGCCTTTTCAAGGGCTTTTGATGGTCGCCGGCCGTGTTATTTCAATACCGGCGTCATGGTCATAGATCTGTGGAAATGGAGACAAGGTCGCTGCACGGAGAAGCTCGAGACGTGGATGAGAATGCAGAAGAGGTATAGAATATATGAGCTGGGTTCTTTGCCTCCCTTCTTGCTCGTATTCGCCGGAGATGTTGAAGGCGTTGAGCACAGATGGAACCAGCATGGTCTGGGCGGCGACAATCTTGAAGGGTTGTGTAGAGCTCTTCATCCTGGTCCGGTGAGTCTGCTTCACTGGAGTGGCAAGGGTAAGCCGTGGCTGAGATTGGATTCGAAACGTCCGTGTCCACTCGATAGCCTTTGGGCACCCTACGATCTGTTTCGTCACGTATCGTTGTTTTCGGACAGCTGACGCCGTATCATGATTCATGGATGCTCAGTCGATGATGGAGAGAAAGTAGTATGAGAGCACGGATGAGGTCAAAAGTGCGATAACACGGAATTGGAAATTTTGTCTGGTACGGTCGCAATGAACGGTCGTGATTACTTTAACTGGAGGGAGGAGAAGATTGATAATGGTTGTTGCCTGTTGGTGAAATTGTAAAATAATGGTTTCCTCTACTGCACGTGGCAAAGTAGGTGGATATGGGAGATTCCCTGGTCGCCACCATCATGTTATTCTTTTTTGTTATGAAGCTGTAATGCTTGTTTTGGTGATTGGGGGTGTTTGGTTTCTGTTACTCGATGCCCTATGTCCGCACTAGTTTATTTTCAATTCGATTTTGTAAAGTTTTTTTTTTAGTATATTGGGAAATTATAA |
| CL4552.Contig1_All | PREDICTED: probable galacturonosyltransferase 11 isoform X1 [*Vitis vinifera*] | ATGGTGAGGCACTCCTATTTCAGTCGTTTCTTGATCAAATCTGAGTAATGAGTCGTTTTGAATTCTTGTTTTGGTCTTGAATTGTGGAAGGAATTTTAGTAAGATGCGGTTCGAAGGAGGTTTTCGTTTTGGATCTGGGCACTTCTTGGATTGTTCTCCGTTGCAGGATTAGTTCTATTCGTTGTTCATCATAATCACCAGGAGGATCCGGTCAAACAGCCAATACCAGAAAAAAATGAAAGAATTGAGCAGGTTGCCCATCAGGGTTTAAATTTTACTGAAGAAATACTTAGTGCTACTTCATTTGCCAGGCAGTTAGGGGAACAAATGACACTTGCCAAGGCTTACGTCATTATTGCAAAAGAGCATAACAACCTTCAGCTTGCTTGGGATCTCAGTTCAAAGATCAGAAGTTGCCAGCTCTTGCTCTCAAAAGCTGCCATGAGAGAAGAGCCCATCTCACTTGATGAAGCAGAGCCAATTATTAAAAGCCTGTCATCTTTGATTTTCAAGGCACAGCACAACCATTATGACATTGCAACTACAATAATGACGATGAAATCCCACATTCAGGCCCTTGAAGAGCGTGCAAATGCAGCAACAGTTCAAACTACAGTGTTTGGTCAAATGACTGCCGAAGCACTACCCAAGAGCTTGCACTGTCTAAACATCAAACTCACCGCTGATTGGCTTACAAAGTTATCTCTACACAAGCATGAAGAAGAGCAGAGGAACTCTCCCCGACTCGTTGACAACAATCTCTATCATTTCTGCATATTTTCGGATAATGTACTGGCGGTCTCTGTTGTAGTCAATTCTACCGTCTCCAATGCCGACCATCCAACGCAGCTTGTCTTCCACATTGTAACGAATGGGATTAACTATGGAGCAATGCAGGCTTGGTTCCTCAGTAATGACTTCAAAGGGTCGACCATAGAAGTGAAGAACATCGAGGAGTTTTCTTGGTTGAATGCCTCATATGCTCCTGTTGTCAAACAACTCCTTCAAGAAGATTCACTTGCATATTATTTTGATGGGTCTCAAGACTTCAAAGTGGAGCCGAAGTTGCGAAACCCAAAATACCTGTCCTTGTTGAACCACCTACGGTTTTACATACCAGAGATTTATCCTCAGCTTGAAAAGATAGTTTTTCTTGATGATGATATTGTCGTTCAGAAAGAATTAACCCCACTCTTTTCATTAGATTTGCATGGGAATGTCAACGGAGCAGTGGAGACCTGTCTTGAAGCATTTCATAGGTATTACAAGTATCTCAATTTCTCAAACCCAATCATCAGCTCAAAGTTTGATCCTCAAGCTTGCGGATGGGCATTTGGTATGAATGTTTTTGATTTGGTTGCATGGAGGAAAGCAAATGTGACCACACGATATCATTACTGGCAGGAGCAAAATGCTGATAGGACGCTTTGGAAACTAGGTACCCTTCCTCCTGGTCTTTTAACCTTCTATGGACTAACAGAGCCACTTGATCGAAGATGGCATGTGTTGGGATTGGGTTATGATCTGAATATAGACAACCGTTTGATCGAGAGTGCAGCCGTTATTCACTTCAATGGGAACATGAAGCCATGGCTGAAATTGGGTATTGGGAGATATAAGCCTCTATGGGAACGGTATGTAAATCAAAGCCACCCATACCTCCAAGATTGTGTCACTAGTTGAAATGTGCTCTACTTCCCAATATTTATGCAGTTTTCAATATGTTGAAAAACTCTGAAGCAATTATTTTAGAGTCAGAAGTGGGAAAATTTTGGCATTGTTAGGAGAGATTTTCCTTTCCTTTACCTTTTTGTCATTATAA |
| Unigene23624_All | probable galacturonosyltransferase 10 [*Citrus clementina*] >XP_006469845.1 probable galacturonosyltransferase 10 [*Citrus sinensis*] | ATGCCCCAATGATCCATCCAAAAACCCTAAAACATTTTCTTTCATCTGTTACAGATCTCTACAGGAACCCGCGATCTGCAACGGAGGTCGCAAACATTACCTCCAAAACCCCGCTTCTTCAATCGTTACTATTTCAATTCTGATTCTCGCGGAAAAAAAGTTTCCCGGAAATTCTTTTTTCGTGGGAACACCTGTAGGCATGATTTGGATGCATCTGAGCTAGAGTATTTTTTTGACTATCTTTCTGGATTTGGATACGATTTGGCTGATTGTGATTGGGGAAGAGAATGAGGAGGAGAGCGGCGGAGTTTCGCCGGCCTGTGAAGCGGAGGATTCCGAATGTGTTTTGGCTGACATTATGTGGGATAGTAGTCTTGCTCTTCATGACAGAATCATGGAAGGCCTTAACGTGACTGAAGAAATGCTGAGCCCCAACTCAGTGACAAGGCAACTCAGTGATCAAATTTCTCTCGCAAAATCTTTCGTAGTAATTGCTAAGGAAAGCAACAACCTCCAATTTGCTTGGGAATTAAGTGCCCAGATTCGCAACTCACAGATCCTTCTCTCCAATGCCGCAATAAGACGAGCTCCCCTTACGAAACGAGAATCTGAAACTGCAATCCACGACATGGCCCTCTTGCTCTACCAAGCCCAGCAATTGCATTATGACAGTTCAACCATGATCATGAGACTGAAAGCCAAAATTCAAACTCTGGAAGAACAGACAAATTCGGTAAGCGAGAAGAGTTCAAAGTATGGCCAAATAGCCGCCGAAGAAGTTCCAAAGAGTTTATACTGCCTTGGCATCCGATTGACAACCAAATGGTTCAGGAGCCAGTTTTTACATAAGAAGCTCAAGGAAAGGAATCAAATGGAAATAAACTCAAAACTCAAAGACAACAACCTTAACCATTTCTGTGTCTTTTCTGACAACATCCTCGCAACATCAGTCGTGGTGAATTCAACTGCCTTAAATTCGAAAAACCCGAGCTCGGTTGTGTTCCATCTCGTAACCGACGAAGTGAACCATGCGGCAATGAAGGCCTGGTTTTCTCTCAACAGTTTCCAAGGAGTCACCGTCGAGGTTCAAAAGTTCGAAGACTTCACGTGGCTAAACGCGTCTTACGTTCCTGTTCTTAAGCAACTTCAAGACTCTGACACTCGGAACTATTACTTCTCCGGCAACAACGACGGTGGCCGGACTCCAATCAAGTTCCGAAACCCAAAATATCTTTCCATGCTTAACCACCTCCGGTTCTACATCCCCGAAGTCTTCCCTGCGTTGAACAAGGTTGTTTTTCTCGACGACGATGTTGTGGTTCAGAAGGATCTCTCCGGTTTATTCTCGATTAATCTGAACGGAAACGTGAATGGAGCCGTTGAGACGTGCATGGAGACGTTTCATCGGTACCACAAGTACTTAAACTACTCTCACCCATTAATACGAGCGCATTTTGACCCAGACGCATGCGGGTGGGCGTTCGGGATGAATGTTTTCGATCTGGTTGAGTGGAGGAAACGGAACGTGACGGGAATTTACCACTACTGGCAGGAAAAGAACGTTGACCGGACATTATGGAAGCTTGGAACTTTACCGCCCGGACTGTTGACTTTTTATGGGTTGACTGAGCCGTTGGACCCGACCTGGCACATATTGGGGCTGGGTTACACGAATGTGGATCCTCATTTGTTGGAGAAAGGAGCTGTGTTGCACTTCAATGGAAACTCAAAGCCGTGGTTGAAGATTGGGATGGAAAAGTACAAGCCACTTTGGGAAAAGTATGTTGATTATGGTCATCCTTTGTTGCAACAGTGCAATGTGCATTAATGGAAATTTTTGCTTCTCTTCCACGACAATTCTTTTGTATCATCAAGGTCAAGCAGGGTTAGTGGGTTGTAGCAATTGATATTCTAGTGGTTGTAGTATAACATTCTGCATATCGATTATTGCTTAGTTTCCTTAGAAAAAATTTCAGTCAACGTTATTGGGTTGAGCTGTAATTGAATAGCACTAGCTGCAGTTTATAATTGTTTGTTACCAGTGAAGCCTAGCTACTGTTTCTGCAATGTTGGCGATTACAAAAAATTTCGGTCGGTGAAACTTGGGCTGCGTTTTGATATTAATATAATTGGATTTTGTTTGGCCTTTGAGAATCTACTACAGAGTGAATGTATAATTGGGTTTTGTTTGGCCTCCACGGGGTGAATCCGTATCGCCGCCATCATCTTCCATTTATTGATCAAGATCGTCTTCACGAAGCCTCATTCTTTTCTTTTAAGAATGAGAAGAGGAAACTACTCTGTCTACTGCAACATCAATGATACCAGCCTTGAGAAACCTGTATCCTTGAGCGTTGTTTCTTTTGCAAGATTGGTAGCAAAAGAAATTTCAGTGAGAGTATCTACAGTTTTGGTAGGTTTAGTCATCTTCCTCATTCTAGTTCTTCTCTTTTTCCATGTTATTGAGCTATAAGTTAGACTCCATATAGTATCTAATACCAAACACATTATTGTTACTCTCTGTTGAAAGATGATGAGTATTGATGCCCATAACGATATAAGAAGTGGTAATGTAA |
| CL2754.Contig1_All | Acetyl-CoA carboxylase [*Macleaya cordata*] | ATGTATCGAAATTGTCTATCTTTGCTTATGAAAACAACCAAAATCAGGGTTTCCACGACGATCAATTCAATGCTCCCCTTCATAAGACCCCCCTCTCAGTCCTTGTCTCTTGTTTGGCTTGGTCTTATGAGACAGCAAGTTCTGTACATCGTTTCTTAGCAAAATGTCGATTCACTGATCCAATTTGGCCTTTGAGGGATTTGGATAGCTTGAAGATTGAGAATGGCTTCTTTAACTCACTCTCCCATTGAGTATTCTGGTACAAGTGCTTCGGATCATCTCCAGAGCTCAAGCAATGGTGTTAATGGCATTCCTTTGAGAGTTTTAGGAAAGTCTAGGTTGGGTGTCCAGAAGATGGATTTTACTGTTAGTGCAAAGAGGAAAGTGAAGAAGCATGATTACCCATGGCCAAGTGACCCAGATCCCAATGTCAAAGGTGGAGTACTTACTTATCTCTCACATTTCAAGCCATTGAAAGAGAAGCCAAAGCCAGTTACTCTGGAATTTGAGAAACCACTCATGGCCCTGGAGAAAAAGATCATTAATGTACGAAAGATGGCGCATGAAACTGGTCTGGATTTCAGCGATCAAGTCCTGTCATTGGAGGAAAAGTACCACCAGGCTTTGAAAGATTTATATACACATCTTACTCCTATACAACGTGTTAATATTGCCCGACATCCTAATAGACCAACATTTCTTGACCATGTGCTAAGCATTACTGACAAGTTTGTTGAGCTTCATGGAGATCGCGCAGGCTATGACGATCCTGCCATTGTAACTGGTATTGGAAGCATTGGTGGTCAAAGATACATGTTCATGGGTCATCAGAAAGGAAGAAATACAAAGGAAAATATCAAGCGCAACTTTGGAATGCCTACTCCACATGGATACCGGAAGGCTTTGCGTATGATGTATTATGCAGATCACCATGGATTTCCTATTGTCACTTTCATTGACACACCTGGAGCATTTGCAGACCTTAAATCAGAAGAATTGGGCCAAGGTGAAGCCATAGCCTTCAATTTGAGGACTATGTTTGGTCTGAAGGTTCCAGTAGTTTCTATTGTCATTGGGGAGGGTGGCTCAGGTGGTGCTCTGGCCATTGGCTGTGCAAACAAATTGTTAATGCTTGAAAATGCGGTCTTTTATGTGGCAAGTCCTGAAGCATGTGCAGCAATTTTGTGGAAAACCGCTAAGGCTTCTCCAAAGGCAGCTGAGAAGCTGAAGATAACTGCTTCCGAGTTGTGCAAGCTGAAAATAGCAGATGGCATTATCCCTGAACCATTGGGAGGTGCACATGCAGATCCAGCGTGGACATCCCAACAAATTAAAATTGCAATTGTTGAGGCTATGGGTGAGCTCATGAAGATGGATACGCAAGAACTTCTAGACCATCGGATGCTTAAATTCCGTAATCTTGGTGGATTTCAAGAAGGAATACCAGCTGACCCCATAAAGAAAGTAAACATGAAGAAGAAAGACGAGCCAATTTTTCAAAAGGGGATGCGTGGAAAAACCGAATACGAGTTGAGGGATGAAATTGGAAAACTGAAACAGAAAGTCTCGACAGCCAAGGAGTCATCTTCTGAGCCTCCTGTCACAGCTCTAAAGGAGATGATAAAGAAGCTAAAAGTGGAGGTTGACAGGGAATATTTAGAGGCAGTTGAAGCCATGGGCCTAAAGGACAGGCTTCACACGCTTCATGAAGAATTTGTAAAAGCGAGAACTTCACCGAACCAACTCATACATCCAGGTCTGATGGACAAGATTGAGAAGCTTAAAGTTGAATTCAACAAAAAACTAGCCGAAGCTCCTAATTTCGACAACTTGAGTTATAAGCTTGACATGCTAAAGGAAATATCTAAAGCCAAGGAGCTTTCTGAGAAGAATGACAAGGCAATGGCGTTGAAGGAGGAAATCAATAAGAAAATCAAAAAGGTCTTGAATCAGTCCAATGTAAGGAAGAAAATTGAGATGCTCAAGACTAATATTGCAAATGCCGGAGATCCAGATCATGTGATGAAGGTGAAGAAGGAGATAGAAGTGGAGTTGGCTGAGCTCCTCAGGTCTCTGAAATTGGAGGTTGAGGTTGTGGAATCAGAAACAAAAGATCTGGTTGATCAATCGCCGTTGTTGGAGTATAAGGTGGAGATTGAGGAACTGAATGAGGAAATCAATGAGGAGATAGAAAGTGCTATTGGCTCTTCGGATTTGAGAAACAAAATAGAGCTATTGAAGCTGGAGGTGGTAAAGGCAGGAAAGAATCCTGACAAGGAGTCGATGAAAAGAATTGAAGCTTTGAAGCAACAAATCAAGAAAAAAATTATGGAGGCAGTAAGCTCTCCATCATTGATTGATAAACATGAACAACTGAAGATAGAAATAGCTAGAGCCATGGAATCTTTTAGAGGAATTGATGGAAGTTTGAAGCAGGCCGTGAGTTTTTCTTAACGTGGGGGGGATGGAACATATGTTCTGTTTGTTGGCATCGATCAAGTCTGTACATGAATGTTACAAAGTACCAGATTAAGCTGAATAACTTTTTATGTCATCATGAGATATATTTTCGGAATTTGGGGTCAGATTTGTCGAAAGTTAA |
| CL2754.Contig2_All | Acetyl-CoA carboxylase [*Macleaya cordata*] | ATGAAAACAACCAAAATCAGGGTTTCCACGACGATCAATTCAATGCTCCCCTTCATAAGACCCCCCTCTCAGTCCTTGTCTCTTGTTTGGCTTGGTCTTATGAGACAGCAAGTTCTGTACATCGTTTCTTAGCAAAATGTCGATTCACTGATCCAATTTGGCCTTTGAGGGATTTGGATAGCTTGAAGATTGAGAATGGCTTCTTTAACTCACTCTCCCATTGAGTATTCTGGTACAAGTGCTTCGGATCATCTCCAGAGCTCAAGCAATGGTGTTAATGGCATTCCTTTGAGAGTTTTAGGAAAGTCTAGGTTGGGTGTCCAGAAGATGGATTTTACTGTTAGTGCAAAGAGGAAAGTGAAGAAGCATGATTACCCATGGCCAAGTGACCCAGATCCCAATGTCAAAGGTGGAGTACTTACTTATCTCTCACATTTCAAGCCATTGAAAGAGAAGCCAAAGCCAGTTACTCTGGAATTTGAGAAACCACTCATGGCCCTGGAGAAAAAGATCATTAATGTACGAAAGATGGCGCATGAAACTGGTCTGGATTTCAGCGATCAAGTCCTGTCATTGGAGGAAAAGTACCACCAGGCTTTGAAAGATTTATATACACATCTTACTCCTATACAACGTGTTAATATTGCCCGACATCCTAATAGACCAACATTTCTTGACCATGTGCTAAGCATTACTGACAAGTTTGTTGAGCTTCATGGAGATCGCGCAGGCTATGACGATCCTGCCATTGTAACTGGTATTGGAAGCATTGGTGGTCAAAGATACATGTTCATGGGTCATCAGAAAGGAAGAAATACAAAGGAAAATATCAAGCGCAACTTTGGAATGCCTACTCCACATGGATACCGGAAGGCTTTGCGTATGATGTATTATGCAGATCACCATGGATTTCCTATTGTCACTTTCATTGACACACCTGGAGCATTTGCAGACCTTAAATCAGAAGAATTGGGCCAAGGTGAAGCCATAGCCTTCAATTTGAGGACTATGTTTGGTCTGAAGGTTCCAGTAGTTTCTATTGTCATTGGGGAGGGTGGCTCAGGTGGTGCTCTGGCCATTGGCTGTGCAAACAAATTGTTAATGCTTGAAAATGCGGTCTTTTATGTGGCAAGTCCTGAAGCATGTGCAGCAATTTTGTGGAAAACCGCTAAGGCTTCTCCAAAGGCAGCTGAGAAGCTGAAGATAACTGCTTCCGAGTTGTGCAAGCTGAAAATAGCAGATGGCATTATCCCTGAACCATTGGGAGGTGCACATGCAGATCCAGCGTGGACATCCCAACAAATTAAAATTGCAATTGTTGAGGCTATGGGTGAGCTCATGAAGATGGATACGCAAGAACTTCTAGACCATCGGATGCTTAAATTCCGTAATCTTGGTGGATTTCAAGAAGGAATACCAGCTGACCCCATAAAGAAAGTAAACATGAAGAAGAAAGACGAGCCAATTTTTCAAAAGGGGATGCGTGGAAAAACCGAATACGAGTTGAGGGATGAAATTGGAAAACTGAAACAGAAAGTCTCGACAGCCAAGGAGTCATCTTCTGAGCCTCCTGTCACAGCTCTAAAGGAGATGATAAAGAAGCTAAAAGTGGAGGTTGACAGGGAATATTTAGAGGCAGTTGAAGCCATGGGCCTAAAGGACAGGCTTCACACGCTTCATGAAGAATTTGTAAAAGCGAGAACTTCACCGAACCAACTCATACATCCAGGTCTGATGGACAAGATTGAGAAGCTTAAAGTTGAATTCAACAAAAAACTAGCCGAAGCTCCTAATTTCGACAACTTGAGTTATAAGCTTGACATGCTAAAGGAAATATCTAAAGCCAAGGAGCTTTCTGAGAAGAATGACAAGGCAATGGCGTTGAAGGAGGAAATCAATAAGAAAATCAAAAAGGTCTTGAATCAGTCCAATGTAAGGAAGAAAATTGAGATGCTCAAGACTAATATTGCAAATGCCGGAGATCCAGATCATGTGATGAAGGTGAAGAAGGAGATAGAAGTGGAGTTGGCTGAGCTCCTCAGGTCTCTGAAATTGGAGGTTGAGGTTGTGGAATCAGAAACAAAAGATCTGGTTGATCAATCGCCGTTGTTGGAGTATAAGGTGGAGATTGAGGAACTGAATGAGGAAATCAATGAGGAGATAGAAAGTGCTATTGGCTCTTCGGATTTGAGAAACAAAATAGAGCTATTGAAGCTGGAGGTGGTAAAGGCAGGAAAGAATCCTGACAAGGAGTCGATGAAAAGAATTGAAGCTTTGAAGCAACAAATCAAGAAAAAAATTATGGAGGCAGTAAGCTCTCCATCATTGATTGATAAACATGAACAACTGAAGATAGAAATAGCTAGAGCCATGGAATCTTTTAGAGGAATTGATGGAAGTTTGAAGCAGGCCGTGAGTTTTTCTTAACGTGAGTTTCTCCCTTTGGAATCTCAGTTCATGCTCTGGTCATAACCGAATTTCCTTACCCCCTTTTTTGGGATATAACGTGATTTTTTTGTTTTGTTTTCTCTTTTTAGGTGGGGGGGATGGAACATATGTTCTGTTTGTTGGCATCGATCAAGTCTGTACATGAATGTTACAAAGTACCAGATTAAGCTGAATAACTTTTTATGTCATCATGAGATATATTTTCGGAATTTGGGGTCAGATTTGTCGAAAGTTAA |
| CL6370.Contig2_All | biotin carboxyl carrier protein of acetyl-CoA carboxylase, chloroplastic-like [*Cynara cardunculus* var. scolymus] | ATGTCTTGATTGTGTAACTGATGCAAAACCGACTCATATTTATCTAAATTAATAAATTGAACATAATTTCTTTTGGAATTTCAGAAAATCCGAAAAGAAAATTCATTTTGTATAATGCCTTTTGAAAAACTGAAAAAAAAAAAAAAAAAAAATCCATAGATAGTGGAGAAAAACCCACGTCTCCTATGTATAATTGAATTATTTCGAGACTTCCATAGACCAGCCAAGCTAGCAAGTACATATGACACAAAAGATCTACTACAATTTATATCTCAAAATCAGCAGATGATTAACCTTCATGGCTCATCAACAAATAGAAGATACTCTGTGAATAAACAATGGCTTTTCAGGGCTCAATGGCAAACAGAGGCGTATCCACACTAACTGGTTTCCCGTCATCTACAAGTATCTCAACTACGGTTCCCGACTGATCAGATTCAATCTCATTCATCAGCTTCATAGCTTCAATGATGCAAACTACCTGGCCTTTCTGAATCTTGTCTCCTACCTTTACGAAAGGCGGTGTGTCAGGTGCAGGACAACGATAAAATGTTCCCGCCATAGGGCACTTCAAGGGTGGATGAGAGGAAGAGGACTTGGCTGGCTTTGCAGGGGTAGGTGGCGCGGGTGCTGAAACTGGAGGTGGGGAGGGAGGAGAGGGAATGTCCTGGTGAGGTGAAGGCATTTGAAATTGAGGAGGAGGGGATTGCATCATAACAGGAGCAGCAGGTGGCTGTGGCAGAGCTTCCTTTTTCCTTATAAGTAGCTCACAGTCCAGTTGCTTTAGCTCTAGCTCCATAATGTCCCTCGAATCTACTAGCTTAACAAGGTCTGCCACTTGAGTCATAAATGCTGAAATGGATGCTGCATCTGGAACAGAGTTTTTGTGGGCAGGTTTTTCTGCAATTTCTGACTTCTCAGGAACTGGTGCAGAATTTGATGATTTGTTCTCCTCAGGAACTGATGCAGAATTTGATGATTTATCGGTAGCAGCCTTAAGGGAATCGGATTGGATCCGGCCAGACCCCTGTAATCCGAAATAACTTGGCCGATCTTGCAACGACCCAACCATGAATCCAGACTTGGCTTTTGAAATTAACGGAAACGATATCGAATGATGGTGCGGTTGTTGATACTGATTCGGGTTCGGATACGACGCTCCGACCAAAGAGGATTTAGGGCAAGAAGCGGTGAAAGACGCCATGAATTGAAATTGAATCAGCAAAAGCAGCTAAAAAAACCCAACCAGACTGTGAAACCCTAACTCAAGCTGGGAACGAAGAGAGAGAATGGGAGATTTGTAAGAAAAATTGGGCGGAGGAATCGGAGAGACTCGTGAGGAATCCAACGAAATGAAAAGACGAATACAACTGAGGTCATATGTAGGTCGACAGATACTATCGATGTTTACGAGGTGATGCAAATAA |
| Unigene10349_All | acetyl-CoA carboxylase carboxyltransferase beta subunit (chloroplast) [*Passiflora oerstedii*] >AXB37898.1 acetyl-CoA carboxylase carboxyltransferase beta subunit (chloroplast) [*Passiflora oerstedii*] | ATGTAATGTTAATTGGGAAGAGGTGAGAGAAGAGAGTTCCGGATGGAGAAGAGCGTTCCAGATCGAGAAGAGAAGAGAGGATTGCAGGACTTCAAATCTACGGAGGGATTGAAATCGGGATTTAAAGACATAGTACTTGCCTTTAAATCGTTGGGCAAGTTGAAACATGCTAGTTTTGTCAAGAAGCGTGGCTTCCAATCGTGCGGTAAGTTGAATGATGTTAGTTTTGACAAGAAGCTTCAGAGTCGGAGGATCGAGATAGCCCCTGGCTTCCAACCGTGCGGTAAGTTGAAAGATGTCATTTTTGACAAGAAGCTTAAGAGTCCAGAAATCAAGATAGTCCGTGGGTTCCAACCGTGCGGTAAGGGGAAAGATGTTAGTTTTGACCAGAAGCGTGAAAGTAGGAGGAGGAACGAGATAGTCGGTGGCGGCGGCAAATTGAAAGATGCTAGTTTTGACGAGAAGCGTCTGATGGAGGACGCCAAGGAGAGCTGGAGGAGAGAGATAGTCAGCAAGCCGAAATATCGTATTTCAAGTAGCGTGATTGATGAGGAGGAATACCAGGAGAGTTGGAACAAGATGCGTAAGAGTATTGATGGGAGTGAGACTAGTTTTGACAAGAAGTGTGATTCAAGGAGCCAGATTGACGAGGATGAATACCACAAGGAGAGTAAGAGCCGCCTTAGCCAAATTGATGAGTGTGAGACTAGTTTGGACCAGAAGCGTGATTCAAGGAGCCAGATTGATGAGGGGGAATACCATAAGGAGAGTTGTAAGAGCTGGCGTAGCTATATTGATGAGGAGGAATGGAACAAAGATGAGGAGGAATGGAAGAAAGCGCGTAAGAGCTGGGGTAGAAATGTTGATGGGAAGGGGGATGCTTACTATATTGATGAGGAGGAATACCACCACAAGGAGAGTTGTAAGAACTGGCGTAGCTATATTGATGACGAGGAATGGAACAAAGATGAGGAGGAATGGAAGAAAGCGCGTAAGAGCTGGGGTAGCGATGTTGATGGGAAGGGCGATGATTACTATATTGATGACGAGGGTAGCGATATTGATGGGAAGGGGGATGATTACTATATTGATGATGAGGAAGCATCCCAGGAGGCGGTGGCTGCTGAGCCAACTGGTCGGATTCCTATAAGTCGGGTTCCTACATGGTATAGAGGTGGAGGTGGAATAATAGAAGTTAATCATGATATTGGAAGAGATTTTAAACAGTGGCATTTTTGTGTTAGGTGGGGTGTTGATGGTTGTCCTCCCCATGAGCAGATGATTTTAATTTATTTGAAGTGGATTGCTAAAGTTTTGGGGAGCAAGGAGCAGGCGCTTAAGAGTCTTCTTTCTGTCACTGACACCTATTTTATCTGCAAACTCTCTCGAGAGCGAGCTTACTGGTTCATGGGTTTTGACGAGGTTTTTGCCCTGTTTGATGCCATATATGTAATAGATGTTCATGGCAATATCATAGTAGGAGAATATGCTAGAGAAGGGATGCAGTTTTCAGATGAAGATGGTTTCTATCGCCGCAAACTAGATGAAGAATTGCCAGAAGACCCAGTTGCCATTCCCCCATCAAAAATACACGAGAGACTTTCCAGGATATATGCAGGGGTCCCGGAGTAGACTGATTATATTTGACTGGCAGTTGACAGATTATATGTAGGCCTTTGAACGACATTTTTTATTTCTATTTATGTGTAAGCTTCAGAATGACATTTTTTTTCTTTTATTGTGTGTAAGTTTTTGGATGACATTTTTTTTCTTTCTAATTATGATGTTGCTCGGCTGAACGATGAGCTAGTCTAGTTTTAACGTGGACTTGGTATTATTTTAATGGTATTTTGTCTAGATTTAATATGAACTTAACTGAATATTGAACTCCCTCTACTTTAGTCGGAGGTGGTCAGTATCTGTTCAGAGTGGTCACCATTCTTGTGCCCCCTAGCATTGTTAATTTTGCTAGTGGCTGTTCTAA |
| Unigene8775_All | malonyl-CoA:ACP transacylase [*Camellia oleifera*] | ATGCAACTACTAAAAATCTCATCTTTGAAGTACACAAAATGACAAATGGTACATATTATTTCGTTTTCATATAATGCAAGAATACAAGTACATTAAAGCACATAATAACTATTATTATTTTAACTCCCACAAACTACACTACTTAATTTGGCAATGAAGTTAAACAACTCTCAAACTAGATTCAACTCCCCAAAAATTCAAGCTTCAGATTACTTGGCTTGTAGAAAATAACCTCTTCTCTCTACTCTGATAAGTATCCAAATAGTAATCATGAAGTTAACTACTTCTGACTTAAGCACTAATATTCTCAAGTTCAGCTTTTTTGTCCATTCTTTTCACAATCCCAGCTATAACCTTTCCAGGTCCCAATTCATAGCTCTTTTTCACACCCTTAGTTAGAAGAGACTTCACTGTAGTCTCCCATTGAACAGGAGAAGTTACCTGACGTGCCAATATCTTCTTAATTGTATCAGGATCAGCATGTGGATGTGCATCAACATTCGATATAACTGGTATTCGAGGAGTTATAATTTCTGTTGATCCTAATGCAGCTTCCAAACTTGACACAGCTGGCTCCATGAAACGAGTATGAAAAGCACCAGCAACGGCTAGACGCACCGTCATTCGTGCTTTGAATGACTTAGCCTTGGCTTCTAAAACTTCTACTCCTTTCACGCCACCAGATACTGCATAATTTCCGGGACATAAGAAATTTGCAATCTGGACTTTATCATCTTCATCGACTTCTTCATTGGCTGCATCACATAATAGTTGAACCTTTTCTGAATCAAGTCCTATGATACTGACCATGGCACCTTTAGCAGCATCAGAAGCATCCTGCATTGCTTCTCCCCTTAGTTTGACCAACTTGAGGCCATCCTCAAAGCTGAAAGCTCCAGCAAATGCTAGAGCAGTGTATTCTCCTAAGCTTAGACCACATGTGACATCCACAGAATCAATTATTTGTTGTCCTCCCTCACGAACACGCAAGATCTCAACTGCAGCTAAACTTGTGACATAAATAGCTGGCTGGCTTATAATGGTTGAGTCAAGCTTTTCTTTGGGTCCATTTGTGCAAACATCCAGAAGATCAAACCCTAGAATGTCATTTGCTTTCTTGTACAAGTCTGCTGCAGCTGGCACATTTTGAGCCTCTGCACCCATTCCAACAGCTTGTGCACCCTGTCCAGGAAACAGAAACGCAACATTGGGCTTGTAATCAGAAAACAAAGCGTCGTCAACCGAAGCACGTGATCCAACCGATACGCTCATGAAAACTCTACATCTACCCAAATTTCTCGGGACAAAGCTTCGAGCACCATTTCTACAAGTGAAACTTGAGAATTTGTTGCTAAATGAGAGCTTGTTGAGAGAGATCGAAGGGAGAACCAGGGAAGAACTCATGGCGGATGAGAATGAATCAACGAGAGATAGAAGGTAATGTTGTGGAGAGGGAGTGCGTCGGATAAGAGTGGTGAGGGAGAAGGAGGGCATGCATTTAA |
| CL2188.Contig1_All | PREDICTED: uncharacterized protein LOC100264168 [*Vitis vinifera*] | ATGCTGGATTCAAGAAAAAAAGAAAAGATAGCTGCTAAAAAAAATGAAAAAAAAAAACACAAACACAATAGGAAATGGGTCTATTCACTACCAGTAGCCATCAGAAACTCTCCTTCGCAAACAACCTCACCTCCAACATAAGCCTTGCCTTCCATCTTTGCGATTCCAAAACGTTTCTGCAGCTTGATAAGCGTCATTCTCATTACCAACGTGTCCCCGGCAATCACCGGCTTCCGGAATCTCACTCTGTCAACTCCGGCAAAAAAGAAGTTGTCGCGAGAGCCTCCGACTTCAGGTTGCAGCATAACCAAACCTCCTACCTGTGCCATCGCCTCAACCATGAGAACACCAGGCATGATTGGCCTCTCAGGAAAATGTCCAGGGAAGAAGTTATCATTTATGGTTACATTCTTAATAGCAACAGCTGAAACTCCAGGATTGTATTCAATCACTCTATCCACTAGCAAAAATGGAAACCGATGGGGCAAGATCTCACGAATCTGGTTTATATCCATCACCGTCGGAAAGGCCGGAAACCTCTTTTCGATTGGAATCTCTTCGCCTGCGCCATTTTGGGAACAATTGATGACGAAATTCTTTTTCGATTGAATTGAAATGAGATGAGATGCGGGATTGCCGACGGAAATTTGAGAAGACGGCAAAGCTTTGGGTCGATGAGAAAGCGATACACGCGATCTCGGAGGAGCTAATACCGAGTTTGAGAACGCTGTAGCCGCCATTGATTTGTGTGGGTATGGACGGAGCGCAAAGAGACGCAGTGTAGAAGGAGAAGTTAGCGAAGTTGGGTCGGGTAGATCGGTGTATTTAAGGTAAGCGTGTGGGCGAGAATAACGAGGCGAGAAAGCATATGATGTGTAA |
| CL2188.Contig2_All | PREDICTED: uncharacterized protein LOC109007090 [*Juglans regia*] | ATGTTGGATTCAAGAAAATGAGAAGACACTGGTAAAAGAAAAAGAAAAAACAAAAATGAGAAAACTAGGAGATGGATCTATTCACTACCGGTAGCCATCAAAAACTCTCCCTCGCAGACCACCTCACCTCCAACATAAGCCTTCCCTTCCATCTTTGCGATTCCAAAACGTTTCTGCAGCTTGATAAGCGTCATTCTCATTACCAACGTGTCCCCGGCAATCACCGGCTTCCGGAATCTCACTTTATCAACTCCGGCGAAAAAGAAATTCTCGCGAGAGCCTCCGACTTCAGGTTGCAGCATAACCAAACCCCCTACCTGTGCCATCGCCTCAACCATGAGAACACCAGGCATGATTGGCCTCTCAGGAAAATGTCCAGGGAAGAAGTTATCATTTATGGTTACATTCTTAATAGCAACAGCTGAAACTCCAGGATTGTATTCAATCACTCTATCCACTAGCAAAAATGGAAACCGATGGGGCAAGATCTCACGAATCTGGTTTATATCCATCACCGTCGGAAAGGCCGGAAACCTCTTTTCGATTGGAATCTCTTCGCCTGCGCCATTTTGGGAACAATTGATGACGAAATTCTTTTTCGATTGAATTGAAATGAGATGAGATGCGGGATTGCCGACGAAAATTTGGGAAGACGGCAAAGCTTTGGGTCGATGAGAAAGCGATACACGCGATCTCGGAGGAGCTAATACCGAGTTTGAGAACGCTGTAGCCGCCATTGATTTGTGTGGGTATGGACGGAGCGCAAAGAGACGCAGTGTAGAAGGAGAAGTTAGCCAAGTTTGAAGTTGTTGGATCGGTAGATCGGTGTTTTTGTGGGAAGCGTGTGGGCGAAAGTAACGAGGCGAGAAAGCATATGATGTGTAA |
| CL2188.Contig3_All | PREDICTED: uncharacterized protein LOC109013246 [*Juglans regia*] | ATGATTTTTTCTTCCCAGGGCCGGGTATGGGTTTGATATCATATCAAACCCGTACCCTTCCCAGCCCATTGACAACCCTATTTACTAGCATCAAACTGGAAAAACAGAGCCATAAGGTTTGCATTGGGGGGGGAAATGTTGGATTCAAGAAAATGAGAAGACACTGGTAAAAGAAAAAGAAAAAACAAAAATGAGAAAACTAGGAGATGGATCATTTACTACCAGTAGCCATCAAAAACTCTCCCTCGCAGACCACCTCACCTCCAACATAAGCCTTGCCTTCCATCTTTGCAATTCCGAAACGTTTCTGCAGCTTGATAAGCGTCATTCTCATTACCAACGTGTCCCCTGCAATCACAGGCTTCCGGAATCTCACTTTGTCAACTCCGGCAAAAAAGAAGTTGTCGCGAGAGCCTCCCACTTCAGGTTGCAGCATAACCAAACCCCCAACCTGTGCCATTGCCTCAACCATAAGAACACCAGGCATGATAGGCCTCTCAGGAAAATGTCCAGGGAAGAAGTTATCATTTATGGTTACATTCTTAAGAGCAACAGCTGAAACTCCCGGATTGTATTCAATCACTCTATCCACCAGTAAAAATGGAAACCGATGGGGCAAGATCTCACGAATCTGGTTTATATCCATCACCGTCGGAAAGGCCTGAAACCTCTTTTCAATGGGAATCTCTTCGTCTGCACCATTTTGGGAACAATTGATGACGAAATTACTTTTCGATTGAATTGAAATGAGATGAGATCCAGGATTGCGAACGAAAATTTGGGAAGACGAAAATTTTTGGGGTCGATATGGAAGCGATACACGCGAGTTCGGAGGAGCCAATAGAGAGTTTGAGAAAGCTGTGCCAGCCATTATTTTGTGTGGGTGGATGGAGCGCAAGAGACGCAGAGTAGAAGGAGAAGTTAGCCAAGTTTGAAGTTGTTGGATCGGTAGATCGGTGTTTTTGTGGGAAGCGTGTGGGCGAAAGTAA |
| CL2188.Contig6_All | unnamed protein product, partial [*Vitis vinifera*] | ATGCTGGATTCAAGAAAAAAAGAAAAGATAGCTGCTAAAAAAAATGAAAAAAAAAAACACAAACACAATAGGAAATGGGTCTATTCACTACCAGTAGCCATCAGAAACTCTCCTTCGCAAACAACCTCACCTCCAACATAAGCCTTGCCTTCCATCTTTGCGATTCCAAAACGTTTCTGCAGCTTGATAAGCGTCATTCTCATTACCAACGTGTCCCCGGCAATCACCGGCTTCCGGAATCTCACTCTGTCAACTCCGGCAAAAAAGAAGTTGTCGCGAGAGCCTCCCACTTCAGGTTGCAGCATAACCAAACCCCCAACCTGTGCCATTGCCTCAACCATAAGAACACCAGGCATGATAGGCCTCTCAGGAAAATGTCCAGGGAAGAAGTTATCATTTATGGTTACATTCTTAAGAGCAACAGCTGAAACTCCCGGATTGTATTCAATCACTCTATCCACCAGTAAAAATGGAAACCGATGGGGCAAGATCTCACGAATCTGGTTTATATCCATCACCGTCGGAAAGGCCTGAAACCTCTTTTCAATGGGAATCTCTTCGTCTGCACCATTTTGGGAACAATTGATGACGAAATTACTTTTCGATTGAATTGAAGTGAGATGAGATCCAGGATTGCGAACGAAAATTTGGGAAGACGAAAATTTTTGGGGTCGATATGGAAGCGATACACGCGAGTTCGGAGGAGCCAATAGAGAGTTTGAGAAAGCTGTGCCAGCCATTATTTTGTGTGGGTGGATGGAGCGCAAGAGACGCAGAGTAGAAGGAGAAGTTAGCCAAGTTTGAAGTTGTTGGATCGGTAGATCGGTGTTTTTGTGGGAAGCGTGTGGGCGAAAGTAA |
| CL2188.Contig9_All | PREDICTED: uncharacterized protein LOC109007090 [*Juglans regia*] | ATGATTTTTTCTTCCCAGGGCCGGGTATGGGTTTGATATCATATCAAACCCGTACCCTTCCCAGCCCATTGACAACCCTATTTACTAGCATCAAACTGGAAAAACAGAGCCATAAGGTTTGCATTGGGGGGGGGAAATGTTGGATTCAAGAAAATGAGAAGACACTGGTAAAAGAAAAAGAAAAAACAAAAATGAGAAAACTAGGAGATGGATCATTTACTACCAGTAGCCATCAAAAACTCTCCCTCGCAGACCACCTCACCTCCAACATAAGCCTTGCCTTCCATCTTTGCAATTCCGAAACGTTTCTGCAGCTTGATAAGCGTCATTCTCATTACCAACGTGTCCCCTGCAATCACAGGCTTCCGGAATCTCACTTTGTCAACTCCGGCAAAAAAGAAGTTGTCGCGAGAGCCTCCCACTTCAGGTTGCAGCATAACCAAACCCCCAACCTGTGCCATTGCCTCAACCATAAGAACACCAGGCATGATAGGCCTCTCGGGAAAATGTCCAGGGAAGAAGTTATCATTTATGGTAACATTCTTAATAGCAACAGCTGAAACTCCAGGATTGTATTCAATCACTCTATCCACTAGTAAAAATGGAAACCGATGGGGCAAGATCTCTCGAATCTGGTTTATATCGATCACCGTCGGAAAGGCACGAAATCTCTTTTCAATGGGAATCTCTTCGTTGGAACTGTCTGCGCCATTTAGGGAATAATTGATGGTGAAATTACTTGGATTCTTTTTCGATTGAATTGAAACGAGACGAGATCCAGGATAGCGAACGGAGATTTGAGAAGACGAGAAAGCTTTGGGTCGATGAGAAAGCGATACACGCGAGTTCGGAGGAGCCAATAGGGAATTTGAGAACGTTGAGGCCACCATTGTTTTGTGTGGGTGGATGGAGAAAAGTGAGCGAAGACTGAAGAAGTTGGGTCGGGTAGATCGGTGTATTTAAGGTAAGCGTGTGGGCGAGAGTGAGAAAGCATGGTGTTGTAACTTGTAAGAACACATGGGCAGATATCGAATTCACTCTAACGCGCGAAGAAGACAAACGCGACACCGTCGCGACTATGGATCATAGCCGTTAGATCGATCCAACGATAACAACTAATTCACGTTGCACTTTGGTAGTAACCCATAAAGTGGCGGGTTAAGTTATGCAAACGGATCTTCAATATTAAAACGGAGAATCGCGACAACGAGTCATCATTTCTCTCCAAGCAAAAAACATCCGCTCCCACGGAACCACAAGCGAGTCTTCAGTTCTCTCCGACCAAATCGCGTCGTAGTAGTCCGAGGCAGCAGAGCTTTGGCGTGAGAAGAACCTCTTTGGCACAGCCGCACGCCCGCACAGCTCTTTCTTCTCCGTTCAGCAACCTCTTTCAGTCTTTCTTCTCCGTTCAGTGAACCCAGCCAACAGTCCAACCAGGGGAAACAGCAACGTGGTGCAGATCGAATCCGTTCAACAACGTGCAGGCGGTGCACGGTGGTGCAGACCGTGGAGTGGACGGCGGTGCAAGCGTCAGCGGACAGGGAGACCGGATTTCGATTTCAGGTAA |
| CL12103.Contig1_All | enoyl-[acyl-carrier-protein] reductase, mitochondrial [*Manihot esculenta*] | ATGCTAAAAATCACTATTTAACATAATATCATCCAAACAGACACTTGGCTTCAAAGAAACCAAACGCAAGGCATGCGCCCCTCTAGCTTTGATTGGAAATGCTTTTAATTGGGCTTGGCCAAGCCGAGCTTTCACGCCTACAGTCCAAATGGTAAAAAGGGAAACCAAAATCCTGGTCAACCCTTTAGGATTAGAACTTGATGACTTGTTTAGGTTGGCTTCCTAGTTTTCCAAGGGCCTTATCCAGTGCAGTTTGAAACTGGTCAAAAGGGGAAAGCTCCATCTCGTATTTGAGCTTGCCATCTCGCACTAGACCCAAGAGATAATCTATCATTTCTCTGCACTCTTTTGATTTGTCTGAATCCAACCATTTCTGCAACCAGAATCCCCTCAAGGACAAATCCTTAAAAATGAAGGACGAAGTTGATACAGTCACAGGTTTCTTGGACATTCCACCATATGTTACCATAGTTCCACCTTTCCTCAAGAATTTGAGAACCAAGGAAGCATAATTGCCACCAACACAGTTAATTCCCAAAGCAGGCTCGGGTATTTTAGCCAGGAGACCCTTGACATTCTTCACTTGCAATTGGCTTTCAGTATACACTTCATCAGCTCCAAGTTTCTTGAGTTTTTCTTTTACTTCATCAGACCCAACCCTGTAGCATGAACAAGATGATAAAAGAAGCTAGGATATTTCATAAAAAGTTTCTTTTGCTCGAAGAAAATTGCATCTCCGCAGTTATTTTCTGGCCTTTTTCACAGTAGGCTCATCTACCAGTGAGAATATTTATACAAAGTAATAGAAAACCCAAATGTTTCCAGGAGAAACTAAAATTGGCGATGCATAGTGGGATGTATGCTTTAAGATGTGTTAATCAACATAACCATCTGAAAAATAACAGATGTGATGACCACTTAAGTTGATACACTATCTGATAAATAACAGCTGTGATGACCACTTAGAGTTGAAACTGACTAGGAACAGGGATGCTAACTTTAAATTAACAAATTAGCATCCTTAAATTAACAAACTAAATTCACTACTATGAAACTACTCAAATCAACCATTCATTCCGGAATACCTTTCTCAGCAGCACTTATTCTAA |
| CL3685.Contig1_All | PREDICTED: enoyl-[acyl-carrier-protein] reductase [NADH] 1, chloroplastic isoform X1 [*Vitis vinifera*] | ATGATAATGGTTATGGTTGGGCAATAGCAAAATCTCTTGCAGCTGCTGGTGCTGAGATTCTCCTTGGTACATGGGTGCCTGCGCTTAACATTTTTGAAAGCAATCTACGACGGGGAAAATTTGATGAATCGCGCCTGTTACCAGATGGTTCTTTAATGGAGATAACCAAAGTGTATCCATTGGATGCAGTTTTTGACAGTCCTGATGATGTTCCTGAAGATATAAAAATAAACAAACGCTATTCAGCTTCCTCAAATTGGACTGTTCAGGAAGTTGTTGAGTCTGTGAAACAAGATTTTGGCAGCATTGACATCCTTGTACACTCACTTGCCAACGGACCAGAGGTTAGCAAACCTCTTCTGGAGACCTCGAGGAAAGGATACCTTGCAGCCATCTCTGCTTCGAGTTACTCCTTTGTTTCTTTACTCAAGCATTTTGTTCCAATAATGAACCCAGGTGGAGCTTCAATATCTCTCACATACATTGCTTCTGAAAGGATCATTCCAGGGTATGTATACCTTTCTACCATATTTCTATTGTTGATCTAGAGCAACAATTCATCAATGTAA |
| CL4029.Contig4_All | palmitoyl-protein thioesterase 1 [*Rosa chinensis*] >PRQ57354.1 putative palmitoyl-protein hydrolase [*Rosa chinensis*] | ATGCGTCAAATGCCCATAAACACTTACAGATTAACAGGTCCACTTAAGATGGATAAGCGCCCTACCACTCAACAATAAGCAGCGGATCCCACATTATGAATATATTATTTTTTTTTCGTAAGTTGCAATATCAATCATTTTGTGTGATAGCATAATTTTTGGTGGGGGTAATAAATATTTCCAAAAAAAATTATGCAAATCATCCTCCAATCTTTCAGTCTCATTGTTCACTGTATTTTTGATTTTTTTCACATGCCAAAGATGGCACCCAAGTTAATCCAGAGCATGCAAATGAAGCCTATCATCAGTGAGCCCAACCAGTTCCGTAACCAAATTCCAGATTGATAAGAGCCATCTATGCGATGAAGGTTTTGCTGCCAACAGCCGTTTTGATACTTGGTCCTCCAAGTATGGCACAACATACGTTTTCATGTCGCTTTGAGAGATTTCAAGATGATTCCCCGACACGTTAATAAATTTAACTTTTCCAGCTTCATCTAGAGTTTTCAAACCAATCCAGTCCTCAGTGTAGAGTGTGGTCTCTTGCGCAGGCAAAACAGGGTTAAAAGCTCCATCTGGATAATAGCCAAACCAAGATGTTTCCTTGGGTATCAAAATCGTGTCATGCTTGAACATGATAAGCACCAAATTCTCTAGGCTAGAAAACCTTTCCTTATAAGTGGAATTTGCTTTATTTCTGACTTCATTGTTAAGCTTTGGGAGAAATTTACAGCCTTTTAAATAATCAGAAATATCAGTTGGAATTTTCAGATAACCACTGGGTGCCAAGTGTTCCTGGACAAAGTTGCTGTAGATCTCTGACTTGATTAAATTGTCCAAGAGAATGCATAGCAAACCAGATCCACAAAAAGGAATAGAAGCTGTGCCAGCATGAGGACCTCCTAGTGATACGAAATTCTTAACAGGAGGACCGTCATCACAAAACTCAATAACCCCTCGACCTATCATGTTGCCCTGAGAAAGCCCAACAATGTTGTAACCCTCACTGAGTTCAGTCATATTCTTCACCTTCTCACAAGCAATAGCTGTCTGTTCCAGGAGTGGCATAACCCACGAATCCCATTTTCCATCTCCAATTTCTATGCAATATCCTTGAGAACCAGACCAGATGCTTAAATTCTCTGTAAAGCGTGTAACAGCTCCACTGCCGCATCGATCTCCAATGCCATGCAGTACAATGAAAGGGACAGAATATGTGATTGGCGTGAAGATGGTTATAAAGATGAAGGAAATGGTTGGTAAAGGCAGAACCATAGAAGCCATGAGAGTTGCAGAAGCAAAAAATTAAGGGTTTTAGTTGATCATCAAGATGGGAATTAAGCACGGGCGTCTTGATTCCTATAACTTTTGGGTGTTGCTAGGATTTTGGTCTGGGTTTAA |
| CL4347.Contig2_All | palmitoyl-acyl carrier protein thioesterase, chloroplastic-like [*Manihot esculenta*] >OAY26657.1 hypothetical protein MANES_16G064400 [*Manihot esculenta*] | ATGAATAAATCCAACACCACAAGCAGGATTCTACACAGCCAATGCAGACATCATCATCATATAGAGTAATAAGTCTACAAACTTCTCATCCGGGATATATATATTCATGCCCAAATATTACACAGCCAATACAGAAATCATCATCATATAGAGTAATACGTCTACAAACTTCTTATCCGGGATCATATATATTCATGCACATGCTAAGCACCACTTCCTCCTGCTGGAAGAATTTGACCTAATCCCACAAAGTTGTTAACATGCTTGGGTTGCCACTCAGTCCTCCCTCTCACAATCTCACCTTCATCCTCAAGTCGAAGCAAGTGCTAACACTCAAACATTGCCTAAATCAAGGAAGTGGCCAGTGTCAGTACCAACCACCGCAGTTAAGACTGCGGGACGCTGTCCTTTCCACATTCCTTCCTATACTCTAAAGTGATCGCTAAGTCATCGCTGAAATCTCCTGGCTCTCCAAGATTGGCGAGGGAGCACTCTCGAGTATCCATCTAATGTACTTGACATTGTGAACGTGTTGGTTAACATCCAAATCACTCCACCTTGGTTGGAGACTCAATAAATACAAAATCCATAAAATATATTATGGCACATTTAAATGTGAAGTAACATTATTATTTTTAACAACTGCTTGACTTACAATTAAACCTTTGCTAGTATAATATGTTGTATTGTTGTCGAGTTTGGGTAGTTGTTTGGGTAGTTTTCGGTCGTCCTCGTCTACAATTGGACTAGAATTCACAAAATATGGCTCTATTTCACCTCACACTGCTTTTGGAATTTCAGATAATCTTCTCGTCTGTTTATCCATCATTACCCACACTGGAGGCTCTTGTAAGAGTTTCCCCAGTCTTGCAATCACGAATAAACCAATCACGACGCATACCATTTTTTCCAGAAATAATTTCCAGATACATAAATCTGCAAAATAGATCTTGTAAGAGTTGATCCCGGATGAAATCCCAGTGATCAACTTTTAGACATGTGTATGATGATCTGCAAAATACATAAATCAAAAAAATCCATTAATATCTGATGATCAACTTTTAAACATGTGTATGTGTGTCTATGGTTTGTAATTATGATTTATTAGGACCCAACAACTAGAAAATATTTATTTATTAACACCAGGAACTGAACAACAACACAATTGATGTACCTCTAATAGGAACTGAACCCAATAACTAACACACAACTGATGTGTCTCTACTACAAACTGAACCCAGGCGATAGATTTTTGAAATTATATTTATCTAATAGCACTAGTTCGACAGGGGCATAAATGGTTTATTAAGGTCCACAGAATGTGGTGAAAATCAACAATTATAACCCCTATGCATCAAATGTTTACCAATTAAAGGAACAAACACAAATATCGCAAATTTAAGTGGCAATCATCTTAAGCTAAAGGGAATTTCTTTATGTCACAACTGTCAATTAGCCTAAACATCTGAGTAGGAATTTCTAGGCATACTCGTGAAGAACCTACAGGACCAATACATAAATAATAATCATTGCTAACGGGCTAATTAAGTAGAATCAAAGTAAAAATAAATAGATAATTGACCATGAGTTTAGACATCAAAATACTATGCAAATAGAAAGACATCACCAACATGTAGGAAAAATATCAAAAATATATTACGATTAAAAATACACTCCTCTCTAATAATAGATAGTTTTGTAGCTAGTAATATTTAAGAGCTGGAATCCTCATCACCTTGTTGGGAAAAAAAATATATACCAGCTGATGATGATCTTGAAAAAGATCAACAAATATAGGATAACAAATAAATGAAATCAAAATATTATTGAGAATAAATAGTATCTTCAGACATATACTGATTTACATGCTACGGATCTGTATAAAAATAGGACATGATACAAACAGAGAATAGTCATTCTCAAAGAAGCACTTAAAGAGCCAGCCGCCTTTATGGGTGGGTTATTTGCATGTCTTCTGAGACTCGATTTGAACGAAGATCCTCTTAAAGAATGGGTAACTAGAACTGTCGAAGCCTCTGGGTTAACACATGAAGAGATTGGTGTTTAAGGATCTGAACCAGAAGAAAAAGCCCCACAATTGAATGAATTATCTTTGTATACTGAACATGCCCACTAGATGTTTGACAAAATACCATGATGATATAAGAAAATCCTCTTTGTAGTTTATCTATTTGTTATGAAACTTAGTTGAAACATTTGGTTTAATCTGTACGCAATCAAATTAAAAGTGTTGGAATAAAAAGATGAAGATAAACTCAATTTAATATTGAATTAGGAAGGAAAGAAGTAAAAGGAAAACCCTAGATCTAGGAATATTATATCACAAAAACAAACCTGAATCAATATACATGCATAGTGATTTGACAAAAGAAAGAAGAAGGCCTTGACTAATTTTGTGCTCTTGCGTACACCGGGGACCGATTAGGAAGATCCAGAAGAATAAAAGGTAAAATTGACGACCACATAATTAAAAAAGATCTGAGCACTTCGATCATAGTAGAAACTAACAAAGAGGAAAAACAGATCTTCATCTAAAAATGCCTAAAAAACTCACATAACATCTAAAAAACCTGTGAATAATGGAAGTGGAAGAATCTACATGGAAAATAATTAAACCATGAGGACCCGATCATCCGATCTATATTACTGTCTCTAGAAATCTCAGAGACGATCATCCGATCTATATTACTCTCTCTAGAATCTATATTACTCTATCTAGAATTCTCAGAGAGAACTCTGGAGGATCTCAAATTGAAGGATAA |
| CL4347.Contig6_All | plastid acyl-ACP thioesterase [*Vernicia fordii*] >ASL24661.1 fatty acyl-ACP thioesterase B [*Vernicia fordii*] | ATGCTTTTGAGTACCGTGGTAGTGGCTCTTGGAATTCTGCTTACGACCATTAATGGTCCTCATGAGAAGCATGTTGAGGAGCTTCGTCAATATTCCCGGTTTGGGTTATGGTGGGTGGCCCTTGGTGTTGCATCTTCTATTGGTCTTGGATCTGGTTTGCACACTTTTGTCCTTTATTTGGGTCCTCATATTGCCTTGTTCACAATAAAAGCAATGCAGTGTGGTCGAGTTGATATAAAAAGTGCTCCATATGATACAATACAATTAAAAAGAGGTCCATCTTGGCTAGACAAGGATTGCTCTGAATATGGACCCTCATTACATGGTTCACGGATTCCACTTAGCAGCATATTGGCACAGGTCCAGATTGAAGCTATTCTATGGGGAATTGGGACTGCACTTGGAGAGCTTCCTCCATATTTCATCTCAAGAGCAGCACGTTTATCAGGTAGCAAATTGGATGCCATGAAAGAATTGGATTCTTCCTCAACTGAAGACAGTGGAGTAGTAGCAACTCACCTGAAACAAATCAAATGCTGGCTCCTATCTCACTCACAACATTTAAACTTTTTTACAATTCTAGTGCTTGCTTCGGTGCCAAATCCTTTATTTGACCTTGCTGGCATCATGTGTGGACAATTTGGGGTTCCATTTTGGGAGTTCTTTCTTGCAACACTAATTGGAAAGGCAATTATAAAAACTCACATACAGACAGTTTTCATTATCTCAGTTTGCAATAATCAACTTCTTGACTGGATAGAGAATGAGTTAATTTGGGTGCTTAACCTTATACCTGGTGTTGCTTCTGTCCTGCCCAATGTCATCACCAAACTAAATGCAGTAAAAGCCAAGTATCTGGCAGCCCCAGATCCTGTGGTCTCAGATATCAAGGAGACGAAGTGGAATTTTTCATTTACTTCAATCTGGAATGCAGTGGTTTGGCTCATGCTTGTAAACTTCTTTATCAAGATCATCAATGCAACCGCGCAGAGGCATCTTAAGAAACAACAGGAAATTGACTTAGCTGCATTGACGAAAAAGTCATCTTCATCAGATAAGAAGTCTCGTTGAATTCACAATTTTTTGTTTTTTAATCCTCTCTTTTTCCTTGAAAAAGAAAAAAAAAAAAAAAAAAAAAAAAAAGCATGAATAAATCCAACACCACAAGCAGGATTCTACACAGCCAATGCAGACATCATCATACAGAGTAATAAGTCTACAAACTTCTTAACCGGGATATATATATATATATGCATGCACATGCTAAGCACCACTTCCTCCTGCTGGAAGAATTTGACCCAGTCCCACAAAGTTTTCAACATGTTTGGGTCGCCACCCGGTCCTCCCTCTCACAATCTCACCTCCATCCTCGAGTCGAAGCAAGTGCTGACACTCAACATTGCCCGAATCAAGGAAGTGGCCGGTGTCAGTACCGGCCACCGCAGTTAAAGACTGCAGGACGCTGTCCTTCCCACACTCCCTCCTGTACTCTAAAGTCATAGATGAAAGCTCATGACTCTCCAAGATTGGCAAGGGAGCACTCTCTAGTATCCATCCAATGTACTTGACATTGTTAACGTGCTGGTTAACATCCAAATCACTCCACCTAGGAGATAAACCTTTGCGAGCATAATCTGCTGTATTGTCGTCGAGTTTGGGTAGTTTTCGCCCATCTTCGTCCACAACTGGACTAGAATTCACAAAATACGGCTCTATTTCACCTCGCACTGCTTCTGGAATTTTAGATAATCTTCTCGTCTCTTTATTCATCATCACCCACACACTGGAGGCTCTTGTAAGAGTTTCCCCAGTCTTGCAATCACGAACAAGCCAATCACGACGCATACCATTTTTTCCAGATGCATCTACCCAAGTATCAACTTGAACAACATCACCCCAAGTAGGATAGCGGTCAACCAAAACTTGCATTCTAGTGACCACCCATATCAGATTCTTTTTGCACATCTCTGGGGTTGCACCAAAGCCATCACCCAGAAGTCCAGCTGTTTTAACATGGTTGAGAGCCGTTTCTTGTAAATGATTCATTAACGTCTCTATGGATGCTGTACGATCAGCACCTATTTCATAGGACCTAATTGAGAAATTTTGGCGAAAGACAAGACCATCCTGAACAATTCTCCCAAGACCAAATGGGTCCACGAGCATGTCAGGCCGCCTTGGTTTCCAATCAAGCATCATCCATTGCTTCTCTGCCGCCAAAAAGATTGTTGTGATAGCAGCTAGAAGCATGCTCCAATCAGGTAACTGGTTAATGAAAGTCCTTGGGGGAGGAGAATCATCAGTTTTCACAACTTCCGATGGTGAAGTCAGCCCAACTGAGGTACCATTTATTTTGGTTGGAGCTTGGGCATTTGCCTTCACCTGCAACCTACCGGAAGAACCTGATTTCGACTTGATTCCTCCCAAATTCACAGACCCACTTCCATGCTTGTTAGATGACTTGTCACCAGATTCAGCAGAGGGATTAGGAACAGGTAAAAATGCAGCAGCAGCAGCAGAGGCAACCATGATTGAAATGTAATATCGCCCGTGTCAGTCAAGAGTAA |
| CL4347.Contig8_All | plastid acyl-ACP thioesterase [*Vernicia fordii*] >ASL24661.1 fatty acyl-ACP thioesterase B [*Vernicia fordii*] | ATGCAGACATCATCATCATATAGAGTAATAAGTCTACAAACTTCTCATCCGGGATATATATATTCATGCCCAAATATTACACAGCCAATACAGAAATCATCATCATATAGAGTAATACGTCTACAAACTTCTTATCCGGGATCATATATATTCATGCACATGCTAAGCACCACTTCCTCCTGCTGGAAGAATTTGACCTAATCCCACAAAGTTGTTAACATGCTTGGGTTGCCACTCAGTCCTCCCTCTCACAATCTCACCTTCATCCTCAAGTCGAAGCAAGTGCTAACACTCAAACATTGCCTAAATCAAGGAAGTGGCCAGTGTCAGTACCAACCACCGCAGTTAAGACTGCGGGACGCTGTCCTTTCCACATTCCTTCCTATACTCTAAAGTGATCGCTAAGTCATCGCTGAAATCTCCTGGCTCTCCAAGATTGGCGAGGGAGCACTCTCGAGTATCCATCTAATGTACTTGACATTGTGAACGTGTTGGTTAACATCCAAATCACTCCACCTTGGTTGGAGACTCAATAAATACAAAATCCATAAAATATATTATGGCACATTTAAATGTGAAGTAACATTATTATTTTTAACAACTGCTTGACTTACAATTAAACCTTTGCTAGTATAATATGTTGTATTGTTGTCGAGTTTGGGTAGTTGTTTGGGTAGTTTTCGGTCGTCCTCGTCTACAATTGGACTAGAATTCACAAAATATGGCTCTATTTCACCTCACACTGCTTTTGGAATTTCAGATAATCTTCTCGTCTGTTTATCCATCATTACCCACACTGGAGGCTCTTGTAAGAGTTTCCCCAGTCTTGCAATCACGAATAAACCAATCACGACGCATACCATTTTTTCCAGATGCATCTACCCAAGTATCAACTTGAACAACATCACCCCAAGTAGGATAGCGGTCAACCAAAACTTGCATTCTAGTGACCACCCATATCAGATTCTTTTTGCACATCTCTGGGGTTGCACCAAAGCCATCACCCAGAAGTCCAGCTGTTTTAACATGGTTGAGAGCCGTTTCTTGTAAATGATTCATTAACGTCTCTATGGATGCTGTACGATCAGCACCTATTTCATAGGACCTAATTGAGAAATTTTGGCGAAAGACAAGACCATCCTGAACAATTCTCCCAAGACCAAATGGGTCCACGAGCATGTCAGGCCGCCTTGGTTTCCAATCAAGCATCATCCATTGCTTCTCTGCCGCCAAAAAGATTGTTGTGATAGCAGCTAGAAGCATGCTCCAATCAGGTAACTGGTTAATGAAAGTCCTTGGGGGAGGAGAATCATCAGTTTTCACAACTTCCGATGGTGAAGTCAGCCCAACTGAGGTACCATTTATTTTGGTTGGAGCTTGGGCATTTGCCTTCACCTGCAACCTACCGGAAGAACCTGATTTCGACTTGATTCCTCCCAAATTCACAGACCCACTTCCATGCTTGTTAGATGACTTGTCACCAGATTCAGCAGAGGGATTAGGAACAGGTAAAAATGCAGCAGCAGCAGCAGAGGCAACCATGATTGAAATGTAATATCGCCCGTGTCAGTCAAGAGTAA |
| Unigene13141_All | PREDICTED: palmitoyl-protein thioesterase 1 [*Vitis vinifera*] | ATGATCCTTTGTGTATTTGCTTCAGTTACTCTTTGCATTTTCTTGCTTCTTTGTGTCCTAACATCATCACCTCTTCTTTTCAAGGTGACAACTTTTGTTGATGCTTGGGGCTTCAAGTAAGGCACAACATACTTTTTCATGTCACTTTTGGAGATTCCAAGATGATTTCCCGCCACGCTAACGTATTTTACACGTCCAGCATCATCCAACGTCTTCAAACCAATCCAGTCCTCAGTATACAACAGCGTCTGCTGTGGCGGCAAAACTGGCTTGAAGGCCCCATCTGGATAATATCCAAACCAGGAGGTTTCTTTAGGTATTAAAACAGTGTCATGCTCAAACATGATAAGAACCAAGTTTTGTAGACTACTGAACCTTTCCTTGTAAGTTGAATTTCGCTCGTGAGGGAGTTCGTTGTTGAGCTTGGGAAGAAACTTACATTTCTCTAAATAGGAAGGGATATCATTTGGTAACTTGAGATAACCACTAGGAGCCAAATGAGCTTGGACATAGTCAGTGTAGATCTCCGACTTGATAAGAGCGTCCGCTATGATGCACAAAACACCAGTACCACAAAGAGGAACAGAAGCAGTACCAGCATGAGGTCCACCTAAGGAGATAAAATTCTTAACAGGAGGTCCTCCATCGCAATACTCGACAACAGCTCGGCCAATTAAATTACCCTGAGAGAGACCCACGATATTGTAACCCTTACTGAGTTCTTTCATTTTCTTCACCTTGCCACAAACAATTTCTGCCTGTTCCTCAAGGGGCACAAACCAGGAATCCCATGTCCCATCTCCAATTTCTAGACAATATCCTTGTGAAGCCGAGAAGTTGGTCAGGTTCTTAGTGAACTGTTTAAGTCCCCGGTTGGAGCATTGGTCTCCAATGCCGTGAAGTACTATGAAGGGAATAGAGTACGAGATGGGGACGAAGATGAAGGTAAAGATGAAATATGTTGAGAGAAAAGACATGAATTAGGTATCGATCCAAGAACTTTTGCAGGGAATTGATGATTGGTAATTAGGGAAGAAGGCACAGGAAACACTGATGCGTGTAAATCACAGTTATGTTTCTTTTAA |
| CL4185.Contig1_All | stearoyl-ACP desaturase [*Paeonia ostii* var. lishizhenii] | ATGATTATGTGAGAGGAACTTGCAGGGAGTCACTGAAACTGGAACTGAAACTTCTTCCAAGACGGAACTTAGAGCTGAACCTGTCTGTCAAAGATCCAACTGAAAGGAATGACAGGTCCTTGCTTGGCCCTTCCTGCAGCTCTTTCCTCCAACTTTCTAATTCTCGGAGCTAACCCACAAACATAATCCTGAGCCTTTCGTCCTTCCCCAGAAAGTCCAGTTAAATTCTCCACTTTCCATCTTCCAACCAAAAATTCCAATATATCAGCATAGTCCTTTGCAGTATAGACCCCAAGCCTCTGAGCAACGGCCGAAAAGTTTTCAAAAAGGTTGTCATCACGGCCATCGTACATCAGGTGAGCTGGCATTGAGATTTTCTTCCTCATCATGTCAGCAAAAGATACGACTGTCCCATCTGGATCAATCTCAAACAGCTTTTCAACTATCTTGGTGTAGGCTGTTTCATGACGCTTCTCGTCTGAGGCTATTGTGCCACATATTTGAGCCAAATTCAAGTCCCCATGCTCCTTGGCCTGCCTCGCAGTATTTCCATGGGATATGAAGGTTGCCCTCTCTTGAAATGAAGTGTAAATAAAACCCAGGTAGGGACTGTTTTCAGTTCGTGGATCCATTCCTGAACCAATCAAATACTGAATTGTCTTCTCAATTTGCCTCATGTCCACTCGCCCAGACAGGTAAAGGTACTTATTGAGAAGGTCACCATGCCTGTTCTCTTCAGCAGTCCATGCCCTTGTCCAAACTGCCCAAGAAGTAAGACTTGCACCCGTTTCATCTCGGACTCCATCTAAGGTATTCAGCATTGTTTGGTAAGTTGGAAGGGCTTCTTCAGTGACCATATCTCCAACCAAACAAACAAAATAGTCATCTGGAAGCTCTTTTGCCCTCTCCCTTAGTTCCTTGACTTGCTCCTCAAAACCATCAGATGCAGGATCAGGCAGAAAATCCTGTGGTTGCCAACATTTCTCTACGGGCTTGAGGTGAATCAAAATGTTCTGCTCAGCCCAATCCTCTAGAGATTTGAAGATCTCAATCTTTTGTGGTGGCATAGAGTGGGTCACTTGAACATGCACCTCCCGAGGAGGTGTAAAAGGCTTTTTGAGATTCTCAACCTCCTTGGAGCCAGAGCGAAGAGTTGAAGCCATGAAAACTCTAGGAGATCTGATGGTGGTCATTGGTGGGAGGCCAAAAGAAAGTGATTTCTGAGATTGAAAGGGGAGGGGGTTAAGTTTGAGAGCCATAGTTGGCTTTTTGTTTCTCTAA |
| Unigene4178_All | omega-6 fatty acid desaturase [*Paeonia suffruticosa*] >ASW22215.1 omega-6 fatty acid desaturase [*Paeonia ostii*] | ATGCTTTGGATGAAACAAATGGTTGAGAATTATGGTTTCTCTCAAGATACTATGACTCTCTTTTGTGACAATACCAGTGCAGTCAATATTGCCAAAAATCCTGTTCAACATTCTCGCACCAAACACATTGATATCAGACACCACTTTATCCGTGAACTTGTTGAAGATAAGATCATTGCTATGACCCACATTTCTACTGAACATCAGTTGGCTGATATCTTCACTAAGCCTTTGGATATAGGCAGGTTTGAATTTCTCCGCAAATCCCTTGGGATTTGCACTGGGACTTGATCTTGTCCTGGAACTCTCTCGTAGTTTTTCTTTTTCTTCGTTGCTAGTTTATTTTATTTTCTTGTTTTTGCTTTTTACTATTTCTTTTCTTTGGTTGTTAGTTGTTCTTGATCACACATGAGTATTATTTTCCTGCTTAGTGCACTATTTTTACATGATTTGTATTTATGGCACTTGTTATGACAAAAAGGGGGAGAAAGGTTCCCGTTTGTTCATGTTTGCAGGTAAATAAAGAGCATTAACTCAGGGGGAGTTTTCGCGTCAAAGATCTCCAGACTAGCTCCTCAGCATGATTGTCTTTCTTGAACACCGTGATCCAATTTAGGGGGAGTTGCTCTCGCCATTTCATTTCACGGGTTTTGTCATAAAAGTAGCCATTGGGGGAGATTGAAAAAGCATGCTTTGGAATGTCTACTTTTTGCCAAAATTGGAATTGTTCAAGTGTCGGAGTTGCGTGCATCAGAGTCGTCAAGGTCAATGCTCAAGATTGGTTCCATCTCAAGCTCTCCAAAGGTTTATCAAAAGGTTGTCGGAAGACACAGCTTGGCTTCCTGTTTGTAGAGAGGAGTTTGATTCATCACCAGTTCTTCGCAAGGCCCTTATCTATGCATATGGCCCATTTCGGCCTTGGATGTCTATAGCTCACTGGTTGAAATGTCATTTCAATTTGAAGACGTTTAGACCAAACCAAGTTAAAAGGGTGAAGATAAGCTTAGCTTGTGTTTTTGCCTTCATTGCAATTGGATGGCCGTTGATTGTCTATAAGACTGGGATCGTTGGGTGGATTAAGTTCTGGTTAATGCCATGGTTGGGCTATCACTTCTGGATGAGCACTTTCACAATGGTACATCATACAGCACCTCACATACCTTTCAAATCTTCGGATGAGTGGAATGCAGCTCAGGCTCAGCTTAACGGGACAGTTCACTGCAACTATCCTAATTGGATTGAGATCCTTTGTCATGATATTAATGTTCACATCCCTCACCATATTTCTACAAAGATACCAAGCTATAATTTACGGGCAGCTCATCAATCTCTCCAAGAGAATTGGGGAAAGTATCTGAACGAAGCTACATGGAACTGGCGATTAATGAAGACGATAATGACAGTGTGCCATGTTTACCACAAGGAGCAAAATTACATTGCCTTTGACAAGCTAGTGCCCGAAGAGTCTCAACCAATCACCTTCCTCAAAAAAGTGATGCCCGATTATGCTTGATTCATGGGGTCCACTTTTTTTCTTTTTAGGTTCTTTGTTCTCTTTTTCCGAGTCGCGTATTTTCTCCAGTTTCTTAACATCATGTCACTTGTGTTGAAAGTTAAAATCAAGACTCTTCAATTTTAA |
| CL770.Contig2_All | omega-6 fatty acid desaturase [*Paeonia suffruticosa*] >ASW22215.1 omega-6 fatty acid desaturase [*Paeonia ostii*] | ATGAGAGATCAAAGTAAGGGAGATTGATTAACCAATTAGGATAGTTGCAGTGAACTGTCCCGTTAAGCTGAGCCTGAGCTGCATTCCACTCATCCGAAGATTTGAAAGGTATGTGAGGTGCTGTATGATGTACCATTGTGAAAGTGCTCATCCAGAAGTGATAGCCCAACCATGGCATTAACCAGAACTTAATCCACCCAACGATCCCAGTCTTATAGACAATCAACGGCCATCCAATTGCAATGAAGGCAAAAACACAAGCTAAGCTTATCTTCACCCTTTTAACTTGGTTTGGTCTAAACGTCTTCAAATTGAAATGACATTTCAACCAGTGAGCTATAGACATCCAAGGCCGAAATGGGCCATATGCATAGATAAGGGCCTTGCGAAGAACTGGTGATGAATCAAACTCCTCTCTACAAACAGGAAGCCAAGCTGTGTCTTCCGACAACATGTTAGTTTTTGCATGATGCCGATCGTGCTTAAAGCGCCATGGTTCATACGGGTATATCAGAGGCAAAAAGGCCAGAGTTCCAACAATGTCTTCCACTAATTTGTTCCTTGTAAATGATTTGTGAGCACAATCATGACCTATAACAAAGAACCCCGTGACTGCGGTTCCTGTCCATGCCCAAGCCAGAGGGAGTAGATACCATGGGGCTTTTGAAATCATAAAGAGTCCCAATGCATATGAAGAGGCGGATACTAATACTGTCTTCCATGCTTTCACGTCATCGATATCAAAAACCTCTTTTGGGAGAGTATTAACGATATCCTTTAATGTAACATTTTCAGGAAGTGGTTCTCCAATTTGCCTAAATCCATAGCTTTCAGATAATTGCTTTCTATAATCTGCACTTTCTGATGAAGATGGTGCAACTGGAGTCGCCACAGCTTGTACGCTTTTAGTCCTTCTTGTGGAAATTAAACCTTGTTGATGTTTAAATCCTCTTCGTAGAAGGCTTCCCCACTTCAAATAGCATATGCCTGTTGAATTGTGGGGAGTGAATCTATGGCTTCGACTTGGCGTTTGACGGGGGCCCATGAACAGGAAGCTTGAATCTGCAAGTCTGCAAGCCATGTTAGCAGAAATGCAGAATATCCCCGGTTCAAGAAGACCCCCTTTTGAGGATGGAAAGCGTAAAAAGTGTAGCTCTCTGTAATTTCCCTAGCCGCCCTCTTGTGGATAGAAATTTGAAGCCACAACAGCTTTAGTGGTCACATTACCTTCACGTGAGTTGTTCGGTTCTTTCCTTATAAGCCCAAAAATTAA |
| CL770.Contig1_All | omega-6 fatty acid desaturase [*Paeonia suffruticosa*] >ASW22215.1 omega-6 fatty acid desaturase [*Paeonia ostii*] | ATGGGATAACACCGAAAAGAAGATTTAATTTTTGGGCTTATAAGGAAAGAACCGAACAACTCACGTGAAGGTAATGTGACCACTAAAGCTGTTGTGGCTTCAAATTTCTATCCACAAGAGGGCGGCTAGGGAAATTACAGAGAGCTACACTTTTTACGCTTTCCATCCTCAAAAGGGGGTCTTCTTGAACCGGGGATATTCTGCATTTCTGCTAACATGGCTTGCAGACTTGCAGATTCAAGCTTCCTGTTCATGGGCCCCCGTCAAACGCCAAGTCGAAGCCATAGATTCACTCCCCACAATTCAACAGTTTATGCTGACAGGCATATGCTATTTGAAGTGGGGAAGCCTTCTACGAAGAGGATTTAAACATCAACAAGGTTTAATTTCCACAAGAAGGACTAAAAGCGTACAAGCTGTGGCGACTCCAGTTGCACCATCTTCATCAGAAAGTGCAGATTATAGAAAGCAATTATCTGAAAGCTATGGATTTAGGCAAATTGGAGAACCACTTCCTGAAAATGTTACATTAAAGGATATCGTTAATACTCTCCCAAAAGAGGTTTTTGATATCGATGACGTGAAAGCATGGAAGACAGTATTAGTATCCGCCTCTTCATATGCATTGGGACTCTTTATGATTTCAAAAGCCCCATGGTATCTACTCCCTCTGGCTTGGGCATGGACAGGAACCGCAGTCACGGGGTTCTTTGTTATAGGTCATGATTGTGCTCACAAATCATTTACAAGGAACAAATTAGTGGAAGACATTGTTGGAACTCTGGCCTTTTTGCCTCTGATATACCCGTATGAACCATGGCGCTTTAAGCACGATCGGCATCATGCAAAAACTAACATGTTGTCGGAAGACACAGCTTGGCTTCCTGTTTGTAGAGAGGAGTTTGATTCATCACCAGTTCTTCGCAAGGCCCTTATCTATGCATATGGCCCATTTCGGCCTTGGATGTCTATAGCTCACTGGTTGAAATGTCATTTCAATTTGAAGACGTTTAGACCAAACCAAGTTAAAAGGGTGAAGATAAGCTTAGCTTGTGTTTTTGCCTTCATTGCAATTGGATGGCCGTTGATTGTCTATAAGACTGGGATCGTTGGGTGGATTAAGTTCTGGTTAATGCCATGGTTGGGCTATCACTTCTGGATGAGCACTTTCACAATGGTACATCATACAGCACCTCACATACCTTTCAAATCTTCGGATGAGTGGAATGCAGCTCAGGCTCAGCTTAACGGGACAGTTCACTGCAACTATCCTAATTGGATTGAGATCCTTTGTCATGATATTAATGTTCACATCCCTCACCATATTTCTACAAAGATACCAAGCTATAATTTACGGGCAGCTCATCAATCTCTCCAAGAGAATTGGGGAAAGTATCTGAACGAAGCTACATGGAACTGGCGATTAATGAAGACGATAATGACAGTGTGCCATGTTTACCACAAGGAGCAAAATTACATTGCCTTTGACAAGCTAGTGCCCGAAGAGTCTCAACCAATCACCTTCCTCAAAAAAGTGATGCCCGATTATGCTTGATTCATGGGGTCCACTTTTTTTCTTTTTAGGTTCTTTGTTCTCTTTTTCCGAGTCGCGTATTTTCTCCAGTTTCTTAACATCATGTCACTTGTGTTGAAAGTTAAAATCAAGACTCTTCAATTTTAAGGAAGCTTCTTGGAAGTCCACTACTTTCTTGTTGGTTATTTGCTTTTGGTGGTTGTGAGATATAA |
| CL2169.Contig5_All | omega-3 fatty acid desaturase [*Paeonia ostii*] >ARJ54829.1 fatty acid desaturase [*Paeonia suffruticosa*] | ATGGAGAAATCAGTACAAGCCCAAGAAACCTGTAACGAAAGAGAGATGAATCAAGGTACGCACTTCTTTTGAAAAAACTGGTACAAGGCTTAAGGTTTTTTCTTGTTTGAACGTAGTAATTCTTGTTTTATTGATACAAGCCACAAGGCTCAAGCTAAGTTAATGTGCAAAAACTGATGGTACATATATATCTATGAAAGCCTTCTCCTCAAGGAAATCTCTCCCTATTCCTTAGAAGGAGAGATGTCCATTTTGGGTCCTTTGTCATGCGAGTGATGGGGATGGAGCTATAGCCAACCCAAATGAGACTCCATGAGAGTCTCTTTCATGTGCAGCCCTATCGCCGTATGTCAGGGATGGAGCTATAGCCAACCCAGATGAGAAGCCGCTCATGTCAAAAAGAAATTGAATCTTCATTCAATGTCAAAAGATATCCTAGCTAGCTTCGTCTGCATATATGCTGGCTGGCCTAAGGTAAGACAAGAAGTTTGGGTTATCTGCCTTTTTTTCCCATAATATGGTTTCTACTTTCTATTCCAAATTTTTTGCCCAATATGTATGTACACAAAGGATATAAAAAATTTAGAAAGAATTGCTCACCGTCCGGGGGTTGCGTGTACCCAAGGCTGGCCCTATATGGCATTGAGCTTAGGCAACACCCGCTAATCCTGTCGGTGAGCAACCCTTCAATGAGCAATTAGTTATAATTCTATCTAAAGCAACTCAAACCTCACAGCCAGCCCACAAAAACTCTGATATCATGTTAATGTACAATCCGATTCAAGCTCGGGTGGTGGTAGCCTGGTAGGAGAAGGCTAAAAATTATGAAAAACAAAAAAAAAAACAAAAAAAAAAAAAAAAAAAAAAAACTTTTTTTGCTTTTAGAAGAAAACAATAAAGATGAGGTTCTTTTTGGCATTATCTAAGCAGCATTTGTTCTCTTCCGCTTTTTATTTAAATGCAAAAAAACATGAGCATGTGATGATGACATCATCCGATTTATTGTCTTTACATAACCAGTGATGTGGCCTTACAGATGTCAATGCCCATCATCCAAATGGAGTTAACGGAGAAGGAGATGCTACAATAGAAAAAGACGACCACTTTGACCCAGGTGCGACCCCTCCCTTCAAAATTGTAGAGATCCGATCCGCCATTCCAAAGCATTGCTGGGTCAAGAATCCATGGATGTCTCTCAGTTATGTTGTGAGGGATGTCTTTGTGGTGTTTGGATTGGCAGCAGTGGCTATATACTTCAACAGCTGGGCTGTTTGGCCACTCTACTGGGCTGCCCAAGGAACAATGTTCTGGGCTCTCTTTGTTCTTGGACATGATTGTGGCCATGGAAGCTTTTCTGACAACCCTAAGCTGAACAGTGTGGTGGGCCATCTCCTGCATTCTGCAATTCTAGTACCTTATCATGGATGGCGAATCAGTCACAGAACACATCATCAGAACCATGGGCACGTAGATAATGACGAGTCATGGGTTCCGTTGACTGAGAAGGTGTACATGGAAATGGATATTTTTTCGCGTTTCATGAGATACACAGTGCCCTTCCCCATCTTTGCTTACCCTTTCTATCTGTTTGCTAGAAGTCCTGGAAAGAAAGGCTCTCACTTCAGCCCTTACAGTGACTTGTTCTCCACTGCCGAGAAGATGGATGTGATCACTTCAACTGTGTGCTGGCTTTTAATGGTTGCTTTGCTTGCCTGCTTATGCTTTGTAGTAGGTCTTTGCAGCATGCTTAAAATCTATGGTGTTCCTTACTTGATTTTCGTAATGTGGTTGGATTCTGTGACTTATTTGCATCACCATGGCTATGATGAGAAAACTCCTTGGTATCGTGGCAAGGAATGGAGTTACCTTAGAGGAGGGCTTACGACAATTGATCGCGATTATGGATGGTTCAATAACATCCACCATGACATTGGCACCCATGTTGTACATCACCTCTTCCCTCAAATCCCACACTATCATTTGATAGAAGCGACAAAGGCGGCCAAGCCAATTCTTGGAAAATATTATAGAGTGCCAGAAAAATCTGGGCCATTCCCATTTCACTTGATCAAGAATCTAGTAAGGAGCATCAAGCAAGACCACTACGTTAACGACACTGGAGATGTTGTGTACTACAAGACCGACCATGAGCTCTATAAATTTTCGCGCAGCAAGGCCGAGTGACTTTTCAAAAGCCTGTTTGGTTACTTGAGTGGGCTTATTCATGATTTACTTATATTTGAATGAGACCATAGCAAACAATTGATGAGATGGTAGCCTTTAATAAATTTCTAAAACTCATCTCGTCAATTCAAGTATTCATTCGAATGTAGAATAAATTATGAATGAGACTTCAGAAGCTATGGAATGACCAAACAGGCTCAAAAAGTTTACTAGATATTATTTAGGTATTGGAAAGAGGTTACTAATAAGGGTTGCATCATCGTTTTCTTTTGTGGACTGTATGCAGTTGTGATGAAGAGAACCACTTTTATGTGTAATCTTGTACAAATAA |
| CL11925.Contig2_All | PREDICTED: probable glycerol-3-phosphate acyltransferase 8 [*Vitis vinifera*] >RVX10014.1 Glycerol-3-phosphate 2-O-acyltransferase 4 [*Vitis vinifera*] | ATGACCAAATTTCATTAATGATCAATCACATAAATAACATGGAAATTTTTATGTAAAAATTCTAAACTGATAATAGAGTGACAAATAGACCGTTACATAAACGTTTCATATAAACCTCTCCACTGTTTTACTAAATCCAATGACCAAAATACCCTCTCCCGAAATCACGAGATGAAGATGTTCCTTACCCTTTCTTGGCATACATTGATTCCACCTTCCCATCATTTCCTCCCAACAGCATATACTTATCCTTCCTGGTTAATCCAGTGCACTCAAACCCAAGAACCCCACCCAACACCTTCTGCACATGATTCGCCACCTCAATCGGCGACTTTCCGCCAGCCTTACACGTCATCTCTTCTGGCAGCCGATCAAGAAACGTGATTTCATAAGTGGGCCTCGGGTTCATAAAATAGAAATACGCGTCCCAGAACTTGACCCCACGAACCGTCGTCCCATTAAACATGTTTTGCTTCACATTAACTGCCACCGGTACGATCCGATCACTCAACTCTGCGAATAAAGCACTGAACCGAAGCAAATACGGCTCGCGACAAGTGGTCCCTTCGGGACAAACCACTAGATCACCCTTTTGGAGGATGGCTGAGATCCGGGCCGCGTCCGCTTCACGATCACGGGTCAAGGCAACGGCTGGGATGGGTGATAGGAATCTTGAGAGACGGCTTACGCTGTATGTGACACACGAAACTTTGCGTCCGAGTGCGATGGCGATCACGATTGGATCGAGGGCTGTACGGTGGTTGCATACGTAAAGGTTTCCAGGGTGGCCGGCGGAAGGAGCTGGCGGTGGAGTGCCGCGGATTACGAGGTTGATGCCGAGGGCCGGGTATGTGTAGCGTACGATTCGTTCTGGGAGGGGAAGGTTGAAGTAGACGCGGAATATGGAGAGGATGAATCCGAATGGGAGCCATATGAAGGTGATTAGGGCATTAAGGGGGTCTGGGCGCTGAACTAGGCGGCCGTCGTGGAAGATGATCCGATTTTTGAGTCGCTCGACTGGTACTGGAGTTGCTGATTTGTTAGGTAGTACCATGTACCCCTCCTTGCAAATTGACATAAAATCATGATCAGTTTTCCGATCTCCGAGCCCAATATCCGGCGACTCTTCCCCAAATTCCTTCAAAATCGCCAACCTCTTCTGGTCTCCCACCAAAACCCCCGACTTCTTCACAAACCCTGTCGCCTTCTTCGTCTTTGGATCCACCTCGATTTCGGTGCCCAGCACTTTATCAGCACCCAAAAACTCTTTCACAAAATAATCAACCATCACAATTGGATTCGCCGTCACCACTACTCTCCTCCCACACTTCTCAAACACCTCCCAACTCTCCCGCCTCACATCCGCCGCATAAAACCTTGGAAGCACGGCGCGGGATGCGAGCTCGATGTCACGGATCCTGAGTCCCGCGAAGGAGATGTAGATTAGGATTTGGATGCCGAGGGATTCCGAAACGAAAAGGTAGGATAGTACGATGAGAGGGAACGAGAGGAGGAGTATGAGGCCGCGGAGAAGGCTTCCGGCTTCGACGGCGATGAGCATGAAGTAAGGGAACGAGCTTCTGGAAATGAGGAGCGTGCCGTCGAGGTCGGCGGCTACTGACTTTTTTTCACCACCGGAAACGTTGCATGTCGTCACTGACGGGAAGTTTCTGCTTGGTTTTGGCGTCATGCCTCCGCCGGAAAGCAAATGGAACTGTTCTATTTCTCTAAAACTTGTATTTTCTCTCTCCCAATAA |
| CL8660.Contig2_All | PREDICTED: 1-acyl-sn-glycerol-3-phosphate acyltransferase 1, chloroplastic [*Vitis vinifera*] | ATGCGGGAGGATATTTCCTTGAAATTGCACCCCTGAACCAAAAAAGAATGAAAAACAAAAGGCTTTGGGGAAGTAGAAAATATTACGCATTCGTATAAGTTAGTGTAGATTCAAACGAAAATTCTATAGCGTTTACTAGAGTTAGAGACAACAAGATCAGTTAGGTACCATCTTCGGATTGCCACATTTCGTCCATGGAAGTCTCTTCTCAACTGAAATTCCACCGTTCTTTCATCCACACATCTGCTTTCTTACATCGCTTCAGTGGGAAAGAATCGAGCTTTTTGTTTCCATCGTCTTATTTACCGTTGTGCACTAATAAAGGACTTTGTATAGAGCGACCCACATATAGTCGGGACATTATGAGAAATTCAAACGATTATAGCATAAGCAGTCCAAACCAATGTTTTGGTGTACCTCGGCTCTATTTCACCCCAAAGAAACTGTCCAGATATATTGTTGCAAGATCTGAACTTGCTGGACCGGGGAATCCTGGTGCTGCCCAGCCTTTATCAGAGTTTCAATTGAGCTCTAAACTTAGAGGAATATGCTTTTACTCCGTTTGTGCTTTTCATGCCATTTTCCTGTTTGTGATGATGGTGGTGGCGCATCCTTTTGTGCTTTTGTTGGATCGATACCAAAGGAAAACTCAACACCTTATTGCCAAAATTTGGGCAACTTTATCTGTCGCTCCATTCTTTAAAGTTAAATTTGAAGGATTGGAAAATCTGCCTGCACAAGATATTCCCGCTGTTTATGTTTCCAACCATCAGAGTTTTTTAGATATATATGCTCTTCTAATTCTTGGGAGAAGCTTCAAGTTCATTAGCAAGACAGCTATTTTTTTCATTCCCATTATAGGATGGGCCATGTCTTTTATGGGTATTATTCCTTTGAAGCGCATGGACAGCAGAAGCCAGTTGGAGTCTTTTAAGCGATGCATGGATCTTGTAAAAAATGGAGCATCTGTTTTTTTCTTCCCAGAGGGAACTCGGAGTAAAGATGGGAAATTAGGTCCTTTCAAGAAAGGAGCATTTAGCCTTGCTGCGAAAACCAGAGTGCCAGTGGTGCCAATTACTCTTATAGGAACGGGACAAATAATGCCTGCGGGAATGGAGGGTATATTGAATTCAGGATCTGTGAAAGTTGTTGTTCACAAGCCTATAGAAGGGGATGATCCAGACGTACTGTGCCGCGAAGCTAGAAACATTATTGCAGATTCGCTCAATTCTCAAGGCTAACAAATTCGGTGTTAAAAGTTGTTTTTGTTTTCTACATCTCAATTTTGAATCCTTTGGTGTGGATAGAAGTATAATCTGAGAGATTTTGTAACGCTGAGCTAGTGCTCCATTCATCGAGTCTTGCTGGAACCTACTCTTAGTTTGCTTATAACACCGTGACTGGTTTATTTCTTTCACTTCCATAATGAATTTTTATGGTTATTGTCTTTTACAAATTACATTCTAGTGTTTTATTGAATATAAGTAGTGGGAGCGTGGAGAGATAA |
| Unigene338_All | PREDICTED: 1-acyl-sn-glycerol-3-phosphate acyltransferase 3 [*Theobroma cacao*] | ATGCGCCCACGCACAGATCATCATCCATAGACCCGTCCCCCCCGTTCTCTCTACAATTCACTCTTTCTCTCTCTGCAACAATTGCGTGCGACTGATGCTATGGCGATCCCAGCTGCAATTGTCATTGTTCCTGTGGGAATTCTCTTCATTCTCTCAGGCCTTATCGTCAATCTCATTCAGGCAGTTTTCTTCATCCTTGTTCGTCCGCTGTCGAAGAATATGTACAGAAGGATTAACAAAGTAGTAGCAGAATTGTTATGGATGGAACTCATATGGCTCTTTGACTGGTGGGCAGGCATCAAGGTTGACCTTTATGTGGACTTGGACACTTTTCAGTTAATGGGTAAAGAACATGCCCTTGTCATATGCAACCACAGAAGTGACATTGATTGGCTTGTTGGATGGGTCTTAGCTCAGCGCTCAGGTTGTCTTGGTACTGCACTAGCTGTCATGAAGAAATCATTAAAGTTTCTCCCGATCATAGGTTGGTCAATGTGGTTTTCTGATTATGTCTTCCTGGAAAGAAGTTGGGCCAAAGATGAAAGGACATTAAAGTCTGGTTTTCAGTGTCTGGAGAATTTCCCTCGGCCTTTTTGGTTGGCTATTTTTGTAGAGGGGACTCGCTTTACACCAGCAAAGCTTTTAGCTGCTCAAGAATATGCTGCTACAGCAGGGTTGCCTGTTCCTCGGAATGTTTTGATTCCTCGTACCAAGGGATTTGTTACAGCGGTAAGTCATATGCGTTCGTTTGTTCCAGCGCTTTACGATATCACTGTGGCTGTTCCTAAAAATCAGCCTTCACCCACAATGCTGAGAATGTTTAGGGGTCAATCTTCTGTGGTAAGCGTGCACATCAAGCGCCGTTCTATGCATGAATTGCCAGAAAGAGAAAGTGACATTTCAAAGTGGTGTAAAGATTTATTTGTGGACAAGGATGCCTTTTTGGATAACTTTCTTGCCACAGACACATTTGGTGATCAAGAATATCAAGGCATTGGTCGACCGAAGAAGCCTTTGTTTGTAACTATAATTTGGTCATGTCTCCTAATCGTTGGTGCTATCGAATTATTTCGATGGTCTTCACTATTTACCCTGTGGGAAGGTATTGCATTTGCAGCAACTTTGTTGGTCATTGTTACAATTGTCATGCAAATCCTCGTTTTATTTACTCAGTCAGAACATTCCACCCCTCCCAAGGCAACCCCGCAGAGCTCCTTGAAGGAGAAGCTGCTTGTTAAATGAGCTGATTAATATTCAGTTTTCTGTATGTATCATTAGCAGAGCCTTAACACTTTGTATGCATACTAATCCATTTACTGCTCCTTGCCACTCCTAACAAAAAGAAATGACAGTGTAAAGAGAAATTGGGCCGGGGGATGTTCTTTTGACTGATCCTACACTTGTAA |
| CL10660.Contig1_All | Phospholipid/glycerol acyltransferase [*Trema orientale*] | ATGAATCAACGAATTGATGTTCTCTAGTCTCAACTATAAAGACCAAGAAACTTAAATAAAATAAATAAAATAAAAACGCCAAGGCAGATGACACATAATACTACACATATCACCGAATTCACAAAAACAGCTGTGTTGATCAAGCGACTGATTTACAGAAAAATATTAAGAAAAACAAGCTCTGTGATTTTTAATGTTGTTTGTTTTGTCCCGACCCTGACGATGACTCTTCCCCTTGGTTCTTGGTCTTTGCTGGGGCAATCTTGGCGGGAGTTGAACGCTCCGCCTGTGAGAAGAGAATCAAGAAGTGCATGAGGAAAGTGATTAAACCTACTACAGTTGCCGAGATTGCCATTCCTTTCCATGAGGATAGAAGTGATGACCACTGGAGGAATTTTAGAGTTCCGAAGAAAAGAAAACATGACCAAGAAATAACCACCACAAGTGACTTCTTCGGCCGACCGGTGTCATGAAGTTCTTGGTCACCAAAGGTATCCTCGGCTATATGTTTGTCCAATAATGCATCCTTGGCCACAAATATATCTTTACACCACTGTGCAACTGCATCGTCAGTTTCAGGCAAATCCTTCATCAAATGTCGTTTGATGTGCACGTGCACCACAGAGGATTGCCCCTTGAAGAGTCTTAGCATTGTAGGTGAAGGCTGATTTTTAGGAATAGCTATTGTTCCGTCATAAATGGCAGGAACAAATGAACGCATGTGGCTAACTGCTGAAACAAAACCCTTTGTACGAGGAATCAAAACATTTCTCGGAACAGGTAATCCTGTTGACGTTGCATATTCTTGAGCTGCTAAAAGCTTTGCCTGCGTAAAGCGAGTTCCCTCAACAAAAAGAGCCAACCAAAAGGGTCGAGGGAAATCCTTCAACCGTCGAAGACCCAACTTTAATGTACTTTCATCCTTTGCCCAATTTCTTTCCAGAAAGAGATACTCAGAAAACCACATCGACCAGCCTATGACCGGAAGAAATTTTGATGACTTCTTCATTACAGCTAGTGTGCTGCCAAGGCAACCTGAGCGCTGAGCCAATACCCATCCAACAAGCCAATCAATGTCACTTCTGTGGTTGCATATGACAAGGGCATGTTCTTTACCCATTAACTGAAAAGTGTCCAAGTCCACATAAAGGTCAACCTTGATGCCTGCCCACCAGTCAAAGAGCCATATGAGTTCCATCCATAACAATTCTGCTACTACTTTGTTAATCCTTCTGTACATATTCTTCGACAGCGGACGAACAAGGATGAAGAAAACTGCCTGAATGAGATTGACGATAA |
| CL10660.Contig2_All | Phospholipid/glycerol acyltransferase [*Trema orientale*] | ATGAATCAACGAATTGATGTTCTCTAGTCTCAACTATAAAGACCAAGAAACTTAAATAAAATAAATAAAATAAAAACGCCAAGGCAGATGACACATAATACTACACATATCACCGAATTCACAAAAACAGCTGTGTTGATCAAGCGACTGATTTACAGAAAAATATTAAGAAAAACAAGCTCTGTGATTTTTAATGTTGTTTGTTTTGTCCCGACCCTGACGATGACTCTTCCCCTTGGTTCTTGGTCTTTGCTGGGGCAATCTTGGCGGGAGTTGAACGCTCCGCCTGTGAGAAGAGAATCAAGAAGTGCATGAGGAAAGTGATTAAACCTACTACAGTTGCCGAGATTGCCATTCCTTTCCATGAGGATAGAAGTGATGACCACTGGAGGAATTTTAGAGTTCCGAAGATAAGAAAACATGACCAAGAAATAACCACCACAAGTGACTTCTTCGGCCGACCGGTGTCATGAAGTTCTTGGTCACCAAAGGTATCCTCGGCTATATGTTTGTCCAATAATGCATCCTTGGCCACAAATATATCTTTACACCACTGTGCAACTGCATCGTCAGTTTCAGGCAAATCCTTCATCAAATGTCGTTTGATGTGCACGTGCACCACAGAGGATTGCCCCTTGAAGAGTCTTAGCATTGTAGGTGAAGGCTGATTTTTAGGAATAGCTATTGTTCCGTCATAAATGGCAGGAACAAATGAACGCATGTGGCTAACTGCTGAAACAAAACCCTTTGTACGAGGAATCAAAACATTTCTCGGAACAGGTAATCCTGTTGACGTTGCATATTCTTGAGCTGCTAAAAGCTTTGCCTGCGTAAAGCGAGTTCCCTCAACAAAAAGAGCCAACCAAAAGGGTCGAGGGAAATCCTTCAACCGTCGAAGACCCAACTTTAATGTACTTTCATCCTTTGCCCAATTTCTTTCCAGAAAGAGATACTCAGAAAACCACATCGACCAGCCTATGACCGGAAGAAATTTTGATGACTTCTTCATTACAGCTAGTGTGCTGCCAAGGCAACCTGAGCGCTGAGCCAATACCCATCCAACAAGCCAATCAATGTCACTTTTGTGATTGCAGATGAAAAGCGCATGCTCTTTACCCATTAATCGAATGGTTTCTTCATCTGTATATAGTTTGACCTTCATCCCGGCCCACCAATCAATGAGCCACACAAGCTCGAGCCACAGCAATTCTGCCACTACTCTGTTAATCCTTCTGTATGTGCTTTTTGAAAGTGGCCGAATGAGGACAAATAAAACTGCCTGAATGAGATTGACCACAAGGCCTGAGGTAAAGAAGAAGACAGACAAGGGTACTACCACCACTGCTGCTGCAATCGCCATTCCCAGACCCAATCCCTTTGAGGGTGAAAATTAATTTTTATATATGATGAGAAGTTTTGCAGGTAGGGAGAAGCGTTTGGAGTGAGAAGAAGGGAAGGTATTTGAGAGTGGGGGATGGGGAGAGAGAGAAATTGATATATAGAATAATTGATCGATGATTTAAATAATATAATATTGTTTATCGATGTTTTCAGAGATGTTACTGAGATGCACTGCCCGCTTTAA |
| Unigene23957_All | Phosphatidic acid phosphatase/chloroperoxidase, N-terminal [*Cynara cardunculus* var. scolymus] | ATGGAACCACCACCACAACCAGAACCCATTATTCGGCGTCTAATCAACCTCGACACTACCGTCTCTCTCCATCTACACACAGTTACCCAACCTCTTATCCCCCGCCCTCTCCTCAAAACCCTAGAAATCTCCGGCGATGGCCGCTTGTTCTTCCCCGTCACACTTTCTCTCCTTCTATCTCCTCTCCCCTCTTCCACTCCCCTCCTCCGACCCTTCCTCGTATCCCTTTTGCTCGGGTCTCTCATGGACCTCCTTCTGGTCGGACTCACTAAGCACCTCGTCCGCCGATCTCGCCCTATCTACAACAAAGGCATGGATCTGGTTTTCTCCGTCGATAACTGGTCCTTCCCTAGTGGGCACTCCTCCAGGGTTTGCTTTATCGCTGGCTTTTTGTATCTGTGCTCACCGGAGATCAGAGAGGCTTTGGCGCAATTGAGGTCTGCCGACGGTGTAGGCTATTACTTCAATGAGAATTTTCGATTGTTCATTTGGGCTACTAGTTTGTGGGCAGCGGCGACTTCGGTTTCTAGGGTTTTGCTTGGCCGGCATTTTGTTTTTGATGTGGTTGCGGGGGCGTGTTTGGGTGTTCTCGAAGCTCTGTTTGTGTTCCATTATCTGAAATACTAGATTTTGAGGTAAGAACATGATAGGAAGAAGTGTTTTTTTCTGATTTCTCATTATGGTTTATCGTTATCTACTGAATTTACTAAAGCAACTGTAGAGATTGTGAGGTGAAAATTTCAAGCTATTGTTATCATACTTTGTACTGTGGTGCTTAATGTATTTAGTTTCCATTGTAATGTAATTAACCTTTTATTGGATTTGCATCTGTTTTGTTGCATAATTAA |
| CL4895.Contig2_All | Diacylglycerol acyltransferase [*Parasponia andersonii*] | ATGATTTGTTACACTAATTGAGATACACGTTGATTTCTTCAACTCTTAATTTTGGAAGAGTTTGAGGGAATTTACAAAGTTTTCTTGTTGCTTAATGCTATGCTCTACATCATGGGCTTTCGTCTTGGTCACTTATATTTAGCCCACCACAATTTAACACTAGTTTGCTGAATTAATTAATTATACCAATTGTATAGCAGAAATAAATAGATAAATGCACATACATGTATGTATATGAGCAGGTTCCCCCCACGCCATTTATCAAGGACAATGACTCGTGAAGAGTGAGTATAAACTGCTCACAATATTTTCAATTCAAGATCGGCATGGCCAACCCGTGTTTTGTGCTTTTCAAACAGATTCTTAAGTGCTTCAACGAACTGACCGTGTACTTGAATCACCTCTTCCATAGTGGGTTGTGGATTTTTCTTAAGCTCAATGGGTCTTCCTACAACCACATGCATTGGATGCCGAAAGGGGATTGGAGATCTGGCCAAAACAGAAAACTGGAACTAGGGGTAGGCCCATCTCCATGGCAATACGAACAAATCCTCTTCTCGTCTTCAGGAAAACATTCTCAGAACCATGCTCCATATGAAATGTTTCTTGCACTCCACCAGGTATTACGATGCAACTATAACCAGCTGACAAAAGGGAAATAAAACCTTTCTTTGTTGCAGGTGCAAGACCCAACCATGACCATATATGCCTCAAGAATGGTGTGTAGAACACAGCACTACTTGCGAGGACCTTTATTTTTGGGAGAGGCATAAAACCCGTAAGGTTGGCAAGTGCAACGACACCAATTGGCAAAACTGAATGGGGCTCATAACCAAAGACATATGCACGATTTGGATCAAAGGCCTCTATATCCTCCACATGTAGAGTCACCGGAAAATAACCACAGGCATGCTTACATATATACCTGGATAACCTTCGACCTACTTTGCTGCTATCGTCAATTGGTATAACCATTAAAAATATAA |
| Unigene22497_All | diacylglycerol acyltransferase [*Paeonia suffruticosa*] | ATGCTTTTGTTGCACCAACCTTTCAACTATAAATGTGGCAAGAGGGAAAAATTAGGAGAGATGGACTTCACATGAGAAGGGGCAAATATCTCAAGGATTTTGAACTAAACCAAAAATCAGACCTAATTAACAAATCATACTTCATGAGATTCTCGATAATAAGATGGATGTTGGCTGATGCTGGTAGGGTTGATTGCTATTGCGGGTCACTAGCGTCGCTCCATTGAGGTTTTTTAGGCGGTGACCTTCTAGTTTGAGTCCGCTTGAATGTTTCCTGGTTCTGCAGGCTTGAGTCGCTTGCAGGTTCACTGGTTCTGCTGGCTTGAGCCCTGCAAGGGCCTGCCTGGTAGCCTCGAATGCCTTACAATGATGGCAGAATGAGAGGTCGAGTGTGAAAGGGATATAA |
| Unigene23779_All | diacylglycerol acyltransferase [*Paeonia suffruticosa*] | ATGGATCTTATTTTCTTTATCTTAAACCACACTTTCTAACTGCAGCTCTTTTTCTTGGCATCAACGGCGATCTCGAATTCGCCCAAAAGTGTAGTCACTACATTAACTCCTCAGCTCAAGTCAGATCCTAACTTTTTTATTCGGAGGAGATTGAGTGTTAGGACTGTTGTTGATAACTCGAACGAGGTTAAATCTGAGAATTCGGGTAAAAATGAATCGCTGGACGATAGGGACTCAGTCGGGGCTGGCGATGATAGGGTGGAACCGAGGAATAATGGAATGTACAGTGGTGATAAGATGGTAAATGGTTAGAATCGAGCGATAGATTTCACCTTGAAATTCGCTTATCGGCCTTCTATTCCAGCTCATTGGAGAGTGAAAGAGAGTCCTCTCAGTTCTGACGCCATTTTCAAACAGAGCCACGCATGTCTTTTTAACCTTGTATAGTTGTGCTTGTTGCTGTAAACAACCGTCTTATTATTGAGAATCTCATGAAGTATGGTTTGTTAATCAGGTCTGGTTTTTGGTTTAGTTCAAAATCATTGAGAGATTGGCCACTTCTCATGTGTTGGTACTGCTTCCTCTTCTTTTTCTTTTATGTATAAATGAATCAAGTGCTCGATGTAGATTTTATAAGAGTGTAATTATTATTTAA |
| CL3264.Contig2_All | Phospholipid:diacylglycerol acyltransferase [*Actinidia chinensis* var. chinensis] | ATGTCAACAGTAACTGATTATCAAACCTTTTTGAACTCCCAAACTTTGATGTGCCTTCGAAATTAGAACTAGAGGCTGGAACAATTTCACTAAATTGAGGCGGACAGAAACACGAGAAGAAACTAGACACGGTGCTCATAAGGATGAACAATTGAAGAAAGAGGAAATTTGAACCCATAATAAAGATCAAACACCACCTTAAGGAAAATCAAACAAAAAGACCAGCAATTAAGCAACAATGGGATCGGGCAGAAGTCAGAGCTTAATATGAGGGTTGATAACAGATTAAATTACATAACACGTCAATAGAACACGAGCATGTACCTTCCAACATTATAGTTGTAAATTGATCTTCTCCGACCACTTGAATATCTCAGTGTACACTTTATCACCTCCCAAGTCTTCACCTTTAGCCCCAGCTGCTACCCTTAAAACATCCTCAATCAATTGAAAATTCCCCATTATATCCACATGTGCACCACTCTGTGTGCCCCGACCTTCTAAAAGATTGGCTGGGGGAGCGTGATCGTACTCCCGAATGTAAGTACGAATCCCAGAAGGATTGAATCTGGTTTTTCCACGCCAAGCTTTTGCACACATGAAGCCTGCACTTAAAACAGGCACTGTCTCGTCCCCGTCGACTGTGTAGACTCCATCTTTTAGGCAGCTGTCTTCCTCTCCACCATCAGCTGATGTATCGATCTGAAATGGAATGTAGCATTCAGCAGAAGGAGATTGCTTGTAAACATACGCTCTTTCAGTCGGGATACCAACTCCATACATCGTGAATATTTCAATGTCAGGAGCATTTGGTAATTTTGTTTCCAAAGGATTTGACCAATATTTATAGTGGTTGTATTTTGGATCATCCAAGTTGTCAGCAATTCCATACGAAAAATGAGCACTGCCGCGTGCCATCATTTTGGGAGCAACAAAACCGAGCAGATCCACAATTGACCCAACAGTGTAAACTTTGTAGTCGACAACAGCTTTAATCCCTTCAATTCCCATGTCATGGTACTCCGTCCACACATCACGACAGCTTGTGTTTGCAACATTGTTACCCTTAACGGCATCCCTAAATTCAATCCTCTCAATCTCAGATGACCGTGCCTTGGCTAGATCTTTCCCAAATGAAATAATCCTTCCAAAATTAACACTTTCTGTTCGAGAAACCAGACTTCCAGTCTCATTTTGCCCTACAAGCTGACTATCATTGTTTCTTTGCTTTTTCTTGCTAGGAACGCAACATTCTTCAGGTGACCAGTCAAGACCACCCCAAATAGTATCGCCACCTTTTGGTATCATGGACATGGTTGAATCCCATGTGCGGCTCATTCTCATCACATGTTGCAATGTTTGTAGCCGAAATAAATCATTATCCAAAAAGCTTGGTGTAATGGCTCTGGCAACTGCTATATCTTTGGCTTCAGCAGAGAAAAGCCCAGAAACGGCCTTTGGAACACCTAAAAATGGTCCACCAATGTTCATCACTGCCTTTATATGCTTAGAACACCAATCTGATCCACCCCCACCACCCACTGGAGCCGGAACCTCAACCCACTTCATAAAATGCAAAAAGTACAAAACGCCCATGGAATGTGGAATGATAACTGCTTTTTTTCCACTTGTAGCAACCATCAGTTCGATATTACTTTTTATTCTACTAAGTGTTTGGTCCCGCGCCTCAGTGTTCTGAAATGAGAGTCTCCAATCATAGGCAGCCATATACATGGTTTTTTCCTCATACCCAATGCGAGCTAAATTGGCAATAAGAACTGCCCATACAAAATAGCCCGGAGCGAAGTAATCTGCTGCTACGAGTCCAGTGACAGGCCTGACCCTTATACCAGGAGGATCCATTCCAGTCTCATTGTCCAGTGACATATGCTCTACCCAGCATAAAGGTCTTTTATAAACTTCTCCAAATGTGCCCCCCCAAAGCCGCTTCCGAAACAACCCATCCGCACACTGATGCCCTTCCCATAATTCAAGCCCACCCGTCACAATACCCGGCACGAACACCACCGGATGCTTTGCCGTCAACCCTTCTTTACGCAATTTCGCACCTGGAGGGTCTGGCAACGGCCCTGTGATGGCCTCCGTGACGTACTGAGGGAACGAAGCCGGCATCGCATTGTATAGAAACAAGAGTACCCACCACACCGTACATATAAATCCTACGAACCAACAGCAGCTATCGATACATGACCACTTATTTGGGTCTTCAACTTTCTTCTTCGGAGCAGCCTTCTTGATTTTGCTGCTCTTGTCGTTGTCTTCTCCACTATTTGTTGAAATCGAAGTTTTGTTATCAGTTTCAGATGTTTTTCTCCGTCGCATTAATGGCATATCTTTAAATGCCTTTGGTTGAGAAAAAATTATCAGAAGGTGAGGGAGTCGAAGAATATAATCTTCTTCCCCTTTTCTTTTATCTTTTACTCTTTCTTCGGTAGGAATAATCTGGAAATGAGGAATTGACTGTGGTCGTTGATTGTGCTGCTAA |
| CL3264.Contig3_All | Phospholipid:diacylglycerol acyltransferase [*Actinidia chinensis* var. chinensis]  Phospholipid:diacylglycerol acyltransferase [*Actinidia chinensis* var. chinensis] | ATGCCATTAATGCGACGGAGAAAAACATCTGAAACTGATAACAAAACTTCGATTTCAACAAATAGTGGAGAAGACAACGACAAGAGCAGCAAAATCAAGAAGGCTGCTCCGAAGAAGAAAGTTGAAGACCCAAATAAGTGGTCATGTATCGATAGCTGCTGTTGGTTCGTAGGATTTATATGTACGGTGTGGTGGGTACTCTTGTTTCTATACAATGCGATGCCGGCTTCGTTCCCTCAGTACGTCACGGAGGCCATCACAGGGCCGTTGCCAGACCCTCCAGGTGCGAAATTGCGTAAAGAAGGGTTGACGGCAAAGCATCCGGTGGTGTTCGTGCCGGGTATTGTGACGGGTGGGCTTGAATTATGGGAAGGGCATCAGTGTGCGGATGGGTTGTTTCGGAAGCGGCTTTGGGGGGGCACATTTGGAGAAGTTTATAAAAGACCTTTATGCTGGGTAGAGCATATGTCACTGGACAATGAGACTGGAATGGATCCTCCTGGTATAAGGGTCAGGCCTGTCACTGGACTCGTAGCAGCAGATTACTTCGCTCCGGGCTATTTTGTATGGGCAGTTCTTATTGCCAATTTAGCTCGCATTGGGTATGAGGAAAAAACCATGTATATGGCTGCCTATGATTGGAGACTCTCATTTCAGAACACTGAGGCGCGGGACCAAACACTTAGTAGAATAAAAAGTAATATCGAACTGATGGTTGCTACAAGTGGAAAAAAAGCAGTTATCATTCCACATTCCATGGGCGTTTTGTACTTTTTGCATTTTATGAAGTGGGTTGAGGTTCCGGCTCCAGTGGGTGGTGGGGGTGGATCAGATTGGTGTTCTAAGCATATAAAGGCAGTGATGAACATTGGTGGACCATTTTTAGGTGTTCCAAAGGCCGTTTCTGGGCTTTTCTCTGCTGAAGCCAAAGATATAGCAGTTGCCAGAGCCATTACACCAAGCTTTTTGGATAATGATTTATTTCGGCTACAAACATTGCAACATGTGATGAGAATGAGCCGCACATGGGATTCAACCATGTCCATGATACCAAAAGGTGGCGATACTATTTGGGGTGGTCTTGACTGGTCACCTGAAGAATGTTGCGTTCCTAGCAAGAAAAAGCAAAGAAACAATGATAGTCAGCTTGTAGGGCAAAATGAGACTGGAAGTCTGGTTTCTCGAACAGAAAGTGTTAATTTTGGAAGGATTATTTCATTTGGGAAAGATCTAGCCAAGGCACGGTCATCTGAGATTGAGAGGATTGAATTTAGGGATGCCGTTAAGGGTAACAATGTTGCAAACACAAGCTGTCGTGATGTGTGGACGGAGTACCATGACATGGGAATTGAAGGGATTAAAGCTGTTGTCGACTACAAAGTTTACACTGTTGGGTCAATTGTGGATCTGCTCGGTTTTGTTGCTCCCAAAATGATGGCACGCGGCAGTGCTCATTTTTCGTATGGAATTGCTGACAACTTGGATGATCCAAAATACAACCACTATAAATATTGGTCAAATCCTTTGGAAACAAAATTACCAAATGCTCCTGACATTGAAATATTCACGATGTATGGAGTTGGTATCCCGACTGAAAGAGCGTATGTTTACAAGCAATCTCCTTCTGCTGAATGCTACATTCCATTTCAGATCGATACATCAGCTGATGGTGGAGAGGAAGACAGCTGCCTAAAAGATGGAGTCTACACAGTCGACGGGGACGAGACAGTGCCTGTTTTAAGTGCAGGCTTCATGTGTGCAAAAGCTTGGCGTGGAAAAACCAGATTCAATCCTTCTGGGATTCGTACTTACATTCGGGAGTACGATCACGCTCCCCCAGCCAATCTTTTAGAAGGTCGGGGCACACAGAGTGGTGCACATGTGGATATAATGGGGAATTTTCAATTGATTGAGGATGTTTTAAGGGTAGCAGCTGGGGCTAAAGGTGAAGACTTGGGAGGTGATAAAGTGTACACTGAGATATTCAAGTGGTCGGAGAAGATCAATTTACAACTATAATGTTGGAAGGTTAGTTGGAAACATTGCTATAAAGAAGAATTAGTGATCCAACTCCGTTTATATCTACTTCATTAAAATTGCCTTGTCAATCAACCGTGTAAACTGGCAAGAGTGCTGGTCTTAGTCACAACTAGTTTGTACTCTTCTCAAGAGAACCCTGAGAAAGGTTTAAAGATTGTTTATTCAATAAAATGGGGGGTTTTGGGTTAA |
| CL3264.Contig4_All | Lecithin:cholesterol/phospholipid:diacylglycerol acyltransferase [*Trema orientale*] | ATGATTTTTAATAGATTAGGGTCCTAATTCTGACTATTAACACCTCTGATCATCTCTATCAGAGCATTTAAAGCGGCTCTCTTTAATTTATGTGTGAACAATTATGTCCAACTTGAGACGCAGAAAGGGCTCTGAACCCGATAAATTTCCCGAGTCAGAATCTCTGATTAATGAAGATGAGAAAAAAGAGGAGATAGAGACAGAGACAGAGGAGGTGAAGAAACAAGAGGTGGTGAATAAAAAGAAGAAGAAAAAGAAGTGGTCTTGTATTGATAGCTGTTGTTGGTTTATTGGCTTTATTTGTTCGGTTTGGTGGTTCTTGTTGTTTCTTTACAATGCTATGCCGGCATCATTCCCTCAGTACGTCACGGAAGCCATTACAGGACCTTTGCCAGACCCGCCCGGCGTCAAATTGAGGAAAGAAGGGTTAACGGCCAAGCACCCAGTGGTTTTCGTACCTGGGATTGTTACAGGTGGACTTGAGTTGTGGGAAGGGCACCATTGTGCTGAGGGATTGTTCAGAAAGAGGCTTTGGGGTGGTTCATTTGGAGAACTTTACAAAAGACCCTTATGTTGGGTTGAGCACATGTCGTTGGATAATGAAACTGGATTGGATCCCTCTGGTATAAGGGTTAGGCCTGTATCTGGACTTGTTGCTGCCGATTACTTTGCACCAGGTTATTTTGTATGGGCAGTTTTAATTGCTAATTTGGCTCGCATAGGGTATGAGGAGAAGACCATGTATATGGCCGCATATGATTGGAGACTTTCATTTCAGAACACTGAGGTCAGGGACCAAAGTCTAAGTAGAATAAAGAGTAATATAGAGCTCATGGTAGCTACAAGTGGGAAAAAGGTGGTGGTTATTCCACATTCTATGGGCGTTTTGTACTTTCTGCATTTTATGAAATGGGTTGAGGCACCAGCTCCAATGGGTGGTGGGGGTGGATCAGATTGGTGTGCGAAGCATATAAAGGCAGTGATGAACATTGGTGGACCATTTTTGGGCGTTCCAAAAGCAGTTTCTGGACTTTTTTCTGTCGAAGCCAGAGATATTGCCGTTGCCAGGGCATTTGCACCAGGCGTCTTGGATAAGGATGTATTTGGTCTTCAAACCTTTCAACATATGATGCGTATGACACGTACATGGGATTCAACCATGTCAATGATACCAAAAGGTGGGGAAACGATCTGGGGTGGCCTTGATTGGTCACCAGAAGGAGGCTATGACTGTGGTGGGAAAAAGGCGAAGAACAATCATAGTCGGACCACAGGCCAAAACGGGCTGGGTTCAACTAAAAGTGTGAATTATGGAAGAATTATATCATTTGGGAAGGATGCAGCTGCTGTACATTCGTCGAAATTGGAAAGGAAGGATTTTAGGGAAGCTGCCAAGGGTCATAATCATGCAGAGACGGCATGCCATGATGTATGGACAGAGTACAATGACATGGATGTTGAGAGCTTCAAAGCTGTCTCAGATTATAAAGTTTACACTGCAGGGACGATTTTGGATCTGCTTCATTTTGTTGCTCCCAAGCTGATGGTACGTGGAGGTGCTCATTTCTCATATGGTATCGCTGACAATTTGGATGACCCGAAATACGAGCACTATAAATATTGGTCAAACCCCTTGGAAACCAAGTTACCATATGCTCCAGGCATGGAGGTTTATTCTCTGTACGGAGTTGGAGTTCCAACTGAAAGGGCGTATGTTTATAAGTTAACTAGTGGTGGGGCTGAATGTCACATCCCATTTCAAATAGACACCTCTGCAGAGGGTGGAAGTGAAGCTCCGTGTCTAAAAGGAGGGGTTTTTTCCGGTGATGGAGATGAAACTGTTCCTGCTTTAAGTGCAGGTTTCATGTGTGCAAAAGGTTGGAGAGGAAAAACCCGATTTAATCCTTCGGGGATTCCTACTTATGTAAGAGAATATGATCATGCACCTCCAGCTAATCTTCTGGAAGGCAGGGGAACACAGAGTGGTGCTCATGTTGATATAATGGGGAATTTTGCATTAATTGAGGATGTTTTAAGAGTAGCAGCAGGAGCTACAGGAGACGGGTTGGGGGGTGATCGAGTTTTCTCTGATATCTTCAAGTGGTCTAATAATATCAACTTACAACTCTAAGGTTCACAGGATGAAAGGATTGCACATATATCTACAAGCCGAGAGTGTTGTATTTAAAAGCTAAGAAATGAGGAGGAGAAGGGATTCTAGAAACAAAGAATCAAGATTTTTGTTGTTATCCACTTGTATTCACAGTTTCACATATAGTGTTTATTGTTTTGGGAAGCACGAATCTGATTCACGTGTTCGGCATCCCATGTTATACCAGGTTCCTCTTATTGTAGGTAAATTTTTTGATATTATATTTATTTTTTAATTTATAA |
